# Towards the Inhibition of Protein–Protein Interactions (PPIs) in STAT3: Insights into a New Class of Benzothiadiazole Derivatives

**DOI:** 10.3390/molecules25153509

**Published:** 2020-07-31

**Authors:** Matteo Mori, Ettore Gilardoni, Luca Regazzoni, Alessandro Pedretti, Diego Colombo, Gary Parkinson, Akira Asai, Fiorella Meneghetti, Stefania Villa, Arianna Gelain

**Affiliations:** 1 Department of Pharmaceutical Sciences, University of Milan, via L. Mangiagalli 25, 20133 Milano, Italy; matteo.mori@unimi.it (M.M.); ettore.gilardoni@unimi.it (E.G.); alessandro.pedretti@unimi.it (A.P.); fiorella.meneghetti@unimi.it (F.M.); arianna.gelain@unimi.it (A.G.); 2 Department of Medical Biotechnology and Translational Medicine, University of Milan, via C. Saldini 50, 20133 Milano, Italy; diego.colombo@unimi.it; 3 Department of Pharmaceutical and Biological Chemistry—UCL School of Pharmacy, University College London, 29/39 Brunswick Square, London WC1N 1AX, UK; gary.parkinson@ucl.ac.uk; 4 Center for Drug Discovery—Graduate School of Pharmaceutical Sciences, University of Shizuoka, 52-1 Yada, Suruga-ku, Shizuoka 422-8526, Japan; aasai@u-shizuoka-ken.ac.jp

**Keywords:** benzosulfamides, structure-based virtual screening, STAT3-SH2 domain, cysteine binder, diversity-oriented synthesis

## Abstract

Signal transducer and activator of transcription 3 (STAT3) is a validated anticancer target due to the relationship between its constitutive activation and malignant tumors. Through a virtual screening approach on the STAT3-SH2 domain, 5,6-dimethyl-1*H*,3*H*-2,1,3-benzothiadiazole-2,2-dioxide (**1**) was identified as a potential STAT3 inhibitor. Some benzothiadiazole derivatives were synthesized by employing a versatile methodology, and they were tested by an AlphaScreen-based assay. Among them, benzosulfamide **1** showed a significant activity with an IC_50_ = 15.8 ± 0.6 µM as a direct STAT3 inhibitor. Notably, we discovered that compound **1** was also able to interact with cysteine residues located around the SH2 domain. By applying mass spectrometry, liquid chromatography, NMR, and UV spectroscopy, an in-depth investigation was carried out, shedding light on its intriguing and unexpected mechanism of interaction.

## 1. Introduction

Since the discovery of the relationship between the constitutive activation of signal transducer and activator of transcription 3 (STAT3) and malignant tumors, the validation of STAT3 as a target has been supported by a large number of studies. Various compounds with STAT3 inhibitory activity have been reported in recent years, including potent inhibitors such as Stattic, S3I-201, and erasin [1,2,3,4]. Nevertheless, these molecules are mostly at the experimental stage, and only few are in clinical trials [5]. Hence, the challenge is the discovery of new selective candidates with high potency and in vivo activity. Since a direct inhibitory approach should be preferred, we addressed our efforts to the disruption of STAT3 homodimerization, an essential step in its activation, targeting the protein–protein interactions (PPIs). The large surface area of STAT3 and the chemistry of these interactions [6], which are very different from those of better-known targets such as enzymes and G-protein-coupled receptors, could represent significant hurdles, although a number of successful examples have demonstrated that it is possible to overcome them and develop PPI modulators [7]. Since the binding of a ligand to a hot spot should compete with the original protein partner of the PPI, thus resulting in disruption of its function, the aim of our ongoing studies [8,9,10,11,12,13] is the identification of small molecules that are able to bind the Src homology 2 (SH2) domain of STAT3, preventing STAT3 dimerization and thus its activation. With this purpose, in the present work, some compound libraries were analyzed by means of a structure-based virtual screening performed on the STAT3-SH2 domain (see the Molecular Modeling section). Among the candidates that reached the best scores in the docking study, we decided to focus our attention on the compounds that bear a sulfone moiety, which is a common feature in some STAT3-SH2 inhibitors reported in the literature [1,2].

In order to support the docking results, the selected compounds were either purchased or synthesized, and their ability to interact with the STAT3-SH2 domain was evaluated via an in vitro binding test (AlphaScreen-based assay) at 30 µM; the most interesting results are reported in Table 1. Hence, we identified the benzothiadiazole derivative **1** as a promising hit compound that carries a chemically accessible moiety for further development.

**Figure 1:**
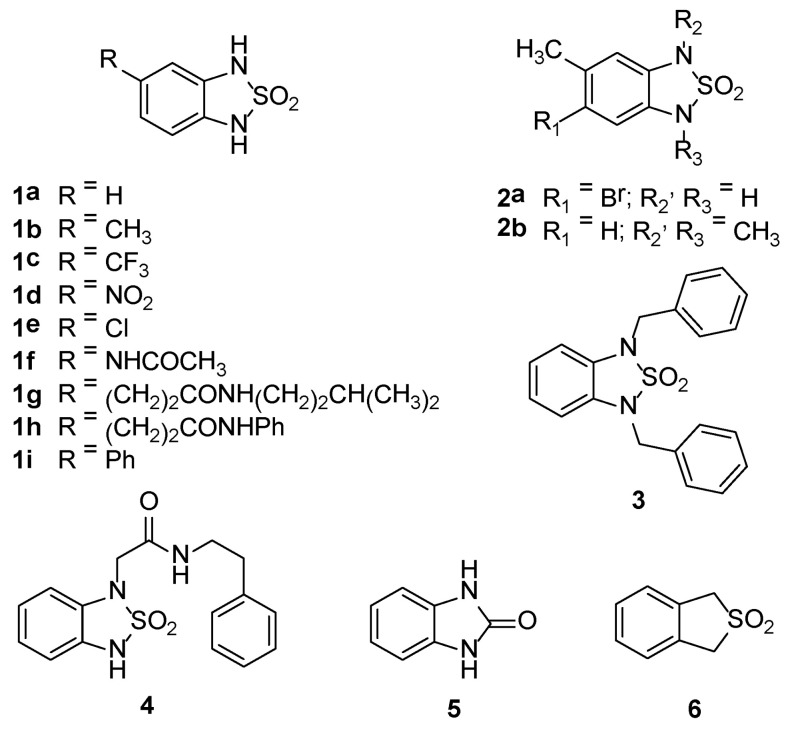
Library of 5,6-dimethyl-1*H*,3*H*-2,1,3-benzothiadiazole-2,2-dioxide (**1**) derivatives.

The significant inhibitory activity exhibited by 5,6-dimethyl-1*H*,3*H*-2,1,3-benzothiadiazole-2,2-dioxide (**1**) prompted us to fully characterize it by means of an X-ray analysis. Moreover, we designed and synthesized a small library of derivatives bearing different substituents on the aromatic and thiadiazole rings (**1a**–**i**, **2a**,**b**, **3**, **4**). Furthermore, imidazol-2-one (**5**) and thiophene dioxide (**6**) moieties (replacing the thiadiazole ring (Figure 1)) were assessed to develop a preliminary structure-activity relationship (SAR) on this class of compounds.

For the synthesis of **1a**–**i**, we employed an adaptable methodology [14] that also considered a diversity-oriented approach using suitable monoprotected intermediates. For derivatives **2a,b**, **3**, and **4**, our synthetic strategy was planned to allow for the introduction of different substituents on compound (**1**) by the direct alkylation of the sulfamide moiety.

Herein, we describe the in silico-based rational design, synthesis, characterization, and interaction with the STAT3-SH2 domain of new inhibitors bearing the benzosulfamide scaffold. Furthermore, since several STAT3 inhibitors have been reported as irreversible cysteine binders (e.g., Stattic and S3I-201) [15,16,17,18], we decided to evaluate the activity of compound **1** versus the cysteine residues located around the STAT3-SH2 domain (Cys468, Cys542, Cys550, Cys687, and Cys712) and to shed light on its mechanism of interaction by MS, UV, LC, and NMR studies.

## 2. Results and Discussion

### 2.1. Molecular Modeling—Design and Chemical Space Exploration

In order to identify new direct STAT3 inhibitors, molecular modeling studies were carried out. In particular, a virtual screening approach was applied to compound databases with the aim to select a series of candidates endowed with the best binding scores. Therefore, we performed a structure-based virtual screening, choosing STAT3 in complex with a DNA fragment (Protein Data Bank (PDB) ID 1BG1) [19] as a model and employing a set of databases of small molecules, namely: AKos (544391 molecules), ChemPDB (4009), ChemBank (2344), Kyoto Encyclopedia of Genes and Genomes–Compound (KEGG, 10005), National Cancer Institute Anti-HIV( NCI’s Anti-HIV, 42689), and National Cancer Institute (NCI, 15237).

Each molecule was ranked on the basis of the docking score (interaction energy of AutoDock 4) [20] of the best solution. In this way, a list of compounds was obtained in which the first and the last molecules were, respectively, the best and the worst interacting ones. This list made the next step of the analysis easier because only the first 500 compounds for each database were further considered. Hence, the best molecules were classified on the basis of the most recurrent moieties: (e.g., sulfonic acids, sulfonamides, sulfamates, phosphates, and diketones; Tables S1–S5, Supplementary Materials). This kind of clustering was performed by encoding the moieties into SMARTS patterns and performing queries on the databases of the top-ranked poses, as implemented in the VEGA ZZ program. These results were very interesting because the functional groups included negatively charged atoms, such as oxygens, which can mimic the phosphate moiety of Tyr705 (pTyr-705), upon interaction with positively charged amino acids. A further selection was performed based on the commercial availability, synthetic accessibility, and size of the molecules. In particular, small molecules were privileged because, in the future, they may be included in peptidic derivatives to improve their selectivity. Therefore, drawing inspiration from compounds reported in the literature [1,2], we decided to focus on the cyclic sulfonamide moiety, ultimately selecting compounds with a molecular weight lower than 300 Da. The biological activity of several candidates belonging to the NCI collection was evaluated by an AlphaScreen-based assay, and among the most interesting hits, compound **1** emerged as a promising lead for further development (Table 1). The analysis of its docking pose (see Figure S1) revealed that compound **1** may interact with the SH2 domain through the insertion of its sulfonamide moiety into the polar pocket lined by Arg-609 and Lys-591; moreover, it showed that the compound occupies most of the pocket volume normally filled by pTyr-705 in the dimerized STAT3. Starting from this qualitative analysis of the binding mode, it seems reasonable to hypothesize that this molecule could act as a reversible inhibitor of STAT3 dimerization.

### 2.2. Chemistry

**Scheme 1:**
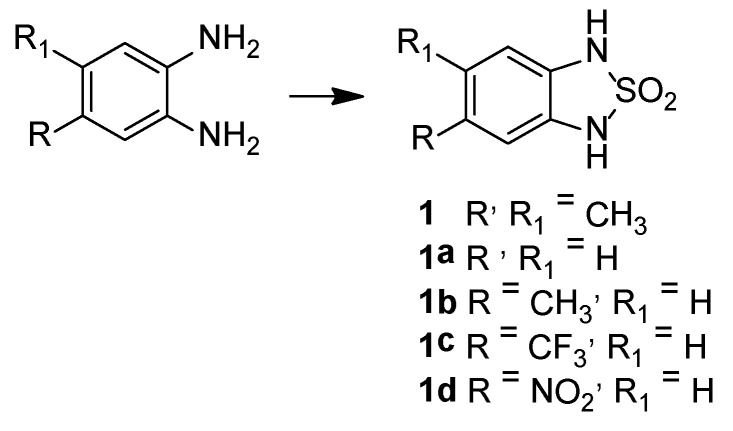
Reagents and conditions: NH_2_SO_2_NH_2_, diglyme, reflux, 40 min (10 min for compound **1**).

Compounds **1** and **1a**–**d** were synthesized from the corresponding commercially available *ortho*-diamines through a direct cyclization in the presence of sulfamide and diglyme (Scheme 1) [21].

**Figure 2:**
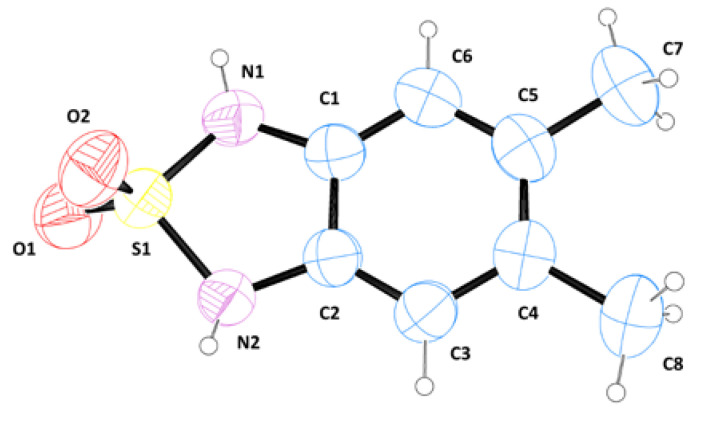
ORTEP [23] view of compound **1** and the relative arbitrary atom-numbering scheme (thermal ellipsoids at 50% probability).

Compound **1** showed acidic properties (pKa = 6.88 ± 0.03) [22] and was fully characterized by X-ray diffraction. Its crystal structure is shown in Figure 2 as an ORTEP [23] drawing (Figure 2).

**Scheme 2:**
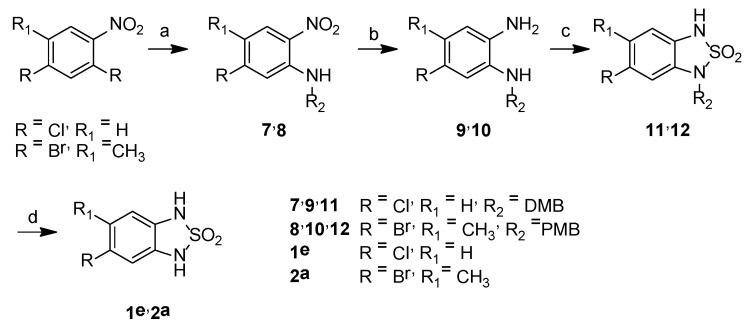
Reagents and conditions: (**a**) 2,4-dimethoxybenzylamine (DMB-NH_2_) (for **7**) or 4-methoxybenzylamine (PMB-NH_2_) (for **8**), isopropanol, reflux, 12 h; (**b**) Zn, NH_4_Cl, MeOH/H_2_O (9:1), reflux, 3 h; (**c**) NH_2_SO_2_NH_2_, dry diglyme, 160 °C, 1.5 h; and (**d**) trifluoroacetic acid (TFA), DCM, 50 °C, 1.5 h.

Products **1e** and **2a** were obtained starting from 2,4-dichloro-1-nitrobenzene and 1,5-dibromo-2-methyl-4-nitrobenzene, respectively. They were reacted with 2,4-dimethoxybenzylamine (DMB-NH_2_) or 4-methoxybenzylamine (PMB-NH_2_) to give intermediates **7** and **8**, respectively, which were converted to the corresponding amines **9** and **10** through reduction with zinc and ammonium chloride. By treatment with sulfamide, **9** and **10** underwent a cyclization reaction, thus affording intermediates **11** and **12**. These benzothiadiazole derivatives were deprotected with trifluoroacetic acid (TFA) to yield the final products **1e** and **2a**, respectively (Scheme 2).

**Scheme 3:**
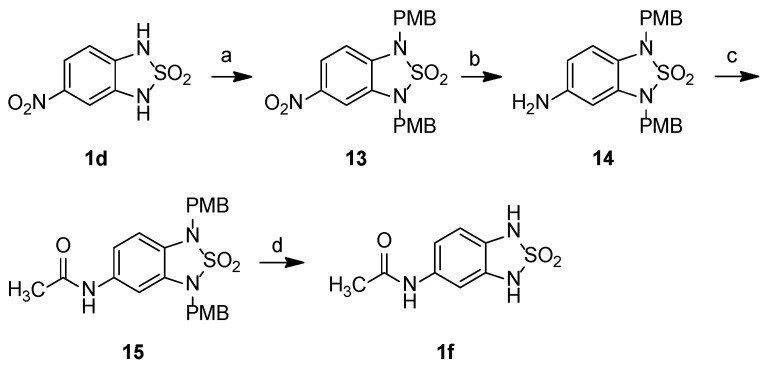
Reagents and conditions: (**a**) PMB-Br, dry K_2_CO_3_, dry DMF, 80 °C, 2 h; (**b**) Zn, NH_4_Cl, MeOH/H_2_O (9:1), reflux, 3 h; (**c**) (CH_3_COO)_2_O, (CH_3_)_2_CO, r.t., 3 h; and (**d**) TFA, DCM, r.t., 8 h.

Product **1f** was obtained starting from compound **1d**, which was protected by treatment with PMB-Br and potassium carbonate to afford intermediate **13**; its reduction with zinc and ammonium chloride gave the amine **14**. The latter, through a reaction with acetic anhydride, afforded intermediate **15**, which was deprotected with TFA to give product **1f** (Scheme 3).

**Scheme 4:**
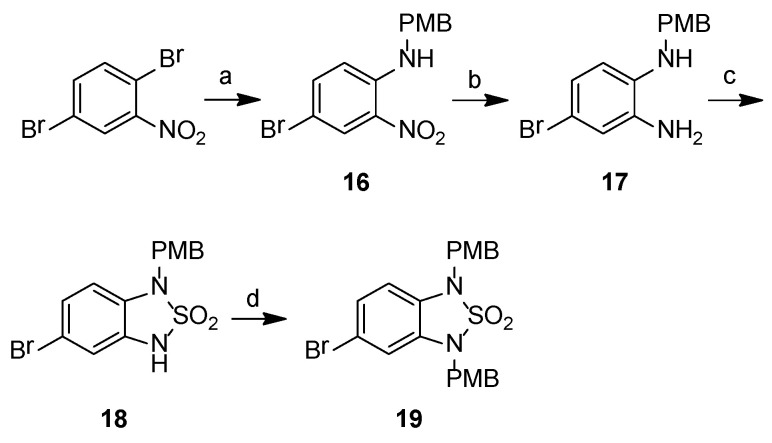
Reagents and conditions: (**a**) PMB-NH_2_, reflux, 8 h; (**b**) Zn, NH_4_Cl, MeOH/H_2_O (9:1), reflux, 4 h; (**c**) NH_2_SO_2_NH_2_, dry diglyme, 160 °C, 3 h; and (**d**) PMB-Br, dry K_2_CO_3_, dry DMF, 80 °C, 2 h.

Intermediate **19**, the starting reagent for the synthesis of products **1g**–**i**, was obtained as reported in Scheme 4: the reaction of 2,5-dibromo-nitrobenzene with PMB-NH_2_ led to intermediate **16**, which was reduced to give the amine **17** in the presence of zinc and ammonium chloride. By treatment with sulfamide, **17** was cyclized, thus affording the benzothiadiazole derivative **18**, which was subsequently protected with PMB-Br to yield compound **19** (Scheme 4).

**Scheme 5:**
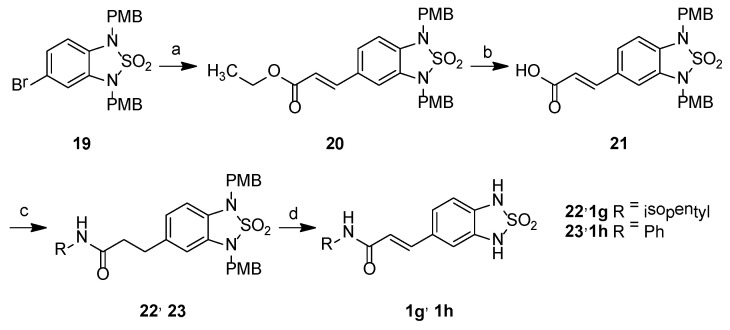
Reagents and conditions: (**a**) ethyl acrylate, P(*o*-tol)_3_, Pd_2_(dba)_3_, TEA, dry DMF, 80 °C, 20 h; (**b**) 3 M NaOH, MeOH, 80 °C, 3 h; (**c**) isopentylamine, HATU, DIPEA, DCM, r.t., 20 h (for **22**) or aniline, TBTU, *N*-methylmorpholine, DCM, r.t., 24 h (for **23**); and (**d**) H_2_ (3 atm), 10% Pd/C, EtOH, r.t., 48 h.

Afterwards, intermediate **19** was reacted with ethyl acrylate in the presence of tri-*o*-tolylphosphine and tris(dibenzylideneacetone)dipalladium(0) to afford the ester **20**. The latter was hydrolyzed under basic conditions to the corresponding acid **21**, which was converted to the amides **22** and **23** using isopentylamine, HATU, and *N*,*N*-diisopropylethylamine or aniline, TBTU, and *N*-methylmorpholine, respectively. By catalytic hydrogenation with 10% Pd/C, they were reduced and deprotected to give products **1g** and **1h** (Scheme 5).

**Scheme 6:**
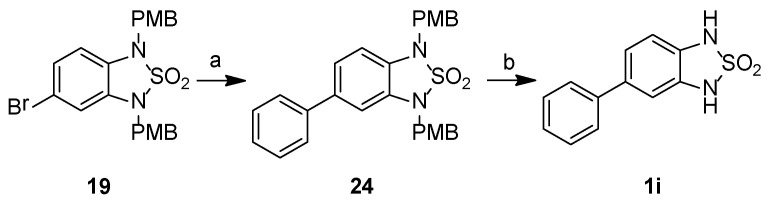
Reagents and conditions: (**a**) PhB(OH)_2_, Pd(PPh_3_)_4_, Na_2_CO_3_, 1,4-dioxane, reflux, 20 h; and (**b**) TFA, DCM, r.t., 3 h.

Moreover, compound **19** was arylated through a Suzuki reaction with phenylboronic acid in the presence of tetrakis(triphenylphosphine)palladium(0) to give intermediate **24**, which was then deprotected by treatment with TFA, thus affording product **1i** (Scheme 6).

**Scheme 7:**
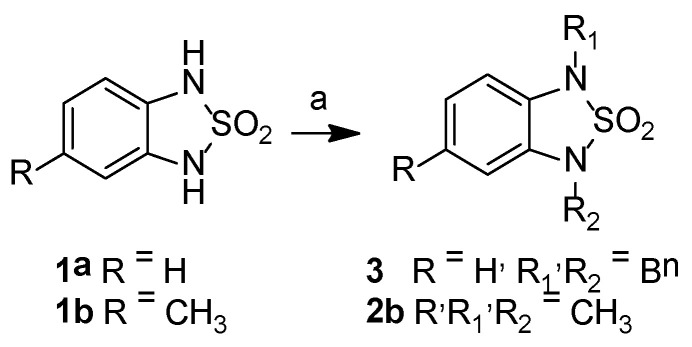
Reagents and conditions: CH_3_I (for **2b**) or BnBr (for **3**), dry K_2_CO_3_, and dry DMF, 80 °C, 2 h.

Products **2b** and **3** were obtained from compounds **1b** and **1a**, respectively, through alkylation with iodomethane or benzyl bromide in the presence of potassium carbonate (Scheme 7).

**Scheme 8:**
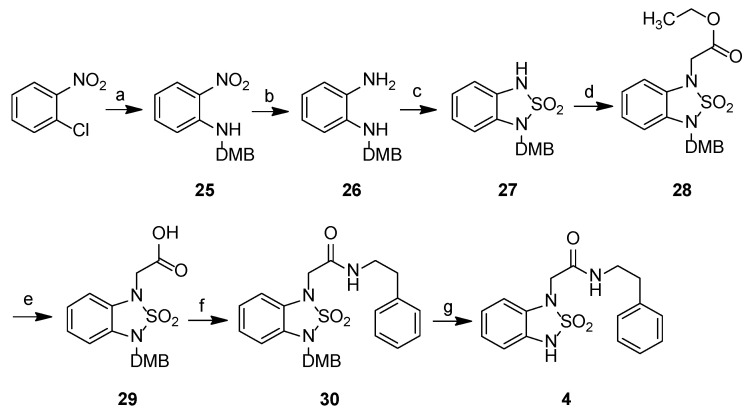
Reagents and conditions: (**a**) DMB-NH_2_, EtOH, reflux, 12 h; (**b**) Zn, NH_4_Cl, MeOH/H_2_O (9:1), reflux; (**c**) NH_2_SO_2_NH_2_, dry diglyme, 160 °C, 1.5 h; (**d**) BrCH_2_COOCH_2_CH_3_, dry K_2_CO_3_, dry DMF, 80 °C, 2 h; (**e**) K_2_CO_3_, MeOH, 50 °C, 2 h; (**f**) Ph(CH_2_)_2_NH_2_, HATU, DIPEA, DCM, r.t., 12 h; and (**g**) TFA, DCM, 45 °C, 45 min.

Product **4** was prepared through a reaction of 1-chloro-2-nitrobenzene with DMB-NH_2_ to obtain intermediate **25**, which was reduced using zinc and ammonium chloride to give the amine **26**. Its cyclization with sulfamide led to intermediate **27**, which was firstly esterified with ethyl bromoacetate to give intermediate **28** and then hydrolyzed under basic conditions to the corresponding acid **29**. This intermediate was condensed with 2-phenylethylamine to afford the amide **30**, which was deprotected with TFA, thus leading to the product **4** (Scheme 8).

**Scheme 9:**
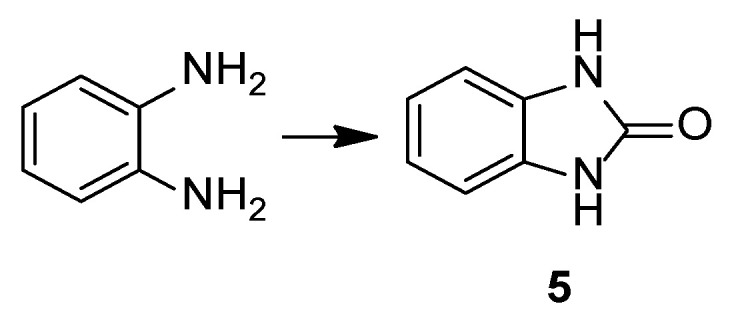
Reagents and conditions: CDI, dry THF, reflux, 12 h.

Product **5** was achieved through the cyclization of benzene-1,2-diamine in the presence of carbonyldiimidazole (CDI) (Scheme 9).

Finally, compound **6** was purchased from commercial sources.

### 2.3. Biological Evaluation

AlphaScreen is an in vitro competitive binding test used to identify compounds that are able to inhibit the binding of proteins containing an SH2 domain to their corresponding phosphopeptides (5-carboxyfluorescein (FITC)-GpYLPQTV for STAT3 and FITC-GpYDKPHVL for STAT1) (Table 2). Compounds characterized by an interesting STAT3 affinity were further investigated for their selectivity versus STAT1, which shows a high degree of sequence similarity to STAT3 but an opposite physiological role [24,25], and Grb2, as a model for other SH2 domain-containing proteins.

The significant activity of compound **1**, revealed at 30 µM (Table 2), prompted us to test this compound at different concentrations. Therefore, we calculated its IC_50_ values against STAT3 and STAT1 to be 15.8 ± 0.6 and ˃50 µM, respectively, while the inhibition versus Grb2 was 23% (at a 30 µM concentration).

To identify a trend in terms of structure–activity relationship, we firstly explored the role of the substituents on the benzene ring (**1a**–**i**). In this respect, we found that lipophilicity does not play an important role in modulating STAT3 inhibitory activity. Bulky lipophilic substituents at position 5 (derivatives **1g** and **1h**) induced a significant drop in the inhibition properties (Table 2). Among these derivatives, only **1d**, bearing a NO_2_ group at position 5, exhibited a noticeable activity against STAT3. For this compound, we calculated the IC_50_ values against STAT3 and STAT1, which were 25.1 ± 2.6 µM and 15.9 ± 2.0 µM, respectively, revealing no selectivity for STAT3 inhibition. Then, we introduced different groups on the nitrogen atoms of the thiadiazole ring (**2a**,**b**, **3**, and **4**), in order to explore asymmetrical substitutions: The presence of the N-substituent did not improve activity. The enhanced bulkiness and hydrophobicity of these substituents compared to compound **1** did not lead to a better interaction.

Finally, we investigated the importance of the thiadiazole ring (**5** and **6**) through the exploration of the function of the SO_2_ group and of the nitrogen atoms: The results of the AlphaScreen-based assay indicated that these moieties play a key role in the interaction with the STAT3-SH2 domain.

Overall, our data seem to indicate that this class of benzothiadiazole derivatives is characterized by an extremely “tight” SAR: Most of the modifications applied to the scaffold of compound **1** resulted in a considerable loss of activity, with the sole exception of **1d**, which retained a partial inhibitory effect.

Since from the literature it is known that some benzothiadiazoles could covalently bind to proteins [26], an in silico prediction to evaluate whether the proposed derivatives were pan-assay interference compounds (PAINS) was performed by submitting their structures to five on-line services (FAF-Drugs4 [27], PAINS remove [28], SmartsFilter, (http://pasilla.health.unm.edu/tomcat/biocomp/smartsfilter), SwissADME [29], and Zinc Patterns (http://zinc15.docking.org/patterns/home/)). Three of them (SmartsFilter, SwissADME, and Zinc Patterns) identified compounds **1**, **1a**–**i**, and **2a** as potential PAINS (see Table S6).

This result was experimentally confuted, at least for compound **1**, because it was found to act as a selective STAT3 inhibitor with an IC_50_ of 15.8 ± 0.6 µM, which was significantly lower than the IC_50_ shown versus STAT1 (˃50 µM). This selectivity was remarkable considering the high similarity and identity of both STAT1 and STAT3, for the SH2 domain in particular (73.5% sequence similarity and 56.1% sequence identity, calculated by EMBOSS Needle [30]).

**Figure 3:**
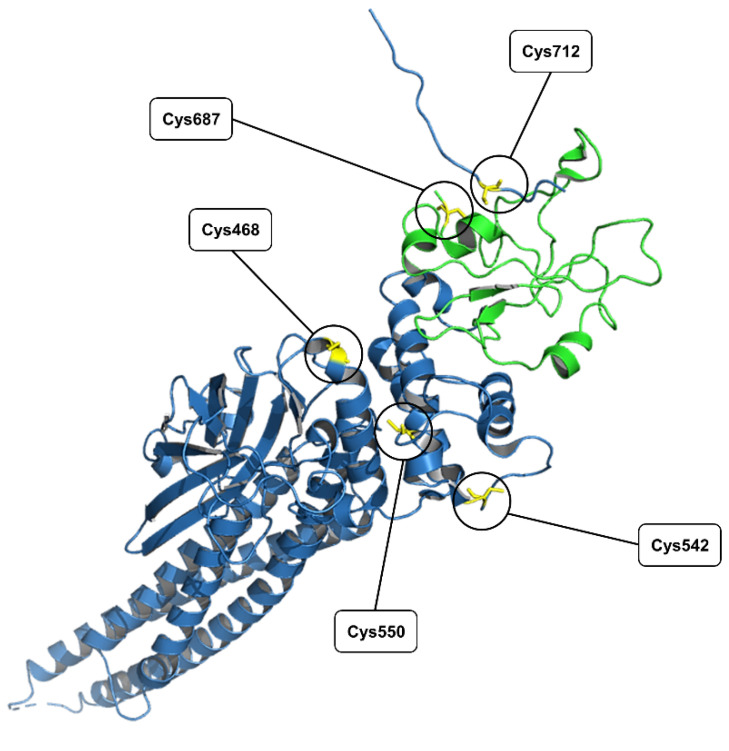
Ribbon diagram of STAT3 (blue), with the SH2 domain in green and the mutated cysteine residues in yellow (Protein Data Bank (PDB) ID 1BG1).

Furthermore, to gain insight into the activity of compound **1**, its interaction with the cysteine residues located in the vicinities of the SH2 domain (Figure 3) was investigated and evaluated by an AlphaScreen-based assay using different mutants (Table 3).

Compound **1** was inactivated by the addition of an excess amount of cysteine into the assay medium (see Table S7). Moreover, the point mutations of Cys468, Cys542, Cys550, Cys687, or Cys712 in STAT3 conferred resistance to the compound (see Table S8), suggesting that each cysteine residue partially contributes to the inhibition mediated by the molecule.

Though STAT3 contains twelve cysteine residues, the assays were performed only on clones bearing mutations on the five cysteines located near the STAT3-SH2 domain. Therefore, we cannot exclude the possibility that other cysteines may be involved in the interaction with compound **1**.

Considering the affinity of compound **1** for STAT3 wild-type, we decided to explore its mechanism of interaction with cysteines by means of analytical studies.

In order to analyze the binding mode of compound **1** to the pockets containing the mutated cysteine residues, a molecular docking study was carried out. In particular, we applied the same protocol used for the virtual screening, but, in this case, the grid maps were calculated by selecting the atoms included in a sphere of 12 Å radius that were centered on each cysteine residue. The so-obtained complexes were optimized with the same minimization protocol employed for the preparation of the STAT3 crystal structure (30,000 steps of conjugate gradients minimization). For a better analysis of the poses, the distances between the cysteine sulfur and the two hypothetical reactive centers (the methyl in 5 and the aromatic carbon in 4) of compound **1** were evaluated. The results in Table S9 show that only the complex with Cys712 seems to have the suitable prerequisites in terms of distances to the reactive centers (4.70 and 4.95 Å for CH_3_–S and CH–S, respectively; see Figure S72) and docking energy (−5.93 Kcal/mol), even if the latter parameter must be taken into account with caution because it was calculated by molecular mechanics. The complex with Cys550 also seemed to have the right distances to react with compound **1** (3.64 and 5.13 Å for CH_3_–S and CH–S, respectively); however, the interaction energy was found to be significantly worse than that of the complex with Cys712 (−3.52 versus −5.93 Kcal/mol). Unfortunately, this kind of behavior could not be confirmed by the experimental data; this was probably due to the intrinsic problem afflicting all docking calculations that could only evaluate the physicochemical and steric complementarity of two interacting partners and not the reactivity of the atoms.

### 2.4. Analytical Studies

#### 2.4.1. MS, UV, and LC Studies

**Figure 4:**
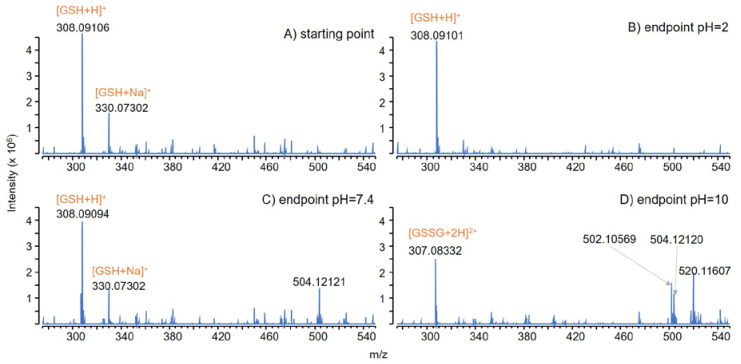
MS spectra of glutathione (GSH) incubated with compound **1** as recorded at incubation starting point (**A**), at incubation endpoint in a pH 2 buffer (**B**), at incubation endpoint in a pH 7.4 buffer (**C**), and at incubation endpoint in a pH 10 buffer (**D**).

MS studies revealed that compound **1** reacts with glutathione (GSH, used as a model for cysteine-containing peptides) to generate stable covalent adducts. Since no adducts were detectable at the incubation starting point, such reactions did not take place during sample transfer into the MS analyzer. Similar approaches have already been used to identify reactive intermediates by MS studies [31]. The reaction was pH-dependent and required a neutral or basic pH to take place, whereas no reactivity was observed at an acidic pH (Figure 4).

The absence of reactivity at the acidic pH can be explained on one hand by the pH-dependent nucleophilicity of GSH [32] and on the other hand by the deprotonation of compound **1** occurring at a neutral/basic pH (pKa = 6.88 ± 0.03) [22], which can lead to structure rearrangements that possibly contribute to an increased reactivity towards GSH. However, the methyl groups seem fundamental for the covalent reaction, since no adducts were detectable upon incubation of GSH with compound **1d**, which was the second most active compound according to the results reported in Table 2.

At a neutral pH (Figure 4C), a single adduct was detectable at the reaction endpoint (504.12121 *m*/*z*), whereas at a basic pH (Figure 4D), three signals at 502.10569, 504.12120, and 520.11607 *m*/*z* were detected along with the complete oxidation of GSH to its dimer GSSG (i.e., the conversion of the singly charged GSH ion at 308.09106 *m*/*z* to the doubly charged GSSG ion at 307.08332 *m*/*z*).

The signals at 502.10569, 504.12120, and 520.11607 *m*/*z* were all within 1 ppm from the theoretical *m*/*z* values expected for singly charged ions of molecules C_18_H_25_N_5_O_8_S_2_, C_18_H_23_N_5_O_8_S_2_, and C_18_H_25_N_5_O_9_S_2_, respectively.

**Figure 5:**
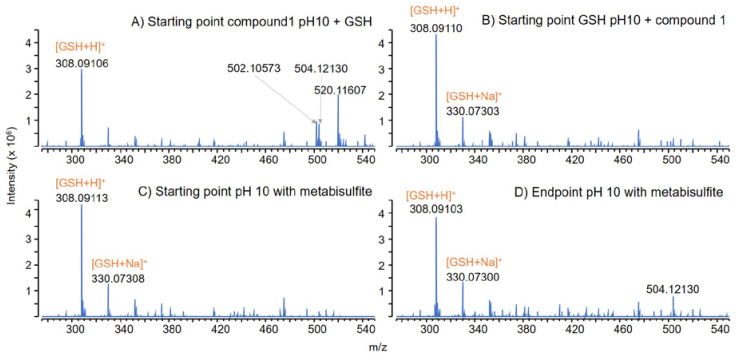
MS spectra at the reaction starting point for a 1:1 GSH:compound **1** mixture incubated at pH 10 (spectra A and B). Samples were prepared by adding compound **1** (spectrum **A**) or GSH (spectrum **B**) as the first reagent. MS spectra at the incubation starting point (spectrum **C**) and endpoint (spectrum **D**) for a 1:1 GSH:compound **1** mixture incubated at pH 10 with Na_2_S_2_O_5_ in the buffer.

Interestingly, at pH 10, the adducts were also detected at the incubation starting point if compound **1** was added before GSH into the buffer (Figure 5A), but not if GSH (Figure 5B) or Na_2_S_2_O_5_ (Figure 5C) were added into the buffer before compound **1**.

Since GSH is an antioxidant, we speculated on the involvement of oxidation steps for the formation of adducts. To address such a hypothesis, the experiments were repeated in a buffer containing Na_2_S_2_O_5_, which had similar effect of GSH at the incubation starting point (Figure 5C) and prevented the formation of adducts at 502.10569 and 520.11607 *m*/*z* at the incubation endpoint (Figure 5D), suggesting that such adducts are generated only in oxidizing conditions.

On the contrary, the adduct at 504.12130 *m*/*z* was detectable at the incubation endpoint, despite Na_2_S_2_O_5_ addition (Figure 5D).

**Figure 6:**
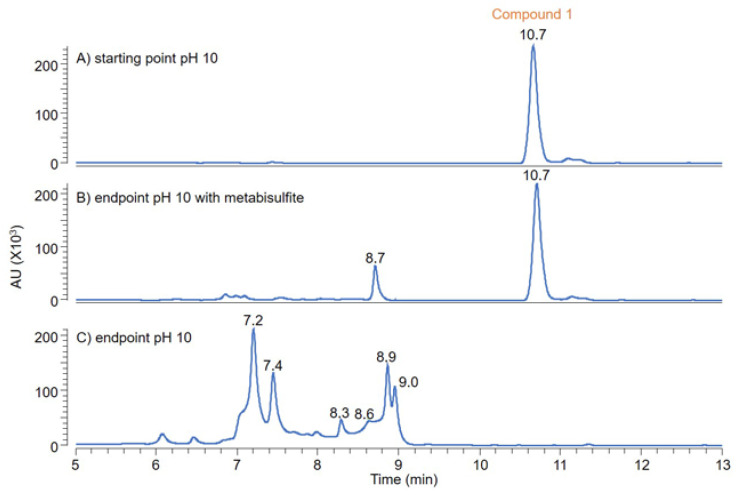
Chromatograms of GSH incubated with compound **1** as recorded at incubation starting point (**A**), at incubation endpoint in a pH 10 buffer containing Na_2_S_2_O_5_ (**B**), and at incubation endpoint in a Na_2_S_2_O_5_-free pH 10 buffer (**C**).

Consistently with MS data, LC-UV experiments detected a single adduct when compound **1** was incubated with GSH and Na_2_S_2_O_5_ (i.e., chromatographic peak at RT = 8.7 min, Figure 6B), whereas multiple peaks were generated if no antioxidants were added into the buffer (Figure 6C).

Na_2_S_2_O_5_ also prevented the complete consumption of compound **1** (Figure 5B), thus confirming that oxidizing conditions trigger a faster reaction rate.

Moreover, despite MS experiments detecting an adduct at 504.12125 ± 0.00006 *m*/*z*, both with and without Na_2_S_2_O_5_, the corresponding chromatograms had no common peaks. This means that oxidizing and non-oxidizing conditions lead to the formation of adducts with different structures but the same elemental composition.

**Figure 7:**
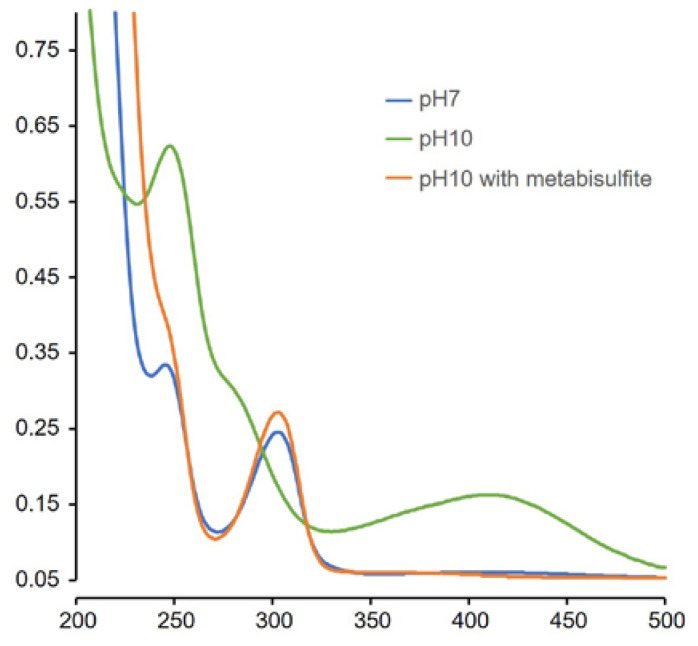
UV spectra at the endpoint of the reaction between GSH and compound **1**. Reaction was buffered at pH 7.4 (blue line), pH 10 (green line), or pH 10 with Na_2_S_2_O_5_ (orange line).

This hypothesis was also supported by the UV spectra collected for the corresponding reaction batches. As reported in Figure 6, the UV spectra at the reaction endpoint look alike for reactions buffered at pH 7.4 or pH 10 with Na_2_S_2_O_5_, with no shifts compared to the starting point (data not shown). On the contrary, the incubation at pH 10 without Na_2_S_2_O_5_ was only found to have a similar shape at the starting point, while a significant shift was observed at the reaction endpoint (Figure 7).

**Figure 8:**
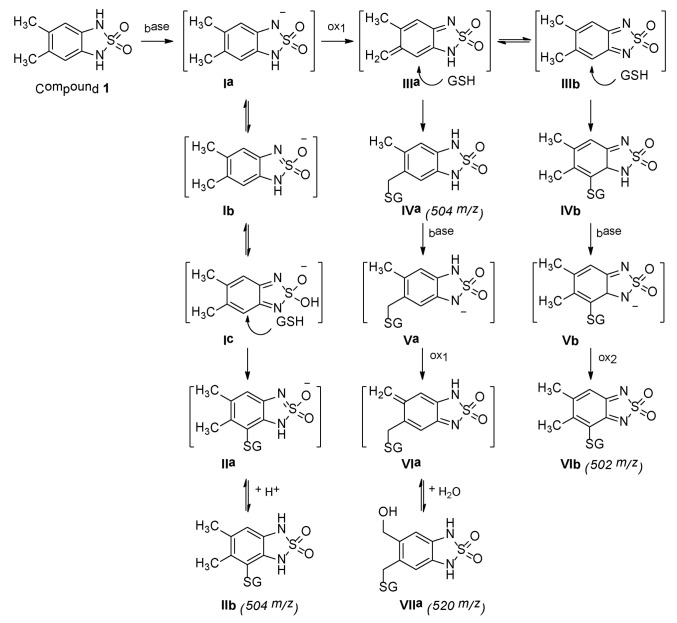
Proposed reaction mechanisms for the generation of compound **1**—glutathione adducts. Group SG indicates covalently attached glutathione via thioether linkage.

The reaction mechanism in Figure 8 accounts for all the observations described above.

Specifically, the overall mechanism requires a neutral-to-basic pH for compound **1** deprotonation to generate adducts. All included oxidation steps are dependent on the basic pH activation of compound **1**, like with catechol [33], and provide the generation of reactive intermediates similar to quinones and quinone-methides that are able to covalently react with GSH [34,35,36]. The final products **VIb** and **VIIa** are generated only after the oxidative rearrangement of compound **1** and have molecular formulas in agreement with the MS signals at 502 and 520 *m*/*z*, found only in reaction batches without antioxidants. The products **IVa** and **IIb** were found to have the same molecular formula, which was in agreement with the signal at 504 *m*/*z* that was found in reaction batches with or without antioxidants. However, consistently with LC experiments, the products **IVa** and **IIb** were found to have different structures and formation mechanisms: **IVa** was found to be generated after compound **1** oxidation, while the formation of **IIb** was not found to require the oxidation of compound **1**. Interestingly, such mechanism is not specific for the class of compounds since it was only observed for compound **1**. The removal of the methyl groups led to compounds that were unable to covalently react with glutathione, despite some of them still being capable of inhibiting STAT3 activity (e.g., compound **1d**; see Table 2).

#### 2.4.2. ^1^H-NMR Analysis

With the aim of confirming the stated hypotheses, NMR studies were performed in the experimental conditions previously considered for the MS analysis in the absence or in the presence of GSH. Actually, few NMR studies concerning covalent interactions between GSH/STAT proteins and small molecules have been reported. In one case [37], the authors exploited the presence of fluorine atoms on the studied compounds and used ^19^F-NMR spectroscopy, which has the advantage of simple spectra with a wide spread of resonances.

In our case, we used ^1^H-NMR spectroscopy even though it is known that GSH, like other small peptides, presents a great conformational variability, also depending on the pH, that influences its ionization state [38]; consequently, its ^1^H-NMR spectra could appear quite complex.

Therefore, to study the interaction of compound **1** with the model peptide GSH, we decided to perform a comparison, at different times, of the spectra of compound **1** and of GSH alone with the spectrum of a mixture of them at pH 10 and 37 °C. These conditions were chosen because they proved to be optimal for the interaction between the two compounds in the MS investigations. Moreover, in order to make the NMR study easier, we decided to add Na_2_S_2_O_5_ to the mixture to reduce the number of adducts that could potentially derive from the reactions between compound **1** and GSH. The use of this reducing agent made the MS spectra less complex, mainly showing a 504 [M + H]^+^ ion (Figure 5D) corresponding to few hypothetical structures (**IIb** and **IVa**; Figure 8); in the same conditions, the LC chromatogram revealed essentially one peak at 8.7 min, even if this analysis showed only a reduced transformation of the starting compound **1** at the incubation endpoint (Figure 6B). Moreover, the addition of Na_2_S_2_O_5_ should have prevented the presence of interfering NMR resonances due to the formation of oxidized GSSG.

Though this study did not demonstrate the formation of the expected compounds (see discussion in Supplementary Materials and Figures S72–S74), we believe that the NMR outcomes cannot disprove the results obtained by MS spectroscopy.

## 3. Experimental Section

### 3.1. Computational Methods

The structural coordinates of STAT3, co-crystallized with a DNA fragment, were downloaded from the Protein Data Bank (PDB ID 1BG1) [19]. The protein was dimerized by applying the transformation matrix, as shown in the PDB file, and the model was completed by the addition of the hydrogens in two steps: (1) to STAT3, applying the algorithm for proteins, and (2) to DNA, applying the algorithm for nucleic acids. In both cases, we used the features included in the VEGA ZZ package [39]. Atom charges (Gasteiger–Marsili method) [40] and potentials (CHARMM 22 for proteins and nucleic acids) [41,42] were assigned to the obtained structure. Finally, the model was optimized by a conjugate gradient minimization (30,000 steps) to reduce the high-energy steric interactions. In order to preserve the experimental data, atom constraints were applied to protein and DNA backbones. This step was carried out by NAMD 2.13 [43], which was integrated in the VEGA ZZ graphic environment. To perform virtual screening calculations, the following databases were chosen: AKos (AKos Consulting & Solutions GmbH, Steinen, Germany; 544,391 molecules), ChemPDB (4009), ChemBank (Broad Institute, Cambridge, MA, USA; 2344), KEGG (Kyoto Encyclopedia of Genes and Genomes, Kanehisa Laboratories, Kyoto, Japan; 10005), NCI’s Anti-HIV (National Cancer Institute, National Institute of Health, USA; 42689), and NCI (National Cancer Institute, National Institute of Health, USA; 15237). These databases are available for free at the mirror site of Ligand.info (http://biophysics.med.jhmi.edu/~yliu120/dockingdatabase.html; the original database site is no longer available) [44], which includes collections of compounds for virtual screening. These structures are already converted to 3D and fully optimized by molecular mechanics. Nevertheless, all molecules were pre-processed setting their ionization state at a physiological pH, and the resulting structures were optimized and refined by PM7 semi-empirical method, as implemented in MOPAC 2016. To perform this calculation, the WarpEngine technology implemented in VEGA ZZ [45] was used, reducing the computational time to few tens of minutes for the whole dataset through the distribution of the MOPAC calculation on a network of PCs. In more detail: Three blade servers equipped with two CPUs, each of Intel Xeon E5 class, were used for a total of 52 physical cores and 104 threads. The virtual screening was carried out by the GriDock software, which was especially designed to run on high performance computing systems (HPC) and distributed computer resources (GRID) as front-end to the well-known AutoDock 4 package [20]. Specifically, GriDock is able to extract each molecule from a database and perform a docking calculation through AutoDock 4. Though it is currently possible to perform screenings with faster and more efficient programs, we preferred to spend more time on the calculation because we had already obtained good results with AutoDock in previous studies on STAT3. Before running GriDock, a single STAT3 monomer was considered, and the grid maps, required to evaluate docking scores, were calculated by selecting the atoms included in a sphere of 12 Å radius centered on the phosphorylated Tyr-705 (PTR-705 in the PDB file). This pocket represented a possible hot spot of interaction with STAT3 for ligands, since it is known to play a pivotal role in STAT3 dimerization and activation. This process was carried out by AutoGrid 4 interfaced to VEGA ZZ. The compounds of the considered databases were docked by GriDock/AutoDock using the genetic-algorithm search and generating ten possible solutions.

### 3.2. Chemistry

#### 3.2.1. Materials and Methods

All starting materials, chemicals, and solvents were purchased from commercial suppliers (Sigma-Aldrich-Merck, St. Louis, MI, USA; FluoroChem, Hadfield, UK) and used as received. Anhydrous solvents were utilized without further drying. Aluminum-backed silica gel 60 plates (0.2 mm; Merck, Darmstadt, Germany) were used for analytical thin-layer chromatography (TLC) to follow the course of the reactions. Silica gel 60 (40–63 μm; Merck) was used for the purification of intermediates and final compounds through flash column chromatography. Melting points were determined in open capillary tubes with a Stuart SMP30 Melting Point Apparatus (Cole-Parmer Stuart, Stone, UK) and are uncorrected. ^1^H and ^13^C-NMR spectra were acquired at ambient temperature with a Varian Oxford 300 MHz instrument (Varian, Palo Alto, CA, USA),operating at 300 MHz for ^1^H and 75 MHz for ^13^C. Chemical shifts are expressed in ppm (δ) from tetramethylsilane (TMS) resonance in the indicated solvent (TMS: δ = 0.0 ppm), while *J*-couplings are given in hertz. All the spectra are reported in the Supplementary Materials. The purity of the tested compounds was assessed by means of elemental analysis using a EuroVector EA 3000 CHNS-O analyzer (EuroVector, Pavia, Italy). All experimental values are within ±0.40% of the theoretical predictions, indicating a ≥95% purity.

#### 3.2.2. Synthetic Procedures

##### 5,6-Dimethyl-1*H*,3*H*-2,1,3-benzothiadiazole-2,2-dioxide, **1**

Procedure A: To a solution of 4,5-dimethyl-1,2-phenylenediamine (100 mg, 0.73 mmol) in dry diglyme (0.75 mL) under reflux, sulfamide (86 mg, 0.89 mmol) dissolved in dry diglyme (0.73 mL) was slowly added. The mixture was stirred for 10 min at 160 °C. After cooling, the dark brown mixture was diluted with ice-H_2_O and extracted with EtOAc (3 × 5 mL). The organic phase was washed repeatedly with cold H_2_O to remove the diglyme. Then, it was treated with Na_2_SO_4_, filtered, and evaporated under reduced pressure. The crude product was purified by flash column chromatography (cyclohexane/EtOAc 8:2) to obtain **1** as a brownish solid. Yield: 50%. TLC (cyclohexane/EtOAc 8:2): R_f_: 0.36. Mp: 148 °C. **^1^**H-NMR (300 MHz, acetone-*d*_6_): δ 9.25 (s, 2H, NH), 6.72 (s, 2H, ArH), 2.18 (s, 6H, CH_3_) ppm. ^13^C-NMR (75 MHz, D_2_O): δ 134.6, 127.0, 111.1, 18.4 ppm. Anal. calcd (%) for C_8_H_10_N_2_O_2_S: C, 48.47; H, 5.08; N, 14.13; S, 16.17; found: C, 48.21; H, 5.03; N, 14.67; S, 16.34.

##### 1*H*,3*H*-2,1,3-Benzothiadiazole-2,2-dioxide, **1a**

Benzene-1,2-diamine was reacted for 1.5 h using Procedure A; the purification was performed by flash column chromatography (cyclohexane/EtOAc 6:4) to afford the compound as a dark yellow solid. Yield: 88%. TLC (cyclohexane/EtOAc 6:4): R_f_: 0.23. Mp: 171–173 °C. ^1^H-NMR (300 MHz, CD_3_OD): δ 6.93–6.91 (m, 2H, ArH), 6.84–6.81 (m, 2H, ArH) ppm. ^13^C-NMR (75 MHz, CD_3_OD): δ 129.9, 121.5, 110.2 ppm. Anal. calcd (%) for C_6_H_6_N_2_O_2_S: C, 42.34; H, 3.55; N, 16.46; S, 18.84; found: C, 41.98; H, 3.59; N, 16.35; S, 18.75.

##### 5-Methyl-1*H*,3*H*-2,1,3-benzothiadiazole-2,2-dioxide, **1b**

4-methylbenzene-1,2-diamine was reacted for 1.5 h using Procedure A; the crude residue was purified by flash column chromatography (DCM/MeOH 98:2) to give a dark red solid. Yield: 55%. TLC (DCM/MeOH 9:1): R_f_: 0.54. Mp: 157 °C. ^1^H-NMR (300 MHz, CD_3_OD): δ 6.73–6.66 (m, 3H, ArH), 2.28 (s, 3H, CH_3_) ppm. ^13^C-NMR (75 MHz, CD_3_OD): δ 131.6, 130.1, 127.5, 121.8, 110.8, 110.2, 19.9 ppm. Anal. calcd (%) for C_7_H_8_N_2_O_2_S: C, 45.64; H, 4.38; N, 15.21; S, 17.41; found: C, 45.11; H, 4.51; N, 14.96; S, 17.25.

##### 5-(Trifluoromethyl)-1*H*,3*H*-2,1,3-benzothiadiazole-2,2-dioxide, **1c**

4-(trifluoromethyl)benzene-1,2-diamine was reacted for 1.5 h using Procedure A; the purification was performed by flash column chromatography (DCM/MeOH 9:1) to give a white solid. Yield: 66%. TLC (DCM/MeOH 9:1): R_f_: 0.19. Mp: 143 °C. ^1^H-NMR (300 MHz, CD_3_OD): δ 7.23 (dd, *J* = 8.2, 2.0 Hz, 1H, ArH), 7.06 (d, *J* = 2.0 Hz, 1H, ArH), 6.94 (d, *J* = 8.2 Hz, 1H, ArH) ppm. ^13^C-NMR (75 MHz, CD_3_OD): δ 132.61, 129.65, 123.38, 123.06, 123.00, 118.66 (q, *J* = 4.2 Hz), 109.34, 106.41 (q, *J* = 4.2 Hz) ppm. Anal. calcd (%) for C_7_H_5_F_3_N_2_O_2_S: C, 35.30; H, 2.12; N, 11.76; O, 13.43; S, 13.46; found: C, 35.61; H, 2.17; N, 11.94; S, 13.78.

##### 5-Nitro-1*H*,3*H*-2,1,3-benzothiadiazole-2,2-dioxide, **1d**

4-nitrobenzene-1,2-diamine was reacted for 1.5 h using Procedure A; the residue was purified by flash column chromatography (DCM/MeOH 9:1) to give a red solid. Yield: 50%. TLC (DCM/MeOH 9:1): R_f_: 0.15. Mp: 190 °C. ^1^H-NMR (300 MHz, CD_3_OD): δ 7.66 (dd, *J* = 8.7, 2.5 Hz, 1H, ArH), 7.38 (d, *J* = 2.5 Hz, 1H, ArH), 6.53 (d, *J* = 8.7 Hz, 1H, ArH) ppm. ^13^C-NMR (75 MHz, CD_3_OD): δ 144.2, 140.1, 133.4, 119.6, 108.9, 104.3 ppm. Anal. calcd (%) for C_6_H_5_N_3_O_4_S: C, 33.49; H, 2.34; N, 19.53; O, 29.74; S, 14.90; found: C, 33.74; H, 2.38; N, 19.41; S, 14.51.

##### 5-Chloro-*N*-[(2,4-dimethoxyphenyl)methyl]-2-nitroaniline, **7**

Procedure B: A mixture of 2,4-dichloro-1-nitrobenzene (191 mg, 1 mmol), 2,4-dimethoxybenzylamine (334 mg, 2 mmol), and isopropanol (2 mL) was heated at reflux for 16 h. After cooling, the reaction was diluted with H_2_O (4 mL) and extracted with EtOAc (2 × 4 mL). The collected organic layers were washed with 0.5 M HCl (1 × 5 mL) and then dried over anhydrous Na_2_SO_4_, filtered, and concentrated in vacuo. The crude residue was purified by recrystallization from EtOH to obtain intermediate **7** as an orange solid. Yield: 60%. TLC (hexane/EtOAc 85:15): R_f_: 0.36. Mp: 107–109 °C. ^1^H-NMR (300 MHz, CDCl_3_): δ 8.44 (br s, 1H, NH), 8.13 (d, *J* = 9.1 Hz, 1H, ArH), 7.18 (d, *J* = 8.2 Hz, 1H, ArH), 6.96 (d, *J* = 2.2 Hz, 1H, ArH), 6.60 (dd, *J* = 9.1, 2.2 Hz, 1H, ArH), 6.53 (d, *J* = 2.4, 1H, ArH), 6.49 (dd, *J* = 8.2, 2.4 Hz, 1H, ArH), 4.44 (d, *J* = 5.7 Hz, 2H, CH_2_), 3.89 (s, 3H, CH_3_), 3.84 (s, 3H, CH_3_) ppm. ^13^C-NMR (75 MHz, CDCl_3_): δ 160.9, 158.4, 145.8, 142.7, 130.7, 129.6, 128.2, 117.1, 115.7, 113.6, 104.3, 98.8, 55.42, 55.41, 42.2 ppm.

##### 5-Bromo-*N*-[(4-methoxyphenyl)methyl]-4-methyl-2-nitroaniline, **8**

1,5-dibromo-2-methyl-4-nitrobenzene and PMB-NH_2_ were reacted according to Procedure B to yield **8** as an orange solid. Yield: 95%. TLC (hexane/EtOAc 9:1): R_f_: 0.28. Mp: 115–116 °C. ^1^H-NMR (300 MHz, CD_3_OD): δ 8.37 (s, 1H, ArH), 8.27 (br s, 1H, NH), 7.28 (d, *J* = 8.7 Hz, 2H, ArH), 6.93 (d, *J* = 8.7 Hz, 2H, ArH), 6.75 (s, 1H, ArH), 4.46 (d, *J* = 5.4 Hz, 2H, CH_2_), 3.84 (s, 3H, CH_3_), 2.37 (s, 3H, CH_3_) ppm. ^13^C-NMR (75 MHz, CD_3_OD): δ 159.3, 147.1, 144.1, 132.0, 129.6, 128.9, 128.4, 115.4, 114.4, 110.1, 55.3, 46.7, 23.8 ppm.

##### 5-Chloro-*N*1-[(2,4-dimethoxyphenyl)methyl]benzene-1,2-diamine, **9**

Procedure C: Zn dust (350 mg, 5.36 mmol) and NH_4_Cl (46.6 mg, 0.87 mmol) were added in a single portion to a stirred suspension of intermediate **7** (216 mg, 0.67 mmol) in MeOH/H_2_O 9:1 (10 mL). The reaction was stirred at reflux for 3 h. After completion, the mixture was filtered through celite, and the filtrate was evaporated under vacuum. The residue was diluted with H_2_O (6 mL) and extracted with EtOAc (3 × 5 mL). The collected organic layers were dried over Na_2_SO_4_, filtered, and concentrated in vacuo. The crude residue was purified by flash column chromatography (hexane/EtOAc from 7:3 to 6:4) to obtain the desired compound as a brown solid. Yield: 71%. TLC (hexane/EtOAc 7:3): R_f_: 0.28. Mp: 135–138 °C. ^1^H-NMR (300 MHz, CDCl_3_): δ 7.21 (d, *J* = 8.2 Hz, 1H, ArH), 6.71 (d, *J* = 2.0 Hz, 1H, ArH), 6.65–6.62 (m, 2H, ArH), 6.52 (d, *J* = 2.4 Hz, 1H, ArH), 6.48 (dd, *J* = 8.2, 2.4 Hz, 1H, ArH), 4.22 (s, 2H, CH_2_), 3.86 (s, 3H, CH_3_), 3.83 (s, 3H, CH_3_), 3.48 (br s, 2H, NH_2_) ppm.

##### 5-Bromo-*N*1-[(4-methoxyphenyl)methyl]-4-methylbenzene-1,2-diamine, **10**

The compound was obtained from **8** following Procedure C. The crude residue was purified by flash column chromatography (hexane/EtOAc from 8:2 to 7:3) to afford a yellow solid. Yield: 71%. TLC (hexane/EtOAc 8:2): R_f_: 0.31. Mp: 147–149 °C. ^1^H-NMR (300 MHz, CDCl_3_): δ 7.32 (d, *J* = 8.8 Hz, 2H, ArH), 6.92 (d, *J* = 8.8 Hz, 2H, ArH), 6.91 (s, 1H, ArH), 6.56 (s, 1H, ArH), 4.22 (s, 2H, CH_2_), 3.84 (s, 3H, CH_3_), 3.30 (br s, 2H, NH_2_), 2.30 (s, 3H, CH_3_) ppm. ^13^C-NMR (75 MHz, CDCl_3_): δ 159.0, 137.3, 133.1, 131.1, 129.2, 129.1, 119.9, 114.1, 113.9, 112.4, 55.3, 48.1, 22.3 ppm.

##### 6-Chloro-1-[(2,4-dimethoxyphenyl)methyl]-1*H*,3*H*-2,1,3-benzothiadiazole-2,2-dioxide, **11**

Intermediate **9** was reacted for 1.5 h following Procedure A. The crude residue was purified by flash column chromatography (hexane/EtOAc 6:4) to afford a brown oil. Yield: 55%. TLC (hexane/EtOAc 6:4): R_f_: 0.21. ^1^H-NMR (300 MHz, CDCl_3_): δ 7.33 (d, *J* = 8.3 Hz, 1H, ArH), 6.86 (dd, *J* = 8.3, 2.0 Hz, 1H, ArH), 6.80–6.73 (m, 2H, ArH), 6.53–6.44 (m, 2H, ArH), 4.82 (s, 2H, CH_2_), 3.91 (s, 3H, CH_3_), 3.82 (s, 3H, CH_3_) ppm.

##### 6-Bromo-1-[(4-methoxyphenyl)methyl]-5-methyl-1*H*,3*H*-2,1,3-benzothiadiazole-2,2-dioxide, **12**

Intermediate **10** was reacted for 1.5 h following Procedure A. The purification was performed by flash column chromatography (hexane/EtOAc 6:4) to give the desired compound as a yellow oil. Yield: 56%. TLC (hexane/EtOAc 6:4): R_f_: 0.27. ^1^H-NMR (300 MHz, CDCl_3_): δ 7.39 (d, *J* = 8.8 Hz, 2H, ArH), 7.02 (s, 1H, ArH), 6.93 (d, *J* = 8.8 Hz, 2H, ArH), 6.42 (s, 1H, ArH), 4.79 (s, 2H, CH_2_), 3.83 (s, 3H, CH_3_), 2.25 (s, 3H, CH_3_) ppm. ^13^C-NMR (75 MHz, CDCl_3_): δ 159.6, 132.6, 130.7, 128.9, 126.0, 125.6, 115.8, 115.4, 114.4, 110.9, 55.3, 46.1, 23.0 ppm.

##### 5-Chloro-1*H*,3*H*-2,1,3-benzothiadiazole-2,2-dioxide, **1e**

Procedure D: Intermediate **11** (40 mg, 0.113 mmol) was dissolved in DCM (1 mL), and TFA (1 mL) was added dropwise. The reaction mixture was stirred for 45 min at 40 °C. After the evaporation of TFA under vacuum, MeOH was added and the white precipitate was filtered off. The filtrate was concentrated in vacuo and purified by flash column chromatography (hexane/EtOAc 6:4) to give product **1e** as a pale pink solid. Yield: 82%. TLC (hexane/EtOAc 6:4): R_f_: 0.48. Mp: 183 °C. ^1^H-NMR (300 MHz, CD_3_OD): δ 6.91 (dd, *J* = 8.4, 2.1 Hz, 1H, ArH), 6.83 (d, *J* = 2.1 Hz, 1H, ArH), 6.78 (d, *J* = 8.4 Hz, 1H, ArH) ppm. ^13^C-NMR (75 MHz, CD_3_OD): δ 130.8, 128.4, 126.4, 121.0, 111.0, 110.1 ppm. Anal. calcd (%) for C_6_H_5_ClN_2_O_2_S: C, 35.22; H, 2.46; N, 13.69; S, 15.67; found: C, 34.98; H, 2.49; N, 13.42; S, 15.48.

##### 5-Bromo-6-methyl-1*H*,3*H*-2,1,3-benzothiadiazole-2,2-dioxide, **2a**

The product was obtained starting from intermediate **12** following Procedure D. The crude residue was purified by flash column chromatography (DCM/MeOH 95:5) to yield the desired compound as a white solid. Yield: 85%. TLC (DCM/MeOH 95:5): R_f_: 0.19. Mp: 185 °C. ^1^H-NMR (300 MHz, CD_3_OD): δ 6.99 (s, 1H, ArH), 6.77 (s, 1H, ArH), 2.32 (s, 3H, CH_3_) ppm. ^13^C-NMR (75 MHz, CD_3_OD): δ 130.5, 129.5, 129.0, 115.2, 113.6, 112.0, 21.3 ppm. Anal. calcd (%) for C_7_H_7_BrN_2_O_2_S: C, 31.95; H, 2.68; N, 10.65; S, 12.19; found: C, 32.08; H, 2.75; N, 10.29; S, 12.31.

##### 1,3-Bis[(4-methoxyphenyl)methyl]-5-nitro-2,1,3-benzothiadiazole-2,2-dioxide, **13**

Procedure E: To a stirred solution of **1d** (140 mg, 0.65 mmol) in dry DMF (2 mL), dried K_2_CO_3_ (179.7 mg, 1.3 mmol) and PMB-Br (313.6 mg, 1.56 mmol) were added. The mixture was stirred at 80 °C for 2 h and then cooled to room temperature, diluted with H_2_O (6 mL), and extracted with EtOAc (2 × 5 mL). The collected organic layers were dried over anhydrous Na_2_SO_4_, filtered, and evaporated in vacuo. The crude residue was purified by flash chromatography (hexane/EtOAc 8:2) to give intermediate **13** as a yellow solid. Yield: 90%. TLC (hexane/EtOAc 8:2): R_f_: 0.28. Mp: 116 °C. ^1^H-NMR (300 MHz, CDCl_3_): δ 7.71 (dd, *J* = 8.8, 2.2 Hz, 1H, ArH), 7.38–7.33 (m, 3H, ArH), 7.30 (d, *J* = 8.8 Hz, 2H, ArH), 6.85 (d, *J* = 8.8 Hz, 2H, ArH), 6.83 (d, *J* = 8.8 Hz, 2H, ArH), 6.50 (d, *J* = 8.8 Hz, 1H, ArH), 4.86 (s, 4H, CH_2_), 3.73 (s, 3H, CH_3_), 3.72 (s, 3H, CH_3_) ppm. ^13^C-NMR (75 MHz, CDCl_3_): δ 159.88, 159.86, 142.1, 133.7, 129.3, 129.1, 128.7, 124.96, 124.93, 118.4, 114.59, 114.56, 107.3, 103.7, 55.3, 46.5, 46.2 ppm.

##### 5-Amino-1,3-bis[(4-methoxyphenyl)methyl]-2,1,3-benzothiadiazole-2,2-dioxide, **14**

Intermediate **13** was reacted for 4 h following Procedure C. The crude residue was purified by flash column chromatography (cyclohexane/EtOAc from 1:1 to 4:6) to give the desired product as a yellow oil. Yield: 46%. TLC (cyclohexane/EtOAc 6:4): R_f_: 0.23. ^1^H-NMR (300 MHz, CDCl_3_): δ 7.32–7.25 (m, 4H, ArH), 6.84–6.77 (m, 4H, ArH), 6.25 (d, *J* = 8.3 Hz, 1H, ArH), 6.01 (dd, *J* = 8.3, 2.3 Hz, 1H, ArH), 5.85 (d, *J* = 2.3 Hz, 1H, ArH), 4.68 (s, 2H, CH_2_), 4.67 (s, 2H, CH_2_), 3.71 (s, 6H, CH_3_), 3.31 (br s, 2H, NH_2_) ppm. ^13^C-NMR (75 MHz, CDCl_3_): δ 159.40, 159.39, 141.7, 130.8, 129.0, 128.8, 127.0, 126.7, 121.9, 114.3, 114.2, 110.2, 107.5, 97.3, 55.29, 55.28, 47.2, 46.1 ppm.

##### *N*-{1,3-Bis[(4-methoxyphenyl)methyl]-2,2-dioxo-2,1,3-benzothiadiazol-5-yl}acetamide, **15**

Intermediate **14** (114.9 mg, 0.27 mmol) was dissolved in acetone (2 mL), and the mixture was cooled to 0 °C in an ice bath. Acetic anhydride (41.3 mg, 0.41 mmol) was added dropwise, and the solution was stirred at room temperature for 2 h. After completion, the solvent was removed in vacuo and the residue was diluted with 1 M NaOH and extracted with EtOAc (2 × 3 mL). The collected organic layers were dried over anhydrous Na_2_SO_4_, filtered, and concentrated in vacuo. The crude residue was purified by flash column chromatography (cyclohexane/EtOAc from 5:5 to 4:6) to obtain intermediate **15** as a dark green solid. Yield: 98%. TLC (cyclohexane/EtOAc 4:6): R_f_: 0.22. Mp: 153 °C. ^1^H-NMR (300 MHz, CDCl_3_): δ 7.43–7.37 (m, 5H, ArH and NH), 7.11 (d, *J* = 2.1 Hz, 1H, ArH), 6.94–6.85 (m, 4H, ArH), 6.73 (dd, *J* = 8.4, 2.1 Hz, 1H, ArH), 6.44 (d, *J* = 8.4 Hz, 1H, ArH), 4.82 (s, 2H, CH_2_), 4.81 (s, 2H, CH_2_), 3.81 (s, 3H, OC*H*_3_), 3.78 (s, 3H, OC*H*_3_), 2.06 (s, 3H, CH_3_) ppm. ^13^C-NMR (75 MHz, CDCl_3_): δ 168.4, 159.49, 159.47, 132.4, 129.5, 129.2, 129.0, 126.4, 126.3, 125.7, 114.3, 112.8, 108.7, 101.8, 55.29, 55.25, 46.4, 46.1, 24.3 ppm.

##### *N*-(2,2-Dioxo-1H,3H-2,1,3-benzothiadiazol-5-yl)acetamide, **1f**

Intermediate **15** was reacted for 8 h at room temperature following Procedure D. The crude residue was purified by flash column chromatography (DCM/MeOH from 95:5 to 9:1) to afford a white foam. Yield: 52%. TLC (DCM/MeOH 85:15): R_f_: 0.37. ^1^H-NMR (300 MHz, CD_3_OD): δ 7.14 (d, *J* = 2.1 Hz, 1H, ArH), 6.83 (dd, *J* = 8.4, 2.1 Hz, 1H, ArH), 6.64 (d, *J* = 8.4 Hz, 1H, ArH), 1.99 (s, 3H, CH_3_) ppm. ^13^C-NMR (75 MHz, CD_3_OD): δ 170.0, 133.0, 130.3, 126.2, 113.1, 110.3, 103.3, 22.2 ppm. Anal. calcd (%) for C_8_H_9_N_3_O_3_S: C, 42.28; H, 3.99; N, 18.49; O, 21.12; S, 14.11; found: C, 42.37; H, 4.02; N, 18.65; S, 14.32.

##### 4-Bromo-*N*-[(4-methoxyphenyl)methyl]-2-nitroaniline, **16**

A mixture of 2,5-dibromo-nitrobenzene (200 mg, 0.71 mmol) and PMB-NH_2_ (293 mg, 2.14 mmol) was heated at 160 °C for 8 h. After cooling, the reaction was diluted with H_2_O (4 mL) and extracted with EtOAc (2 × 4 mL). The collected organic layers were washed with 0.5 M HCl (1 × 8 mL) and then dried over anhydrous Na_2_SO_4_, filtered, and concentrated in vacuo. The crude residue was purified by flash chromatography (cyclohexane/EtOAc 8:2) to afford the desired compound as an orange solid. Yield: 95%. TLC (cyclohexane/EtOAc 9:1): R_f_: 0.35. Mp: 121 °C. ^1^H-NMR (300 MHz, CDCl_3_): δ 8.25 (br s, 1H, NH), 8.23 (d, *J* = 2.4 Hz, 1H, ArH), 7.35 (dd, *J* = 9.2, 2.4 Hz, 1H, ArH), 7.16 (d, *J* = 8.8 Hz, 2H, ArH), 6.81 (d, *J* = 8.8 Hz, 2H, ArH), 6.65 (d, *J* = 9.2 Hz, 1H, ArH), 4.37 (d, *J* = 5.5 Hz, 2H, CH_2_), 3.72 (s, 3H, CH_3_) ppm. ^13^C-NMR (75 MHz, CDCl_3_): δ 159.3, 144.1, 138.9, 132.5, 128.9, 128.7, 128.4, 116.0, 114.4, 106.8, 55.3, 46.7 ppm.

##### 4-Bromo-*N*1-[(4-methoxyphenyl)methyl]benzene-1,2-diamine, **17**

Compound **16** was reacted according to Procedure C; the resulting crude residue was purified by flash column chromatography (cyclohexane/EtOAc from 7.5:2.5 to 7:3) to afford the desired product as a pale yellow solid. Yield: 54%. TLC (cyclohexane/EtOAc 7:3): R_f_: 0.36. Mp: 116 °C. ^1^H-NMR (300 MHz, CDCl_3_): δ 7.32 (d, *J* = 8.8 Hz, 2H, ArH), 6.96–6.88 (m, 3H, ArH), 6.86 (d, *J* = 2.4 Hz, 1H, ArH), 6.55 (d, *J* = 8.4 Hz, 1H, ArH), 4.23 (s, 2H, CH_2_), 3.84 (s, 3H, CH_3_), 3.57 (br s, 1H, NH), 3.40 (br s, 2H, NH_2_) ppm. ^13^C-NMR (75 MHz, CDCl_3_): δ 159.0, 136.6, 135.8, 131.0, 129.0, 123.0, 118.9, 114.1, 113.3, 110.7, 55.3, 48.1 ppm.

##### 5-Bromo-1-[(4-methoxyphenyl)methyl]-3*H*-2,1,3-benzothiadiazole-2,2-dioxide, **18**

Intermediate **17** was reacted for 2 h following Procedure A. The crude residue was purified by flash column chromatography (cyclohexane/EtOAc from 6:4 to 1:1), to afford compound **18** as a red solid. Yield: 60%. TLC (cyclohexane/EtOAc 6:4): R_f_: 0.19. Mp: 166 °C. ^1^H-NMR (300 MHz, CDCl_3_): δ 7.26 (d, *J* = 8.8 Hz, 2H, ArH), 6.87–6.82 (m, 2H, ArH), 6.80 (d, *J* = 8.8 Hz, 2H, ArH), 6.28 (d, *J* = 8.4 Hz, 1H, ArH), 4.69 (s, 2H, CH_2_), 3.70 (s, 3H, CH_3_) ppm. ^13^C-NMR (75 MHz, CDCl_3_): δ 159.6, 129.7, 129.0, 127.8, 125.8, 125.5, 114.4, 114.3, 113.7, 110.1, 55.3, 46.0 ppm.

##### 5-Bromo-1,3-bis[(4-methoxyphenyl)methyl]-2,1,3-benzothiadiazole-2,2-dioxide, **19**

The desired product was obtained starting from **18** following Procedure E. The crude residue was purified by flash column chromatography (cyclohexane/EtOAc 8:2) to afford a pale yellow solid. Yield: 98%. TLC (cyclohexane/EtOAc 7:3): R_f_: 0.59. Mp: 134 °C. ^1^H-NMR (300 MHz, CDCl_3_): δ 7.48–7.36 (m, 4H, ArH), 6.98–6.84 (m, 5H, ArH), 6.70 (d, *J* = 1.9 Hz, 1H, ArH), 6.42 (d, *J* = 8.4 Hz, 1H, ArH), 4.86 (s, 4H, CH_2_), 3.84 (s, 3H, CH_3_), 3.83 (s, 3H, CH_3_) ppm. ^13^C-NMR (75 MHz, CDCl_3_): δ 159.7, 159.6, 130.2, 129.0, 128.1, 125.9, 125.8, 124.1, 114.5, 114.4, 113.8, 111.5, 109.7, 55.3, 46.3 ppm.

##### Ethyl (2*E*)-3-{1,3-bis[(4-methoxyphenyl)methyl]-2,2-dioxo-2,1,3-benzothiadiazol-5-yl} prop-2-enoate, **20**

To a solution of intermediate **19** (200 mg, 0.41 mmol) in DMF (2.0 mL), triethylamine (0.28 mL, 2.05 mmol), ethyl acrylate (0.17 mL, 1.64 mmol), tri-*o*-tolylphosphine (74.8 mg, 0.25 mmol), and tris(dibenzylideneacetone)dipalladium(0) (75.1 mg, 0.08 mmol) were added, and the mixture was heated to 80 °C for 20 h. The reaction was then cooled to ambient temperature, and DMF was evaporated. The residue was diluted with H_2_O (3 mL) and extracted with EtOAc (3 × 3 mL). The collected organic layers were dried over Na_2_SO_4_, filtered, concentrated in vacuo, and purified by flash column chromatography (cyclohexane/EtOAc 8:2) to afford the desired product as a yellow foam. Yield: 50%. TLC (cyclohexane/EtOAc 7:3): R_f_: 0.31. ^1^H-NMR (300 MHz, CDCl_3_): δ 7.39 (d, *J* = 16.0 Hz, 1H, CH), 7.35–7.30 (m, 4H, ArH), 6.90 (dd, *J* = 8.2, 1.6 Hz, 1H, ArH), 6.87–6.80 (m, 4H, ArH), 6.62 (d, *J* = 1.6 Hz, 1H, ArH), 6.46 (d, *J* = 8.2 Hz, 1H, ArH), 6.04 (d, *J* = 16.0 Hz, 1H, CH), 4.80 (s, 2H, CH_2_), 4.79 (s, 2H, CH_2_), 4.14 (q, *J* = 7.1 Hz, 2H, OC*H*_2_) 3.74 (s, 3H, CH_3_), 3.72 (s, 3H, CH_3_), 1.23 (t, *J* = 7.1 Hz, 3H, CH_3_). ^13^C-NMR (75 MHz, CDCl_3_): δ 167.0, 159.63, 159,62, 144.1, 130.7, 129.5, 128.99, 128.95, 128.2, 125.95, 125.93, 122.8, 116.5, 114.5, 114.4, 108.4, 106.9, 60.5, 55.3, 46.3, 46.2, 14.3 ppm.

##### (2*E*)-3-{1,3-Bis[(4-methoxyphenyl)methyl]-2,2-dioxo-2,1,3-benzothiadiazol-5-yl} prop-2-enoic acid, **21**

To a solution of intermediate **20** (53.4 mg, 0.105 mmol) in MeOH (2 mL), 3 M NaOH (1 mL) was added, and the mixture was stirred at 80 °C for 3 h. After cooling, MeOH was evaporated under vacuum and 2 M HCl (1.5 mL) was added until pH 2. Extraction with EtOAc (3 × 3 mL) was carried out, and the collected organic layers were dried over Na_2_SO_4_, filtered, and evaporated in vacuo to afford the acid **21** as a pale yellow solid, which was used in the next step for further purification. Yield: quantitative. TLC (DCM/MeOH 95:5): R_f_: 0.26. Mp: 125–127 °C. ^1^H-NMR (300 MHz, CDCl_3_): δ 7.55 (d, *J* = 16 Hz, 1H, CH), 7.47–7.34 (m, 4H, ArH), 7.00 (dd, *J* = 8.1, 1.6 Hz, 1H, ArH), 6.97–6.84 (m, 4H, ArH), 6.70 (d, *J* = 1.6 Hz, 1H, ArH), 6.54 (d, *J* = 8.1 Hz, 1H, ArH), 6.11 (d, *J* = 16 Hz, 1H, CH), 4.89 (s, 4H, CH_2_), 3.82 (s, 3H, CH_3_), 3.80 (s, 3H, CH_3_) ppm. ^13^C-NMR (75 MHz, CDCl_3_): δ 172.35, 159.85, 146.87, 131.29, 129.71, 129.21, 127.5, 126.04, 123.58, 115.33, 114.66, 108.64, 107.23, 55.45, 46.40 ppm.

##### (2*E*)-3-{1,3-Bis[(4-methoxyphenyl)methyl]-2,2-dioxo-2,1,3-benzothiadiazol-5-yl}-*N*-(3-methylbutyl)prop-2-enamide, **22**

Procedure F: To a mixture of **21** (50.5 mg, 0.105 mmol) in DCM (1 mL), isopentylamine (13.7 mg, 0.15 mmol), *N*,*N*-diisopropylethylamine (DIPEA, 27.1 mg, 0.21 mmol), and HATU (47.9 mg, 0.126 mmol) were added. The reaction mixture was stirred at room temperature for 16 h; after completion, the solvent was evaporated and the residue was diluted with H_2_O (3 mL) and extracted with EtOAc (3 × 3 mL). The organic layers were washed with 1 M HCl (1 × 3 mL) and then dried over Na_2_SO_4_, filtered, and concentrated in vacuo. Purification by flash column chromatography (DCM/MeOH 95:5) afforded compound **22** as a yellow oil. Yield: 62%. TLC (DCM/EtOAc 9:1): R_f_: 0.55. ^1^H-NMR (300 MHz, CDCl_3_): δ 7.45–7.36 (m, 5H, ArH), 7.00–6.87 (m, 5H, ArH, CH), 6.67 (d, *J* = 1.6 Hz, 1H, ArH), 6.53 (d, *J* = 8.2 Hz, 1H, ArH), 6.09 (d, *J* = 15.5 Hz, 1H, CH), 5.62 (br t, *J* = 5.7 Hz, 1H, NH), 4.87 (s, 4H, CH_2_), 3.82 (s, 3H, OC*H*_3_), 3.81 (s, 3H, OC*H*_3_), 3.40–3.35 (m, 2H, CH_2_), 1.74–1.56 (m, 1H, CH), 1.47–1.41 (m, 2H, CH_2_), 0.91 (d, *J* = 6.6 Hz, 6H, CH_3_) ppm. ^13^C-NMR (75 MHz, CDCl_3_): δ 165.8, 159.58, 159.57, 140.3, 130.1, 129.5, 128.99, 128.92, 128.7, 128.6, 126.1, 126.0, 122.0, 119.3, 114.43, 114.37, 114.0, 108.5, 107.1, 55.3, 46.23, 46.17, 38.6, 38.0, 25.9, 22.4 ppm.

##### (2*E*)-3-{1,3-Bis[(4-methoxyphenyl)methyl]-2,2-dioxo-2,1,3-benzothiadiazol-5-yl}-*N*-phenylprop-2-enamide, **23**

To a stirred solution of **21** (50 mg, 0.104 mmol) in DCM (2.5 mL), aniline (0.03 mL, 0.32 mmol)**,** TBTU (46 mg, 0.142 mmol), and *N*-methylmorpholine (up to pH 7) were added. The reaction mixture was stirred at room temperature for 24 h. After completion, the product was extracted with DCM (3 × 3 mL), and the organic layer was washed with 1 M HCl (1 × 3 mL) and then with a saturated solution of NaHCO_3_ (1 × 3 mL) and finally with brine (1 × 3 mL). The organic phase was dried over anhydrous Na_2_SO_4_, filtered, and concentrated in vacuo. Purification by flash column chromatography (cyclohexane/EtOAc 7:3) yielded intermediate **23** as a white solid. Yield: 69%. TLC (cyclohexane/EtOAc 6:4): R_f_: 0.47. Mp: 180–182 °C. ^1^H-NMR (300 MHz, CDCl_3_): δ 7.57 (br s, 1H, NH), 7.52 (d, *J* = 15.4 Hz, 1H, CH), 7.43–7.27 (m, 8H, ArH), 7.11 (t, *J* = 7.4, 1H, ArH), 6.98 (dd, *J* = 8.1, 1.6 Hz, 1H, ArH), 6.96–6.87 (m, 4H, ArH), 6.67 (d, *J* = 1.6 Hz, 1H, ArH), 6.53 (d, *J* = 8.1 Hz, 1H, ArH), 6.21 (d, *J* = 15.4 Hz, 1H, CH), 4.87 (s, 4H, CH_2_), 3.81 (s, 3H, CH_3_), 3.80 (s, 3H, CH_3_) ppm.

##### 3-(2,2-Dioxo-1*H*,3*H*-2,1,3-benzothiadiazol-5-yl)-*N*-(3-methylbutyl)propanamide, **1g**

Procedure G: Intermediate **22** (100 mg, 0.182 mmol) was dissolved in EtOH (10 mL), and the solution was degassed by bubbling Ar for 10 min. Pd/C (10% w/w) was then added, and the mixture was stirred at room temperature under an H_2_ atmosphere (3 atm) for 48 h. After completion, the reaction mixture was filtered through celite, and the filtrate was evaporated under vacuum. The crude residue was purified by flash column chromatography (DCM/MeOH 95:5 to 9:1) to obtain product **1g** as a pale brown oil. Yield: 3%. TLC (DCM/MeOH 9:1): R_f_: 0.41. ^1^H-NMR (300 MHz, CD_3_OD): δ 6.78–6.55 (m, 3H, ArH), 3.12 (t, *J* = 7.5 Hz, 2H, CH_2_), 2.82 (t, *J* = 7.5 Hz, 2H, CH_2_), 2.41 (t, *J* = 7.5 Hz, 2H, CH_2_), 1.55–1.37 (m, 1H, CH), 1.35–1.18 (m, 2H, CH_2_), 0.86 (d, *J* = 6.6 Hz, 6H, CH_3_) ppm. ^13^C-NMR (75 MHz, CDCl_3_): δ 173.5, 134.6, 130.2, 128.2, 121.2, 110.2, 37.8, 37.1, 31.4, 25.2, 21.3 ppm. Anal. calcd (%) for C_14_H_21_N_3_O_3_S: C, 54.00; H, 6.80; N, 13.49; S, 10.30; found: C, 54.08; H, 6.95; N, 13.27; S, 10.41.

##### 3-(2,2-Dioxo-1*H*,3*H*-2,1,3-benzothiadiazol-5-yl)-*N*-phenylpropanamide, **1h**

The compound was obtained by reacting **23** at 50 °C for 5 h following Procedure G. The crude residue was purified by flash column chromatography (DCM/MeOH 9:1) to afford a white solid. Yield: 10%. TLC (DCM/MeOH 9:1): R_f_: 0.40. Mp: 192–197 °C. ^1^H-NMR (300 MHz, CD_3_OD): δ 7.51–7.45 (m, 2H, ArH), 7.33–7.22 (m, 2H, ArH), 7.13–6.99 (m, 1H, ArH), 6.80 (dd, *J* = 8.1, 1.7 Hz, 1H, ArH), 6.76–6.70 (m, 2H, ArH) 2.93 (t, *J* = 7.6 Hz, 2H, CH_2_), 2.61 (t, *J* = 7.6 Hz, 2H, CH_2_) ppm. ^13^C-NMR (75 MHz, CD_3_OD): δ 172.1, 138.3, 134.8, 130.1, 128.3, 128.1, 123.8, 121.3, 120.0, 110.3, 38.7, 31.2 ppm. Anal. calcd (%) for C_15_H_15_N_3_O_3_S: C, 56.77; H, 4.76; N, 13.24; S, 10.10; found: C, 56.89; H, 4.81; N, 13.16; S, 10.14.

##### 1,3-Bis[(4-methoxyphenyl)methyl]-5-phenyl-2,1,3-benzothiadiazole-2,2-dioxide, **24**

A flask was charged with **19** (70 mg, 0.14 mmol), Na_2_CO_3_ (30 mg, 0.28 mmol), phenylboronic acid (18 mg, 0.15 mmol), and tetrakis(triphenylphosphine)palladium(0) (32 mg, 0.028 mmol) under an Ar atmosphere. Then, 1,4-dioxane (3 mL) and a drop of H_2_O were added. The resulting mixture was heated to reflux for 5 h. After completion, the reaction was cooled to room temperature, H_2_O was added (3 mL), and the mixture was extracted with EtOAc (3 × 2 mL). The collected organic layers were dried over Na_2_SO_4_, filtered, and evaporated in vacuo. The residue was purified by flash column chromatography (cyclohexane/EtOAc 7:3) to afford the desired product as a pale-yellow oil. Yield: 68%. TLC (cyclohexane/EtOAc 7:3): R_f_: 0.50. ^1^H-NMR (300 MHz, CDCl_3_): δ 7.38–7.32 (m, 4H, ArH), 7.30–7.23 (m, 4H, ArH, partially hidden by solvent peak), 7.22–7.17 (m, 1H, ArH), 6.93 (dd, *J* = 8.1, 1.8 Hz, 1H, ArH), 6.87–6.80 (m, 4H, ArH), 6.70 (d, *J* = 1.8 Hz, 1H, ArH), 6.52 (d, *J* = 8.1 Hz, 1H, ArH), 4.83 (s, 2H, CH_2_), 4.80 (s, 2H, CH_2_), 3.72 (s, 3H, CH_3_), 3.71 (s, 3H, CH_3_) ppm. ^13^C-NMR (75 MHz, CDCl_3_): δ 159.5, 140.7, 135.2, 129.7, 129.04, 129.02, 128.97, 128.8, 128.6, 127.1, 126.8, 126.4, 120.4, 114.37, 114.35, 114.31, 108.8, 107.4, 55.3, 46.33, 46.29 ppm.

##### 5-Phenyl-1*H*,3*H*-2,1,3-benzothiadiazole-2,2-dioxide, **1i**

Intermediate **24** was reacted for 3 h at room temperature following Procedure D. The crude residue was purified by flash column chromatography (cyclohexane/EtOAc 6:4) to afford a white solid. Yield: 20%. TLC (cyclohexane/EtOAc 6:4): R_f_: 0.42. Mp: 180–185 °C. ^1^H-NMR (300 MHz, CD_3_OD): δ 7.47–7.38 (m, 2H, ArH), 7.33–7.26 (m, 2H, ArH), 7.24–7.14 (m, 1H, ArH), 7.07 (dd, *J* = 8.1, 1.8 Hz, 1H, ArH), 6.96 (d, *J* = 1.8 Hz, 1H, ArH), 6.78 (d, *J* = 8.1 Hz, 1H, ArH) ppm. Anal. calcd (%) for C_12_H_10_N_2_O_2_S: C, 58.52; H, 4.09; N, 11.37; S, 13.02; found: C, 58.48; H, 4.11; N, 11.26; S, 13.18.

##### 1,3-Dibenzyl-2,1,3-benzothiadiazole-2,2-dioxide, **3**

The compound was obtained starting from **1a** and benzyl bromide following Procedure E. The crude residue was purified by flash column chromatography (hexane/EtOAc 9:1) to afford the desired product as a white solid. Yield: 90%. TLC (hexane/EtOAc 7:3): R_f_: 0.81. Mp: 134 °C. ^1^H-NMR (300 MHz, CDCl_3_): δ 7.56–7.48 (m, 4H, ArH), 7.45–7.39 (m, 4H, ArH), 7.38–7.32 (m, 2H, ArH), 6.86–6.81 (m, 2H, ArH), 6.59–6.54 (m, 2H, ArH), 4.96 (s, 4H, CH_2_) ppm. ^13^C-NMR (75 MHz, CDCl_3_): δ 134.6, 129.3, 128.9, 128.1, 127.5, 121.6, 108.5, 46.7 ppm. Anal. calcd (%) for C_20_H_18_N_2_O_2_S: C, 68.55; H, 5.18; N, 7.99; S, 9.15; found: C, 68.63; H, 5.17; N, 8.13; S, 9.17.

##### 1,3,5-Trimethyl-2,1,3-benzothiadiazole-2,2-dioxide, **2b**

In a flame-dried flask, **1b** (50 mg, 0.27 mmol) was solubilized in dry DMF under an Ar atmosphere; oven-dried K_2_CO_3_ (111.9 mg, 0.81 mmol) was then added, and the mixture was stirred for 30 min. After cooling to 0 °C on an ice bath, CH_3_I (0.050 mL, 0.81 mmol) was added dropwise, and the reaction was heated at 80 °C for 2 h. After completion, DMF was removed under reduced pressure and the remaining mixture was diluted with 1 M HCl (3 mL), and then the product was extracted with EtOAc (3 × 3 mL). The organic fractions were combined, dried over anhydrous Na_2_SO_4_, filtered, and evaporated under vacuum. The crude residue was purified by flash column chromatography (hexane/EtOAc 8:2) to afford product **2b** as a white foam. Yield: 87%. TLC (hexane/EtOAc 6:4): R_f_: 0.78. ^1^H-NMR (300 MHz, CDCl_3_): δ 6.81 (dd, *J* = 8.0, 1.5 Hz, 1H, ArH), 6.64 (d, *J* = 8.0 Hz, 1H, ArH), 6.58 (d, *J* = 1.5 Hz, 1H, ArH), 3.28 (s, 3H, NC*H*_3_), 3.27 (s, 3H, NC*H*_3_), 2.36 (s, 3H, CH_3_) ppm. ^13^C-NMR (75 MHz, CDCl_3_): δ 131.7, 130.4, 128.2, 122.0, 108.7, 107.7, 28.2, 27.9, 21.4 ppm. Anal. calcd (%) for C_9_H_12_N_2_O_2_S: C, 50.92; H, 5.70; N, 13.20; S, 15.11; found: C, 50.78; H, 5.63; N, 13.28; S, 15.17.

##### *N*-[(2,4-Dimethoxyphenyl)methyl]-2-nitroaniline, **25**

In a two-neck flask, a solution of 1-chloro-2-nitrobenzene (200 mg, 1.27 mmol) and DMB-NH_2_ (637 mg, 3.8 mmol) in EtOH was heated overnight at 100 °C. After completion, EtOH was evaporated under vacuum*,* and the residue was dissolved in EtOAc and washed with 1 M HCl (1 × 3 mL). The organic layer was dried over anhydrous Na_2_SO_4_, filtered, and concentrated in vacuo. The crude residue was purified by flash column chromatography (hexane/EtOAc 8:2) to give the desired compound as an orange solid. Yield: 80%. TLC (hexane/EtOAc 8:2): R_f_: 0.32. Mp: 85–87 °C. ^1^H-NMR (300 MHz, CDCl_3_): δ 8.43 (br s, 1H, NH), 8.20 (dd, *J* = 8.7, 1.7 Hz, 1H, ArH), 7.43–7.39 (m, 1H, ArH), 7.17 (d, *J* = 8.4 Hz, 1H, ArH), 6.91 (dd, *J* = 8.7, 1.7 Hz, 1H, ArH), 6.67–6.62 (m, 1H, ArH), 6.52 (d, *J* = 2.4, 1H, ArH), 6.46 (dd, *J* = 8.4, 2.4 Hz, 1H, ArH), 4.48 (d, *J* = 5.6 Hz, 2H, CH_2_), 3.88 (s, 3H, CH_3_), 3.83 (s, 3H, CH_3_) ppm.

##### *N*1-[(2,4-Dimethoxyphenyl)methyl]benzene-1,2-diamine, **26**

Intermediate **25** was reacted for 1 h at reflux following Procedure C. The crude residue was purified by flash column chromatography (cyclohexane/EtOAc from 7:3 to 6:4) to afford a yellow solid. Yield: 73%. TLC (cyclohexane/EtOAc 6:4): R_f_: 0.37. Mp: 107–112 °C. ^1^H-NMR (300 MHz, CDCl_3_): δ 7.27 (d, *J* = 8.2 Hz, 1H, ArH), 6.92–6.82 (m, 1H, ArH), 6.78 (d, *J* = 8.4 Hz, 1H, ArH), 6.77–6.73 (m, 2H, ArH), 6.56 (d, *J* = 2.4 Hz, 1H, ArH), 6.51 (dd, *J* = 8.2, 2.4 Hz, 1H, ArH), 4.30 (s, 2H, CH_2_), 3.88 (s, 3H, CH_3_), 3.86 (s, 3H, CH_3_), 3.52 (br s, 2H, NH_2_) ppm. ^13^C-NMR (75 MHz, CDCl_3_): δ 160.3, 158.6, 137.9, 134.8, 130.0, 120.5, 120.0, 118.8, 116.3, 112.7, 104.1, 98.7, 55.42, 55.40, 43.7 ppm.

##### 1-[(2,4-Dimethoxyphenyl)methyl]-3*H*-2,1,3-benzothiadiazole-2,2-dioxide, **27**

The product was obtained by reacting **26** for 2 h at 160 °C following Procedure A. The crude residue was purified by flash column chromatography (cyclohexane/EtOAc 6:4) to give the desired compound as a sticky yellow solid. Yield: 78%. TLC (cyclohexane/EtOAc 1:1): R_f_: 0.31. ^1^H-NMR (300 MHz, CDCl_3_): δ 7.33 (d, *J* = 8.4 Hz, 1H, ArH), 7.03–6.84 (m, 3H, ArH), 6.72 (dd, *J* = 7.3, 1.1 Hz, 1H, ArH), 6.52 (d, *J* = 2.4 Hz, 1H, ArH), 6.48 (dd, *J* = 8.4, 2.4 Hz, 1H, ArH), 4.86 (s, 2H, CH_2_), 3.90 (s, 3H, CH_3_), 3.82 (s, 3H, CH_3_) ppm.

##### Ethyl 2-{3-[(2,4-dimethoxyphenyl)methyl]-2,2-dioxo-2,1,3-benzothiadiazol-1-yl}acetate, **28**

Intermediate **27** (100 mg, 0.31 mmol), K_2_CO_3_ (55.74 mg, 0.403 mmol), and ethyl bromoacetate (62.8 mg, 0.38 mmol) were reacted according to Procedure E. The crude residue was purified by flash column chromatography (hexane/EtOAc 7:3) to afford the desired compound as a yellow solid. Yield: 90%. TLC (hexane/EtOAc 6:4): R_f_: 0.52. Mp: 128–132 °C. ^1^H-NMR (300 MHz, CDCl_3_): δ 7.34 (d, *J* = 8.4 Hz, 1H, ArH), 6.97–6.90 (m, 2H, ArH), 6.75–6.67 (m, 2H, ArH), 6.51 (d, *J* = 2.4 Hz, 1H, ArH), 6.48 (dd, *J* = 8.4, 2.4 Hz, 1H, ArH), 4.90 (s, 2H, CH_2_), 4.47 (s, 2H, CH_2_), 4.29 (q, *J* = 7.1 Hz, 2H, OCH_2_), 3.90 (s, 3H, OC*H*_3_), 3.82 (s, 3H, OC*H*_3_), 1.30 (t, *J* = 7.1 Hz, 3H, CH_3_) ppm.

##### 2-{3-[(2,4-Dimethoxyphenyl)methyl]-2,2-dioxo-2,1,3-benzothiadiazol-1-yl} acetic acid, **29**

To a solution of intermediate **28** (63 mg, 0.16mmol) in MeOH (2 mL), 2% aqueous K_2_CO_3_ (1 mL) was added, and the mixture was stirred at 50 °C for 2 h. After cooling, MeOH was evaporated under vacuum, and 2 M HCl (1.5 mL) was added until it reached pH 4. Extraction with EtOAc (3 × 3 mL) was carried out, and the collected organic layers were dried over anhydrous Na_2_SO_4_, filtered, and evaporated in vacuo to obtain the acid **29** as a yellow oil, which was used in the next step without any further purification. Yield: quantitative. TLC (DCM/MeOH 9:1): R_f_: 0.10. ^1^H-NMR (300 MHz, CDCl_3_): δ 9.91 (br s, 1H, COOH), 7.32 (d, *J* = 8.4 Hz, 1H, ArH), 6.99–6.91 (m, 2H, ArH), 6.79–6.68 (m, 2H, ArH), 6.51 (d, *J* = 2.4 Hz, 1H, ArH), 6.47 (dd, *J* = 8.4, 2.4 Hz, 1H, ArH), 4.90 (s, 2H, CH_2_), 4.52 (s, 2H, CH_2_), 3.89 (s, 3H, CH_3_), 3.81 (s, 3H, CH_3_) ppm.

##### 2-{3-[(2,4-Dimethoxyphenyl)methyl]-2,2-dioxo-2,1,3-benzothiadiazol-1-yl}-*N*-phenethylacetamide, **30**

Intermediate **29** and 2-phenylethanamine were reacted for 24 h following Procedure F. The crude residue was purified by flash column chromatography (hexane/EtOAc 6:4) to afford the desired compound as a yellow oil. Yield: 47%. TLC (DCM/MeOH 95:5): R_f_: 0.83. ^1^H-NMR (300 MHz, CDCl_3_): δ 7.30 (d, *J* = 8.4 Hz, 1H, ArH), 7.20–7.16 (m, 3H, ArH), 7.10–7.03 (m, 2H, ArH), 7.01–6.96 (m, 2H, ArH), 6.85–6.78 (m, 1H, ArH), 6.70–6.64 (m, 1H, ArH), 6.63 (br t, *J* = 5.8 Hz, 1H, NH), 6.53 (d, *J* = 2.4 Hz, 1H, ArH), 6.50 (dd, *J* = 8.4, 2.4 Hz, 1H, ArH), 4.88 (s, 2H, CH_2_), 4.34 (s, 2H, CH_2_), 3.92 (s, 3H, CH_3_), 3.83 (s, 3H, CH_3_), 3.56 (td, *J* = 7.0, 5.8 Hz, 2H, NHC*H*_2_), 2.78 (t, *J* = 7.0 Hz, 2H, CH_2_C*H*_2_) ppm.

##### 2-(2,2-Dioxo-3*H*-2,1,3-benzothiadiazol-1-yl)-*N*-(2-phenylethyl)acetamide, **4**

Intermediate **30** was reacted according to Procedure D. The crude residue was purified by flash column chromatography (DCM/MeOH 95:5) to give the product as a white solid. Yield: 88%. TLC (DCM/MeOH 9:1): R_f_: 0.41. Mp: 172 °C. ^1^H-NMR (300 MHz, CD_3_OD): δ 8.06 (t, *J* = 5.9 Hz, 1H, NH), 7.29–7.23 (m, 2H, ArH), 7.22–7.16 (m, 3H, ArH), 7.00–6.94 (m, 2H, ArH), 6.92–6.84 (m, 1H, ArH), 6.70–6.58 (m, 1H, ArH), 4.27 (s, 2H, CH_2_), 3.49 (td, *J* = 7.2, 5.9 Hz, 2H, NHC*H*_2_), 2.81 (t, *J* = 7.2 Hz, 2H, CH_2_C*H*_2_) ppm. ^13^C-NMR (75 MHz, CD_3_OD): δ 167.6, 138.8, 131.3, 128.5, 128.1, 126.0, 121.9, 121.7, 110.5, 108.2, 44.2, 40.6, 35.0 ppm. Anal. calcd (%) for C_16_H_17_N_3_O_3_S: C, 57.99; H, 5.17; N, 12.68; S, 9.68; found: C, 58.05; H, 5.21; N, 12.49; S, 9.72.

##### 2,3-Dihydro-1*H*-1,3-benzodiazol-2-one, **5**

In a two-neck flamed-dried flask, a solution of *o*-phenylenediamine (30 mg, 0.28 mmol) and carbonyldiimidazole (48 mg, 0.30 mmol) in dry THF was heated at reflux overnight under an Ar atmosphere. After completion, the reaction was cooled to room temperature, and the solvent was evaporated under vacuum. The residue was acidified to pH 2 with 2 M HCl, and the precipitate was filtered off. The filtrate was dried in vacuo, and a white solid was obtained. The crude residue was purified by flash column chromatography (DCM/MeOH 95:5) to obtain product **5**. Yield: 78%. TLC (DCM/MeOH 9:1): R_f_: 0.22. Mp: 305–308 °C. ^1^H-NMR (300 MHz, CD_3_OD): δ 7.06–7.04 (m, 4H, ArH) ppm. ^13^C-NMR (75 MHz, CDCl_3_): δ 156.6, 129.4, 121.1, 108.9 ppm. Anal. calcd (%) for C_7_H_6_N_2_O: C, 62.68; H, 4.51; N, 20.88; found: C, 62.72; H, 4.58; N, 20.87.

### 3.3. Single Crystal X-ray Analysis

Diffraction data for compound **1** were collected by means of a Bruker AXS CCD-based three-circle diffractometer (Bruker AXS, Karlsruhe, Germany), working at ambient temperature with graphite-monochromatized Mo-Kα X-radiation (λ = 0.71073 Å).

X-ray diffraction data in the θ range of 2–30° were collected by acquiring 4 sets of 600 bidimensional CCD frames with the following operative conditions: omega rotation axis, scan width 0.3°, acquisition time 30 s, sample-to-detector distance 50 mm, phi angle fixed at four different values (0°, 90°, 180°, and 270°) for the four different sets.

Omega-rotation frames were processed with the SAINT software (Bruker) for data reduction (including intensity integration, background, Lorentz, and polarization corrections) and for determination of accurate unit-cell dimensions, obtained by the least-squares refinement of the positions of 6048 independent reflections with I > 10σ(I) in the θ range 2–25°. Absorption effects were empirically evaluated by the SADABS software [46], and absorption correction was applied to the data (0.847 and 0.973 min and max transmission factors, respectively).

The structures were solved by direct methods (*SIR-2014*) [47] and completed by iterative cycles of full-matrix least-squares refinement on *F*o^2^ and Δ*F* synthesis using the *SHELXL-17* [48] program (*WinGX* suite) [49]. The positions of hydrogen atoms were introduced at calculated positions in their described geometries and allowed to ride on the attached carbon atom with fixed isotropic thermal parameters (1.2 Ueq of the parent carbon atom).

These data can be obtained free of charge via www.ccdc.cam.ac.uk/conts/retrieving.html (or from the Cambridge Crystallographic Data Centre, 12, Union Road, Cambridge CB21EZ, UK; Fax: +44-1223-336-033; or deposit@ccdc.cam.ac.uk). CCDC Number 1,982,708 number contains the supplementary crystallographic data for this paper.

Crystal data for compound **1**: C_16_H_20_N_8_O_2_S_1_, *M*_r_ = 396.48 g/mol, Tetragonal, Space group *I41/a*, *a* = 14.1731 (7) Å, *b* = 14.1731 (7) Å, *c* = 18.0847 (9) Å, *V* = 3632.8 (5) Å^3^, *Z* = 8, *D_calc_* = 1.397 Mg/m^3^, *R* = 0.049 (2655 reflections), *wR2* = 0.133, *T* = 293 (2) K, *GOF* = 1.029. The reflections were collected in the range 1.78° ≤ θ ≤ 29.98° employing a 0.41 × 0.13 × 0.013 mm crystal.

### 3.4. Biological Evaluation

AlphaScreen is a bead-based non-radioactive assay system for the detection of biomolecular interactions in a microtiter plate format. The binding of biological partners brings donor and acceptor beads into close proximity and, as a result, a fluorescent signal between 520 and 620 nm was produced. The AlphaScreen-based assays [50,51] were performed in a final reaction volume of 25 μL of the assay buffer containing 10 mM HEPES–NaOH (pH 7.4), 50 mM NaCl, 1 mM EDTA (pH 8.0), 0.1% NP-40, and 10 ng/μL bovine serum albumin (BSA) in a 96-well microtiter plate at 25 °C. The pTyr peptide probes used in this study were 5-carboxyfluorescein (FITC)-GpYLPQTV for STAT3 and (FITC)-GpYDKPHVL for STAT1. Plasmid construction and protein expression were performed as previously described [52]; the experimental method is briefly summarized as follows. Considering that the expression of the full-length protein is troublesome, a truncated form of STAT3 (136-705) was constructed and chromatographically purified (AKTA FPLC system; GE Healthcare Bio-Sciences, Piscataway, NJ, USA). The soluble STAT3 fraction was loaded on a HisTrap™ column (GE Healthcare Bio-Sciences) and eluted with an imidazole gradient (50 mM Tris–HCl pH 7.4, 300 mM NaCl, 0.2 mM EDTA, 0.1% (*w*/*v*) NP-40, 5 mM β-mercaptoethanol, 10% (*v*/*v*) glycerol, and 10–300 mM imidazole). An SDS-PAGE stained with Coomassie Brilliant Blue (CBB) was performed to check the purity of the protein (see Figure S71). Subsequently, the protein was dialyzed (10 mM HEPES-NaOH pH 7.4, 50 mM NaCl, 10 mM β-mercaptoethanol, 0.1 mM EGTA, 0.02% (*w*/*v*) NP-40, 0.2 mM phenylmethylsulphonyl fluoride (PMSF), and 10% (*v*/*v*) glycerol).

Firstly, 75 nM of each SH2-containing protein were incubated with the test compound (at the following concentrations: 1, 3, 10, and 30 µM) for 15 min. Each protein sample was then incubated for 90 min with 50 nM of its corresponding FITC-pTyr peptide and simultaneously mixed with streptavidin-coated donor beads and anti-FITC acceptor beads before detection at 570 nm using EnVison Xcite (PerkinElmer, Waltham, MA, USA).

The same procedure was used to evaluate compound **1** activity versus STAT3 single-cysteine-residue mutants (Cys468A, Cys542A, Cys550A, Cys687A, and Cys712A). Briefly, 3 µL of compound **1** in DMSO (at 0.3, 1, 3, 10 and 30 µM concentration) were premixed with 1 µL of L-Cys in PBS and 16 µL of an AlphaScreen buffer. Then, the mixture was immediately (<5 min) mixed with the STAT3 mutant solution before adding the FITC-peptide.

### 3.5. Analytical Studies

#### 3.5.1. MS, UV, and LC Analyses

##### Sample Preparation

A compound **1** stock solution (1 mg/mL) was prepared in MeOH. The stock solution for GSH was prepared at the same concentration in deionized water.

An aqueous buffer (10 mM) for experiments at pH 2 was prepared with 10 mM H_3_PO_4_/NaH_2_PO_4_, whereas 10 mM NaH_2_PO_4_/Na_2_HPO_4_ and 10 mM NaHCO_3_/Na_2_CO_3_ were used for the physiological buffer (pH 7.4) and the basic buffer (pH 10), respectively. For experiments under non-oxidizing conditions, 5 mM Na_2_S_2_O_5_ was directly added into the buffers.

Reaction batches were prepared by spiking into the desired buffer aliquots of stock solutions of compound **1** and GSH down to 0.5 and 1 mM, respectively. All reactions were kept at 37 °C under gentle stirring for up to 24 h.

Before MS experiments, all samples were diluted 1:10 with an aqueous solution containing 30% acetonitrile and 0.1% formic acid to improve ionization.

Before LC injection, samples were diluted 1:5 with 24 mM HCl. No additional dilution/sample treatment was required for UV spectroscopy.

##### MS Method

MS analyses were performed on an LTQ-Orbitrap™ XL mass spectrometer (Thermo Fisher Scientific, Waltham, MA, USA) equipped with a Finnigan Ion Max 2 electro-spray source (ESI) endowed with a metal capillary that was 140 mm long and had a 160 µm inner diameter.

Samples were taken with a Hamilton glass syringe. The solution was injected by a pre-installed infuser at a flow rate of 5 µL/min. Nebulization was carried out in positive ion mode with a spray voltage of 4 kV, 10 units of sheath gas at a capillary temperature of 275 °C, and an offset tube lens of 74 V. Orbitrap™ analyzer acquired mass spectra with a scan range from 200 to 1000 *m*/*z*, recording 5 × 10^5^ ion per scan with a resolution of 100,000 (full-width at half-maximum at a 400 *m*/*z*).

##### UV Method

UV spectra were performed with a Shimadzu UV-1900 (Shimadzu, Kyoto, Japan) by using UV PROBE 2.7 software (Shimadzu). Analyses were done with 1.5 mL quartz cuvettes (Hellma, Müllheim, Germany), acquiring the spectra in a scan range from 200 to 600 nm.

##### LC Method

LC analysis was carried out with an HPLC Surveyor LC system (Thermo Finnigan) equipped with a quaternary pump, diode array (PDA) detector, autosampler, online degasser, and thermostat. Chromatographic separation was carried out with a Phenomenex^®^ Kinetex C18 reverse-phase column (Phenomenex, Torrance, CA, USA; 25 mm × 2.10 mm, 2.6 µm particle diameter with 100 Å pores). An OPTI-SOLV^®^ Mini Filter (0.5 mm; Sigma-Aldrich-Merck) was installed before the column to protect it from particulates. A gradient program reported in Table 4 was used at 200 µL/min flow rate; as a mobile phase, 1 mM HCl (pH 3) and acetonitrile were employed. The PDA detector worked in scan mode, acquiring UV spectra in a range from 200 to 600 nm.

#### 3.5.2. ^1^H-NMR Analysis

^1^H-NMR analysis was performed at 500 MHz with a Bruker FT-NMR AVANCE™ DRX500 spectrometer (Bruker, Billerica, MA, USA) using a 5 mm z-PFG (pulsed field gradient) broadband reverse probe at 298 K. The experimental procedure was designed as follows: a 10 mM pH 10 carbonate buffer solution in D_2_O was used as the solvent for NMR analysis. Na_2_S_2_O_5_ (20 mg, 0.1 mmol) was dissolved in 1 mL of the solvent, and (1) compound **1** (5 mg, 0.025 mmol), (2) GSH (16 mg, 0.05 mmol), or (3) compound **1** (5 mg, 0.025 mmol) and GSH (16 mg, 0.05 mmol) were dissolved in 0.7 mL of such a solution. Notably, when Na_2_S_2_O_5_ was added, the pH of the solution was slightly lowered below the value of 10 (pH ≤ 10). Each sample was immediately put into the NMR tube and analyzed (time 0) at 298 K. Then, they were heated at 37 °C in a thermostatic bath, and their ^1^H-NMR spectra were collected after different times at 298 K.

## 4. Conclusions

Benzothiadiazole derivative **1** was identified as a novel STAT3 inhibitor by structure-based virtual screening performed on the SH2 domain. The AlphaScreen-based assay confirmed, albeit indirectly, that compound **1** was able to inhibit STAT3 dimerization, exhibiting a good activity (STAT3 IC_50_ = 15.8 ± 0.6 µM versus STAT1 IC_50_ ˃ 50 µM). Therefore, a small set of derivatives was designed and synthesized through a flexible diversity-oriented synthetic approach when required. The compounds were tested to define the main structural features for the lead development. Among them, only compound **1d** exhibited a significant, albeit reduced, activity, thus suggesting a considerably “tight” SAR for this class of derivatives. Moreover, because compound **1**, like other known STAT3 inhibitors, probably binds to cysteine residues, MS, UV, LC, and NMR studies were performed to understand its mechanism of interaction. Mimicking the biological conditions, we conducted an MS analysis of compound **1** in the presence of GSH: We discovered that this inhibitor can covalently bind to cysteine residues through a Michael-like reaction, leading to the formation of some alkylation adducts.

In an attempt to confirm the results of the MS study, an NMR investigation was carried out to further characterize the adducts formed by the interaction of compound **1** with GSH. However, despite the same reaction conditions (pH, temperature, and use of an antioxidant) being employed, the intrinsic differences between the two techniques prevented us from obtaining additional evidence to support the MS conclusions. Notably, NMR spectroscopy is seldom used for this kind of study in the literature [16,18], which suggests that its inherent limitations make it unsuitable for such an application. Therefore, it would seem reasonable to claim that the NMR results neither supported nor disproved our hypotheses.

The research of STAT3 inhibitors has led to several potent anti-tumor candidates over the years (e.g., Stattic, S3I-201, and erasin). In this context, we are confident that our ongoing studies will allow us to develop and improve our lead compound **1** as a new PPI inhibitor endowed with a novel mechanism of interaction and characterized by a pH-dependent activation.

## Figures and Tables

**Table 1:** Inhibitory activities of compounds **1** and **I**–**III**.

Compound	Structure	% Inhibition
**1**	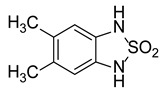	96.5
**I**	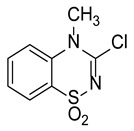	79.0
**II**	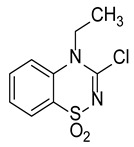	43.4
**III**	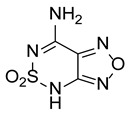	85.4

**Table 2:** Inhibitory activities of compounds **1**, **1a**–**i**, **2a**–**b**, and **3**–**6**. STAT3: signal transducer and activator of transcription 3.

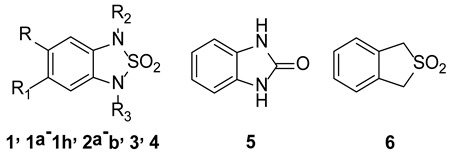		
Compound	Substituents	STAT3 % Inhibition (30 µM)
**1**	R, R_1_ = CH_3_; R_2_, R_3_ = H	96.5 ± 1.1 ^[a]^
**1a**	R, R_1_, R_2_, R_3_ = H	16.4 ± 0.8 ^[b]^
**1b**	R = H; R_1_ = CH_3_; R_2_, R_3_ = H	14.5 ± 0.6 ^[b]^
**1c**	R = H; R_1_ = CF_3_; R_2_, R_3_ = H	8.2 ± 1.6 ^[b]^
**1d**	R = H; R_1_ = NO_2_; R_2_, R_3_ = H	56.9 ± 3.6 ^[c]^
**1e**	R = H; R_1_ = Cl; R_2_, R_3_ = H	28.2 ± 1.4 ^[b]^
**1f**	R = H; R_1_ = NHCOCH_3_; R_2_, R_3_ = H	18.9 ± 2.3 ^[b]^
**1g**	R = H; R_1_ = (CH_2_)_2_CONH(CH_2_)_2_CH_3_; R_2_, R_3_ = H	11 ± 2.1 ^[b]^
**1h**	R = H; R_1_ = (CH_2_)_2_CONHPh; R_2_, R_3_ = H	n.a.
**1i**	R = H; R_1_ = Ph; R_2_, R_3_ = H	6.1 ± 0.9 ^[b]^
**2a**	R = CH_3_; R_1_ = Br; R_2_, R_3_ = H	25.6 ± 1.2 ^[b]^
**2b**	R = H; R_1_, R_2_, R_3_ = CH_3_	8.9 ± 1.1 ^[b]^
**3**	R, R_1_ = H; R_2_, R_3_ = CH_2_Ph	n.a.
**4**	R, R_1_, R_3_ = H; R_2_ = CH_2_CONH(CH_2_)_2_Ph	17.3 ± 1.3 ^[b]^
**5**	-	3.5 ± 5.6 ^[b]^
**6**	-	11.5 ± 0.8 ^[b]^

^[a]^ STAT3 inhibition: IC_50_ = 15.8 ± 0.6 µM; STAT1 inhibition: IC_50_ ˃ 50 µM. ^[b]^ STAT3 inhibition: IC_50_ ˃ 30 µM. ^[c]^ STAT3 inhibition: IC_50_ = 25.1 ± 2.6 µM; STAT1 inhibition: IC_50_ = 15.9 ± 2.0 µM. n.a.: not active.

**Table 3:** Sensitivity of single-mutated STAT3 proteins (Cys468, Cys542, Cys550, Cys687, and Cys712) to compound **1**. SH2 binding to the phosphotyrosine peptide was assessed by the AlphaScreen assay. The means ± SD were calculated from triplicate experiments.

Compound 1 vs. STAT3			
Mutated Cys	IC_50_ (µM)	Mutated Cys	IC_50_ (µM)
WT	15.8 ± 0.6	Cys550A	>30
Cys468A	>30	Cys687A	>30
Cys542A	>30	Cys712A	>30

**Table 4:** LC-UV gradient program.

Time (min)	1 mM HCl, pH 3 (A)	ACN (B)
0.00	95	5
1.00	95	5
16.00	30	70
16.50	30	70
16.51	95	5
22.00	95	5

## Supplementary Materials

The following are available online: Tables S1–S5: Top-ranked compounds from computational studies; Figure S1: Main SH2 domain residues involved in the interaction with compound **1**; Table S6: Pan-assay interference compound (PAINS) in silico prediction test, employing five on-line services (SmartsFilter, SwissADME, Zinc Patterns, FAF-Drugs4, and PAINS remover); Figures S2–S70: ^1^H and ^13^C-NMR spectra of synthesized compounds; Table S7: Effect of cysteine on the inhibitory activity of compound **1** versus STAT3; Table S8: Effect of the mutation of cysteine residues located in the vicinities of the SH2 domain on the inhibitory activity of compound **1** versus STAT3; Figure S71: CBB staining of the STAT3(136–705) and STAT5b(136–703) proteins. The purified soluble proteins (0.5 μg) were analyzed by SDS-PAGE; Table S9: Comparison of the best STAT3 complexes obtained by docking compound **1** into the pockets containing the mutated cysteines, considered in the mutagenesis study; Figure S72: Main interactions of compound **1** into the pocket lined by Cys712; Page S52: NMR studies; Figure S73–S75: ^1^H-NMR full spectra and expansions related to the NMR analysis of compound **1**/GSH.

## References

[1] Masciocchi D., Gelain A., Villa S., Meneghetti F., Barlocco D. (2011). Signal transducer and activator of transcription 3 (STAT3): A promising target for anticancer therapy. Future Med. Chem..

[2] Gelain A., Mori M., Meneghetti F., Villa S. (2018). Signal Transducer and Activator of Transcription Protein 3 (STAT3): An Update on its Direct Inhibitors as Promising Anticancer Agents. Curr. Med. Chem..

[3] Furtek S.L., Backos D.S., Matheson C.J., Reigan P. (2016). Strategies and Approaches of Targeting STAT3 for Cancer Treatment. ACS Chem. Biol..

[4] Lis C., Rubner S., Roatsch M., Berg A., Gilcrest T., Fu D., Nguyen E., Schmidt A.M., Krautscheid H., Meiler J. (2017). Development of Erasin: A chromone-based STAT3 inhibitor which induces apoptosis in Erlotinib-resistant lung cancer cells. Sci. Rep..

[5] Oh D.Y., Lee S.H., Han S.W., Kim M.J., Kim T.M., Kim T.Y., Heo D.S., Yuasa M., Yanagihara Y., Bang Y.J. (2015). Phase I study of OPB-31121, an oral STAT3 inhibitor, in patients with advanced solid tumors. Cancer Res. Treat..

[6] Keskin O., Gursoy A., Ma B., Nussinov R. (2008). Principles of protein-protein interactions: What are the preferred ways for proteins to interact?. Chem. Rev..

[7] Wells J.A., McClendon C.L. (2007). Reaching for high-hanging fruit in drug discovery at protein-protein interfaces. Nature.

[8] Shin D.S., Masciocchi D., Gelain A., Villa S., Barlocco D., Meneghetti F., Pedretti A., Han Y.M., Han D.C., Kwon B.M. (2010). Synthesis, modeling, and crystallographic study of 3,4-disubstituted-1,2,5-oxadiazoles and evaluation of their ability to decrease STAT3 activity. Medchemcomm.

[9] Masciocchi D., Villa S., Meneghetti F., Pedretti A., Barlocco D., Legnani L., Toma L., Kwon B.M., Nakano S., Asai A. (2012). Biological and computational evaluation of an oxadiazole derivative (MD77) as a new lead for direct STAT3 inhibitors. Medchemcomm.

[10] Masciocchi D., Gelain A., Porta F., Meneghetti F., Pedretti A., Celentano G., Barlocco D., Legnani L., Toma L., Kwon B.M. (2013). Synthesis, structure-activity relationships and stereochemical investigations of new tricyclic pyridazinone derivatives as potential STAT3 inhibitors. Medchemcomm.

[11] Meneghetti F., Villa S., Masciocchi D., Barlocco D., Toma L., Han D.C., Kwon B.M., Ogo N., Asai A., Legnani L. (2015). Ureido-Pyridazinone Derivatives: Insights into the Structural and Conformational Properties for STAT3 Inhibition. Eur. J. Org. Chem..

[12] Gabriele E., Porta F., Facchetti G., Galli C., Gelain A., Meneghetti F., Rimoldi I., Romeo S., Villa S., Ricci C. (2016). Synthesis of new dithiolethione and methanethiosulfonate systems endowed with pharmaceutical interest. Arkivoc.

[13] Porta F., Facchetti G., Ferri N., Gelain A., Meneghetti F., Villa S., Barlocco D., Masciocchi D., Asai A., Miyoshi N. (2017). An in vivo active 1,2,5-oxadiazole Pt(II) complex: A promising anticancer agent endowed with STAT3 inhibitory properties. Eur. J. Med. Chem..

[14] Colyer D.E., Nortcliffe A., Wheeler S. (2010). A general synthetic strategy for 1,3-dihydro-2,1,3-benzothiadiazole 2,2-dioxides (benzosulfamides). Tetrahedron Lett..

[15] Buettner R., Corzano R., Rashid R., Lin J., Senthil M., Hedvat M., Schroeder A., Mao A., Herrmann A., Yim J. (2011). Alkylation of cysteine 468 in stat3 defines a novel site for therapeutic development. ACS Chem. Biol..

[16] Heidelberger S., Zinzalla G., Antonow D., Essex S., Piku Basu B., Palmer J., Husby J., Jackson P.J.M., Rahman K.M., Wilderspin A.F. (2013). Investigation of the protein alkylation sites of the STAT3:STAT3 inhibitor Stattic by mass spectrometry. Bioorg. Med. Chem. Lett..

[17] Fletcher S., Page B.D.G., Zhang X., Yue P., Li Z.H., Sharmeen S., Singh J., Zhao W., Schimmer A.D., Trudel S. (2011). Antagonism of the Stat3-Stat3 Protein Dimer with Salicylic Acid Based Small Molecules. Chem. Med. Chem..

[18] Ball D.P., Lewis A.M., Williams D., Resetca D., Wilson D.J., Gunning P.T. (2016). Signal transducer and activator of transcription 3 (STAT3) inhibitor, S3I-201, acts as a potent and non-selective alkylating agent. Oncotarget.

[19] Becker S., Groner B., Müller C.W. (1998). Three-dimensional structure of the Stat3β homodimer bound to DNA. Nature.

[20] Goodsell D.S., Olson A.J. (1990). Automated docking of substrates to proteins by simulated annealing. Proteins Struct. Funct. Bioinform..

[21] Schmidhammer H., Hohenlohe-Oehringen K. (1983). Synthesis of dopa and dopamine analogs with acidic imido-functionalities in heterocyclic ring moieties instead of the phenolic hydroxyl groups. Sci. Pharm..

[22] Burke P.O., McDermott S.D., Hannigan T.J., Spillane W.J. (1984). Basicity of nitrogen-sulphur(VI) compounds. Part 6. Ionization of NN’-di-and N-mono-substituted sulphamides and dihydro-2,1,3-benzothiadiazoline and benzothiadiazine 2,2-dioxides (cyclic sulphamides). J. Chem. Soc. Perkin Trans..

[23] Farrugia L.J. (1999). WinGX suite for small-molecule single-crystal crystallography. J. Appl. Crystallogr..

[24] Kortylewski M., Jove R., Yu H. (2005). Targeting STAT3 affects melanoma on multiple fronts. Cancer Metastasis Rev..

[25] McMurray J.S., Klostergaard J. (2014). STAT3 Signaling in Cancer: Small Molecule Intervention as Therapy?. Anti-Angiogenesis Drug Discovery and Development.

[26] Dahlin J.L., Nissink J.W.M., Strasser J.M., Francis S., Higgins L., Zhou H., Zhang Z., Walters M.A. (2015). PAINS in the assay: Chemical mechanisms of assay interference and promiscuous enzymatic inhibition observed during a sulfhydryl-scavenging HTS. J. Med. Chem..

[27] FAF-Drugs3: A Web Server for Compound Property Calculation and Chemical Library Design—PubMed. https://pubmed.ncbi.nlm.nih.gov/25883137/.

[28] Baell J.B., Holloway G.A. (2010). New substructure filters for removal of pan assay interference compounds (PAINS) from screening libraries and for their exclusion in bioassays. J. Med. Chem..

[29] Daina A., Michielin O., Zoete V. (2017). SwissADME: A free web tool to evaluate pharmacokinetics, drug-likeness and medicinal chemistry friendliness of small molecules. Sci. Rep..

[30] Madeira F., Park Y.M., Lee J., Buso N., Gur T., Madhusoodanan N., Basutkar P., Tivey A.R.N., Potter S.C., Finn R.D. (2019). The EMBL-EBI search and sequence analysis tools APIs in 2019. Nucleic Acids Res..

[31] Wen B., Fitch W.L. (2009). Screening and characterization of reactive metabolites using glutathione ethyl ester in combination with Q-trap mass spectrometry. J. Mass Spectrom..

[32] Mayer R.J., Ofial A.R. (2019). Nucleophilicity of Glutathione: A Link to Michael Acceptor Reactivities. Angew. Chem.-Int. Ed..

[33] Maier G.P., Bernt C.M., Butler A. (2018). Catechol oxidation: Considerations in the design of wet adhesive materials. Biomater. Sci..

[34] Asiamah I., Krol E.S. (2018). Quadrupole linear ion-trap mass spectrometry studies on glutathione conjugates of nordihydroguaiaretic acid (NDGA) analogues reveals phenol-type analogues are without reactive metabolite-mediated toxic liability. Cogent Chem..

[35] Iverson S.L., Shen L., Anlar N., Bolton J.L. (1996). Bioactivation of estrone and its catechol metabolites to quinoid-glutathione conjugates in rat liver microsomes. Chem. Res. Toxicol..

[36] Toteva M.M., Richard J.P. (2011). The generation and reactions of quinone methides. Adv. Phys. Org. Chem..

[37] Ali A.M., Gómez-Biagi R.F., Rosa D.A., Lai P.-S., Heaton W.L., Park J.S., Eiring A.M., Vellore N.A., de Araujo E.D., Ball D.P. (2016). Disarming an Electrophilic Warhead: Retaining Potency in Tyrosine Kinase Inhibitor (TKI)-Resistant CML Lines While Circumventing Pharmacokinetic Liabilities. ChemMedChem.

[38] Vila-Viçosa D., Teixeira V.H., Santos H.A.F., Machuqueiro M. (2013). Conformational study of GSH and GSSG using constant-pH molecular dynamics simulations. J. Phys. Chem. B.

[39] Pedretti A., Villa L., Vistoli G. (2002). VEGA: A versatile program to convert, handle and visualize molecular structure on Windows-based PCs. J. Mol. Graph. Model..

[40] Gasteiger J., Marsili M. (1980). Iterative partial equalization of orbital electronegativity-a rapid access to atomic charges. Tetrahedron.

[41] MacKerell A.D., Bashford D., Bellott M., Dunbrack R.L., Evanseck J.D., Field M.J., Fischer S., Gao J., Guo H., Ha S. (1998). All-atom empirical potential for molecular modeling and dynamics studies of proteins. J. Phys. Chem. B.

[42] Foloppe N., Alexander D., MacKerell J. (2000). All-atom empirical force field for nucleic acids: I. Parameter optimization based on small molecule and condensed phase macromolecular target data. J. Comput. Chem..

[43] Phillips J.C., Braun R., Wang W., Gumbart J., Tajkhorshid E., Villa E., Chipot C., Skeel R.D., Kalé L., Schulten K. (2008). Scalable molecular dynamics with NAMD. J. Comput. Chem..

[44] Grotthuss M., Koczyk G., Pas J., Wyrwicz L., Rychlewski L. (2005). Ligand.Info Small-Molecule Meta-Database. Comb. Chem. High Throughput Screen..

[45] Pedretti A., Mazzolari A., Vistoli G. (2018). WarpEngine, a Flexible Platform for Distributed Computing Implemented in the VEGA Program and Specially Targeted for Virtual Screening Studies. J. Chem. Inf. Model..

[46] Krause L., Herbst-Irmer R., Sheldrick G.M., Stalke D. (2015). Comparison of silver and molybdenum microfocus X-ray sources for single-crystal structure determination. J. Appl. Crystallogr..

[47] Burla M.C., Caliandro R., Carrozzini B., Cascarano G.L., Cuocci C., Giacovazzo C., Mallamo M., Mazzone A., Polidori G. (2015). Crystal structure determination and refinement via SIR2014. J. Appl. Crystallogr..

[48] Sheldrick G.M. (2015). Crystal structure refinement with SHELXL. Acta Crystallogr. Sect. C Struct. Chem..

[49] Farrugia L.J. (2012). WinGX and ORTEP for Windows: An update. J. Appl. Crystallogr..

[50] Matsuno K., Masuda Y., Uehara Y., Sato H., Muroya A., Takahashi O., Yokotagawa T., Furuya T., Okawara T., Otsuka M. (2010). Identification of a new series of STAT3 inhibitors by virtual screening. ACS Med. Chem. Lett..

[51] Uehara Y., Mochizuki M., Matsuno K., Haino T., Asai A. (2009). Novel high-throughput screening system for identifying STAT3-SH2 antagonists. Biochem. Biophys. Res. Commun..

[52] Asai A., Takakuma K., Machida K., Liu B.A. (2017). Expression and Purification of Soluble STAT5b/STAT3 Proteins for SH2 Domain Binding Assay. SH2 Domains: Methods and Protocols.

