# Supplementary material for: Towards the Inhibition of Protein–Protein Interactions (PPIs) in STAT3: Insights into a New Class of Benzothiadiazole Derivatives
Source: Molecules. 2020 Jul 31;25(15):3509. doi: 10.3390/molecules25153509 (PMC7435819; doi:10.3390/molecules25153509)
Supplement: Supplementary file 1 [file molecules-25-03509-s001.pdf]

Towards the Inhibition of Protein-Protein Interactions (PPIs) in STAT3: Insights into a New Class of Benzothiadiazole Derivatives

Matteo Mori [1], Ettore Gilardoni [1], Luca Regazzoni [1,*], Alessandro Pedretti [1], Diego Colombo [2], Gary Parkinson [3], Akira Asai [4], Fiorella Meneghetti [1], Stefania Villa [1,*] and Arianna Gelain [1]

[1] Department of Pharmaceutical Sciences, University of Milan, via L. Mangiagalli 25, 20133 Milano, Italy; matteo.mori@unimi.it; ettore.gilardoni@unimi.it; alessandro.pedretti@unimi.it; fiorella.meneghetti@unimi.it; arianna.gelain@unimi.it

[2] Department of Medical Biotechnology and Translational Medicine. University of Milan, via C. Saldini 50, 20133 Milano, Italy; diego.colombo@unimi.it

[3] Department of Pharmaceutical and Biological Chemistry – UCL School of Pharmacy, University College London, 29/39 Brunswick Square, WC1N 1AX London, United Kingdom; gary.parkinson@ucl.ac.uk

[4] Center for Drug Discovery – Graduate School of Pharmaceutical Sciences, University of Shizuoka, 52-1 Yada, Suruga-ku, 422-8526 Shizuoka, Japan; aasai@u-shizuoka-ken.ac.jp

* Correspondence: luca.regazzoni@unimi.it (L.R.); stefania.villa@unimi.it (S.V.) Tel.: +39-02-50319368 (S.V.)

Supplementary Materials

Molecular Modeling......S2

Chemistry – ^{1}H- and ^{13}C-NMR spectra......S10

Biological Evaluation......S49

Computational studies on cysteine residues......S51

^{1}H-NMR studies......S53

Molecular Modeling

Table S1. Top-ranked compounds with molecular weight lower than 400 Daltons, including the sulfonamide moiety. The interaction energy was evaluated by AutoDock 4 and is indicated in Kcal/mol.

ID	Name	Structure	ID	Energy
**1**	17-Oxo-estra-1(10),2,4-trien-3-yl sulfamate	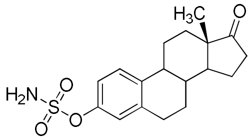	5444	-8.49
**2**	(3-(6-Amino-9*H*-purin-9-yl)cyclopentyl)methyl sulfamate	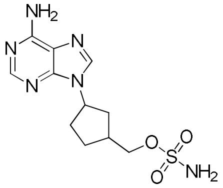	667	-7.16
**3**	(4-(6-(Dimethylamino)-9H-purin-9-yl)-2-cyclopenten-1-yl)methyl sulfamate	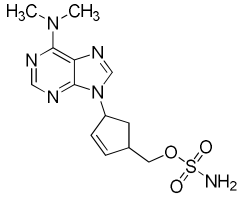	826	-6.48
**4**	(4-(2-Amino-6-hydroxy-9H-purin-9-yl)-2-cyclopenten-1-yl)methyl sulfamate	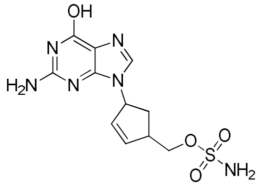	807	-6.43
**5**	2-((5-(Aminosulfonyl)-1,3,4-thiadiazol-2-yl)imino)pentanedioic acid	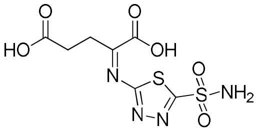	7475	-4.52

Table S2. Top-ranked compounds with molecular weight lower than 300 Daltons, including the cyclic sulfonamide moiety. The interaction energy was evaluated by AutoDock 4 and is indicated in Kcal/mol.

ID	Name	Structure	ID	Energy
**1**	4-((5-(Hydroxy(oxido)amino)-2-furyl)methylene)-3,5-diimino-1,2,6-thiadiazinane-1,1-dioxide	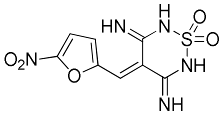	17152	-7.49
**2**	3*H*-[1,2,5]Oxadiazolo[3,4-c][1,2,6]thiadiazin-7(6*H*)-imine-5,5-dioxide	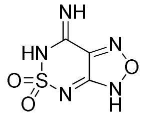	16346	-6.75
**3**	5,6-Dimethyl-1*H*,3*H*-2,1,3-benzothiadiazole-2,2-dioxide	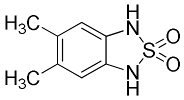	20093	-6.19
**4**	6-Chloro-3-(2-methylhydrazino)-1,4,2-benzodithiazine-7-carbonitrile-1,1-dioxide	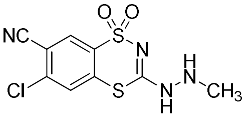	23145	-5.64
**5**	3-Hydrazino-4-methyl-4*H*-1,2,4-benzothiadiazine-1,1-dioxide	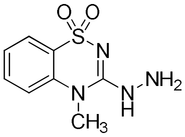	15657	-5.54
**6**	3-Chloro-4-methyl-4*H*-1,2,4-benzothiadiazine-1,1-dioxide	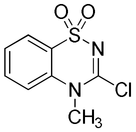	15487	-5.51
**7**	4-Methyl-6-(methylthio)-4*H*-pyrimido[4,5-e][1,2,4]thiadiazin-8-amine-1,1-dioxide	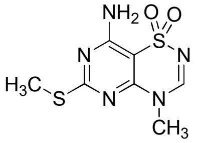	19594	-5.51
**8**	3-Chloro-4-ethyl-4*H*-1,2,4-benzothiadiazine-1,1-dioxide	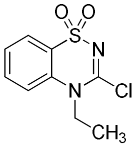	15485	-5.32
**9**	4-Ethyl-3-hydrazino-4*H*-1,2,4-benzothiadiazine-1,1-dioxide	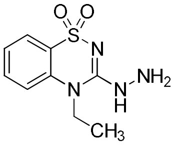	19155	-5.27
**10**	3-Methoxy-4a,5,6,7,8,9,10,10a-octahydrocycloocta[e][1,4,3]oxathiazine-1,1-dioxide	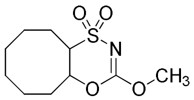	15897	-4.97

Table S3. Top-ranked compounds with molecular weight lower than 600 Daltons, including the sulfonic moiety. The interaction energy was evaluated by AutoDock 4 and is indicated in Kcal/mol.

ID	Name	Structure	ID	Energy
**1**	2-[(5*E*)-5-[[3-(Benzofuran-2-yl)-1-phenyl-pyrazol-4-yl]methylene]-4-oxo-2-thioxo-thiazolidin-3-yl]ethanesulfonic acid	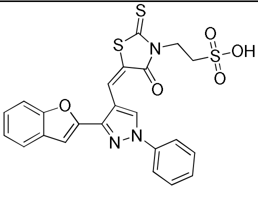	464405	-6.04
**2**	2-[(5*E*)-5-[[3-(7-Methoxybenzofuran-2-yl)-1-phenyl-pyrazol-4-yl]methylene]-4-oxo-2-thioxo-thiazolidin-3-yl]ethanesulfonic acid	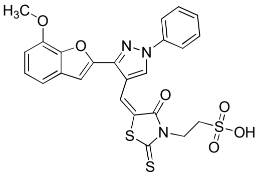	495695	-5.97
**3**	(*Z*)-4-(2-(3-(4-Nitrophenyl)-5-oxo-1-(4-phenylthiazol-2-yl)-1*H*-pyrazol-4(5*H*)-ylidene)hydrazinyl)benzenesulfonic acid	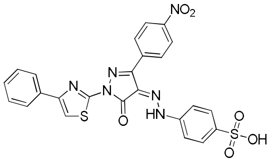	188838	-5.86
**4**	2-[(5*Z*)-5-(1-Benzyl-5-bromo-2-oxo-indolin-3-ylidene)-4-oxo-2-thioxo-thiazolidin-3-yl]ethanesulfonic acid	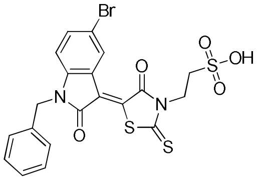	350292	-5.74
**5**	2-[(3*Z*)-5-Bromo-2-oxo-3-[4-oxo-3-(2-sulfoethyl)-2-thioxo-thiazolidin-5-ylidene]indolin-1-yl]acetic acid	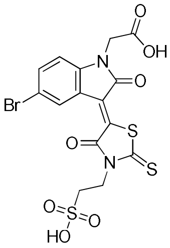	350288	-5.21

Table S4. Top-ranked compounds with molecular weight lower than 200 Daltons, including the 1,2-diketone moiety. The interaction energy was evaluated by AutoDock 4 and is indicated in Kcal/mol.

ID	Name	Structure	ID	Energy
**1**	5,6-Quinolinedione	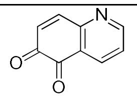	20120	-5.29
**2**	3,8-Dimethylnaphthalene-1,2-dione	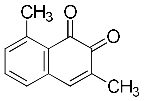	12839	-4.87
**3**	8-Methoxy-7-methyl-5,6-quinolinedione	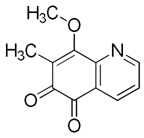	24935	-4.42
**4**	8-(Dimethylamino)-5,6-isoquinolinedione	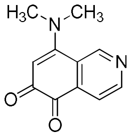	24703	-4.41
**5**	8-Methoxy-2-methyl-5,6-quinolinedione	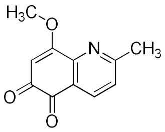	24907	-4.38
**6**	8-Methoxy-4-methyl-5,6-quinolinedione	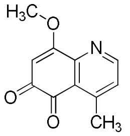	24920	-4.35

Table S5. Top-ranked compounds with molecular weight lower than 400 Daltons, including the phosphonate and phosphate moieties. The interaction energy was evaluated by AutoDock 4 and is indicated in Kcal/mol.

ID	Name	Structure	ID	Energy
**1**	(3-Hydroxy-4-oxo-2-phenyl-4*H*-chromen-8-yl)methylphosphonic acid	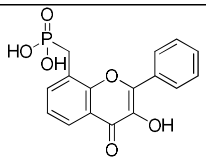	722	-8.41
**2**	1-Hydroxy-1-(hydroxy(2-hydroxy-3-(4-morpholinyl)propoxy)phosphoryl)ethylphosphonic acid	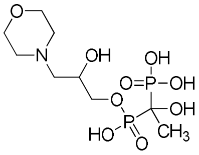	4309	-8.28
**3**	(5-(5-Benzyl-2,4-dioxo-3,4-dihydro-1(2*H*)-pyrimidinyl)tetrahydro-2-furanyl)methyl dihydrogen phosphate	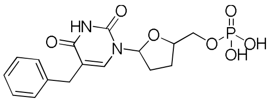	942	-7.64
**4**	(3-(7-Amino-3*H*-[1,2,3]triazolo[4,5-d]pyrimidin-3-yl)cyclopentyl)methylphosphonic acid	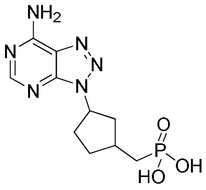	669	-7.54
**5**	1-Amino-2-(1*H*-indol-3-yl)ethylphosphonic acid	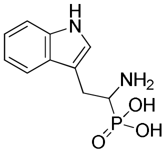	3918	-7.34
**6**	(3-(6-Amino-9*H*-purin-9-yl)cyclopentyl)methylphosphonic acid	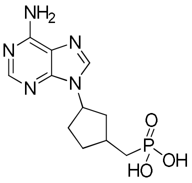	668	-7.17
**7**	(2-Amino-4-hydroxy-7,8-dihydro-6-pteridinyl)methyl trihydrogen diphosphate	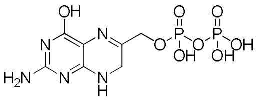	574	-7.13
**8**	(4-(7-Amino-3*H*-[1,2,3]triazolo[4,5-d]pyrimidin-3-yl)-2-cyclopenten-1-yl)methylphosphonic acid	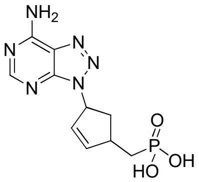	837	-7.07
**9**	2-Oxo-2*H*-chromen-7-yl dihydrogen phosphate	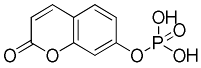	11820	-6.89

**10**	1-Amino-2-(3,4-dihydroxyphenyl)ethylphosphonic acid	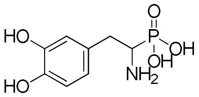	3919	-6.67
**11**	1-Amino-3-(methylsulfonimidoyl)propylphosphonic acid	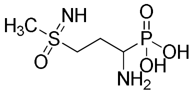	3936	-6.66
**12**	1-Amino-3-(benzylthio)propylphosphonic acid	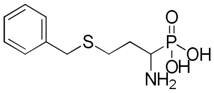	3931	-6.43
**13**	2-Oxo-2-(2-thienyl)ethylphosphonic acid	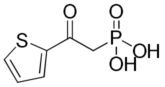	11810	-6.37
**14**	2-(Hydroxymethyl)-6-imino-2,3,3a,9a-tetrahydro-6*H*-furo[2',3':4,5][1,3]oxazolo[3,2-a]pyrimidin-3-yl dihydrogen phosphate	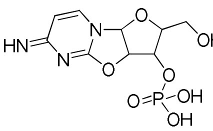	10157	-6.31
**15**	4-Amino-5-((1-methyl-2-oxo-2-((phosphonomethyl)amino)ethyl)amino)-5-oxopentanoic acid	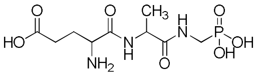	18669	-6.10
**16**	6-Phosphononorleucine	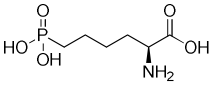	23584	-6.02
**17**	(2-((2-Amino-5-formyl-6-hydroxy-4-pyrimidinyl)amino)ethoxy)methylphosphonic acid	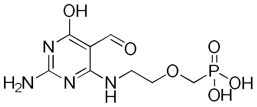	472	-5.85
**18**	(3-(5-Amino-4-(aminocarbonyl)-1H-imidazol-1-yl)cyclopentyl)methylphosphonic acid	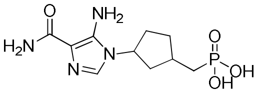	665	-5.78
**19**	(2-((2,5-Diamino-6-hydroxy-4-pyrimidinyl)amino)ethoxy)methylphosphonic acid	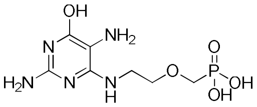	469	-5.69

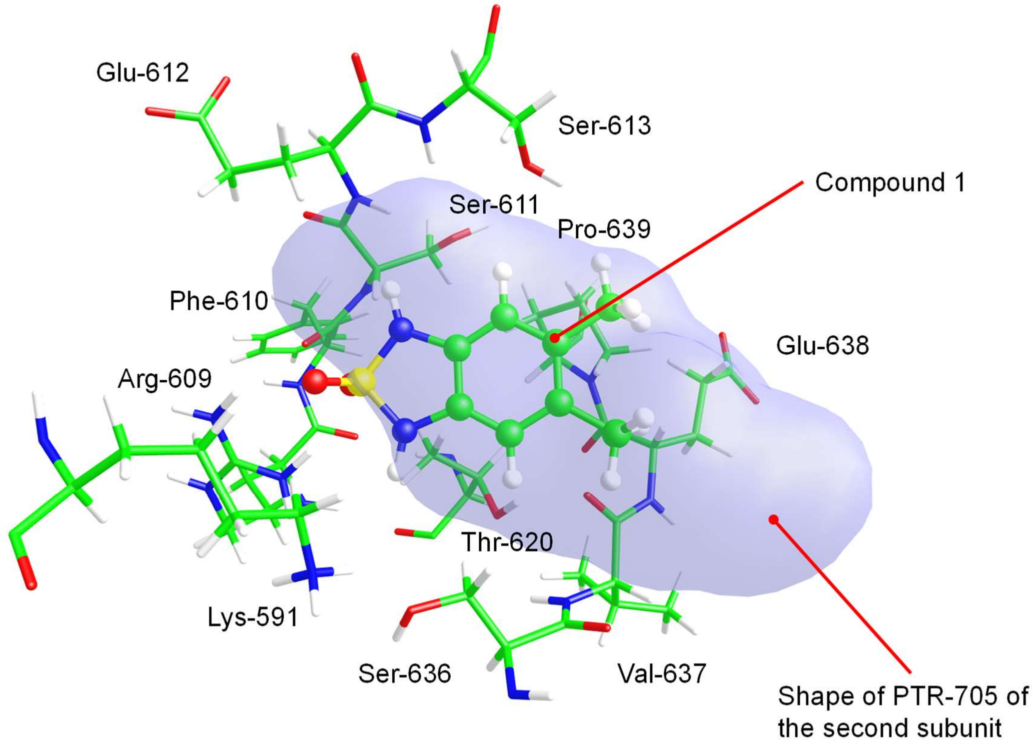

Figure S1. Main SH2 domain residues involved in the interaction with compound **1**. The shape of the phosphorylated Tyr-705 (PTR-705) of the second subunit in dimerized STAT3 is depicted in violet. In particular, compound **1** occupies most of the volume of PTR-705, and the sulfonamide moiety is inserted into the polar pocket lined by Arg-609 and Lys-591, usually occupied by the phosphoric group.

Table S6. Pan-assay interference compound (PAINS) *in silico* prediction test, employing five on-line services (SmartsFilter, SwissADME, Zinc Patterns, FAF-Drugs4, and PAINS remover).

Compound	SmartsFilter	SwissADME	Zinc Patterns	FAF-Drugs4	PAINS remover
**1**	**Yes**	**Yes**	**Yes**	No	No
**1a**	**Yes**	**Yes**	**Yes**	No	No
**1b**	**Yes**	**Yes**	**Yes**	No	No
**1c**	**Yes**	**Yes**	**Yes**	No	No
**1d**	**Yes**	**Yes**	**Yes**	No	No
**1e**	**Yes**	**Yes**	**Yes**	No	No
**1f**	**Yes**	**Yes**	**Yes**	No	No
**1g**	**Yes**	**Yes**	**Yes**	No	No
**1h**	**Yes**	**Yes**	**Yes**	No	No
**1i**	**Yes**	**Yes**	**Yes**	No	No
**2a**	**Yes**	**Yes**	**Yes**	No	No
**2b**	No	No	No	No	No
**3a**	No	No	No	No	No
**3b**	No	No	No	No	No
**4**	No	No	No	No	No
**5**	No	No	No	No	No

Chemistry - Experimental Data

S1. ¹H and ¹³C NMR spectra

S1.1. 5,6-Dimethyl-1*H*,3*H*-2,1,3-benzothiadiazole-2,2-dioxide (1)

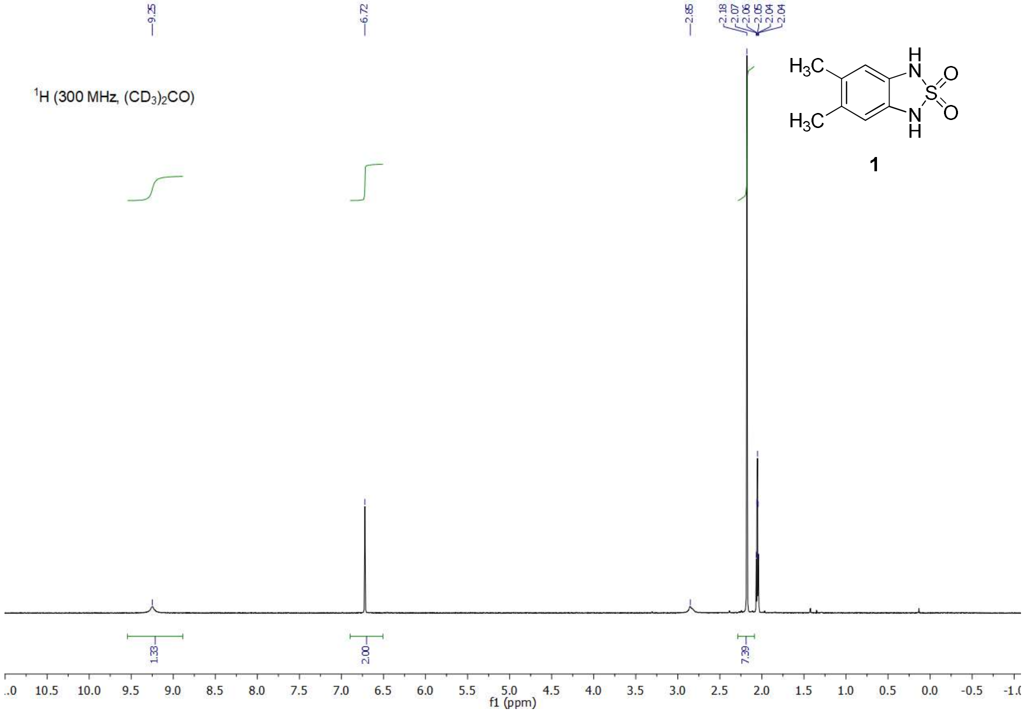

Figure S2. ¹H NMR spectrum of **1**.

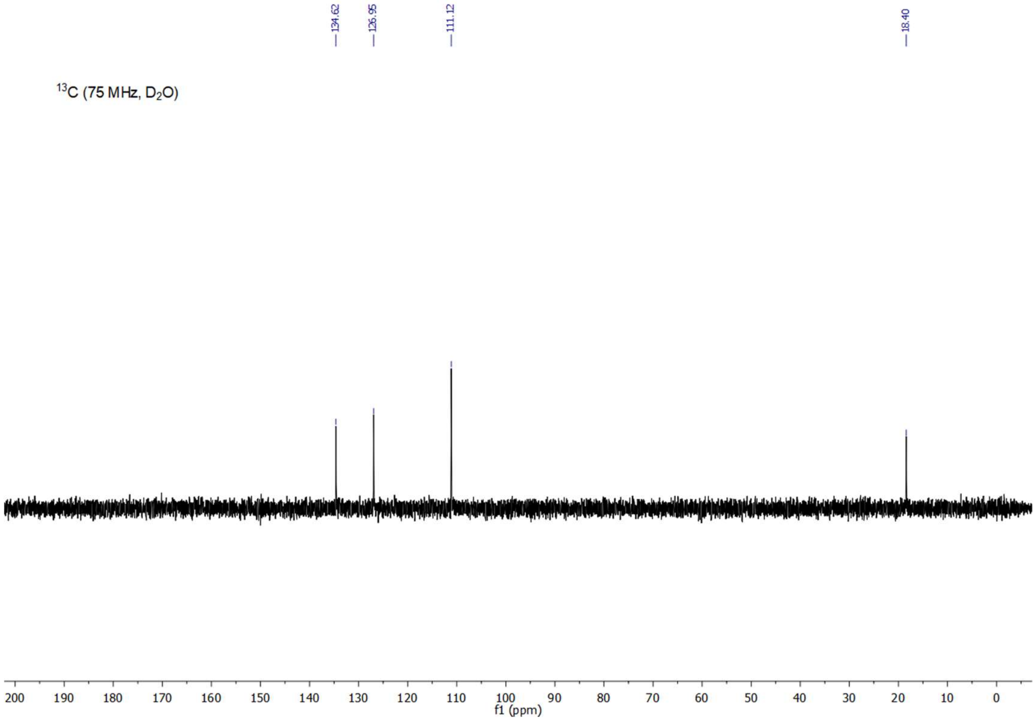

Figure S3. ¹³C NMR spectrum of **1**.

S1.2. 1*H*,3*H*-2,1,3-Benzothiadiazole-2,2-dioxide (1a)

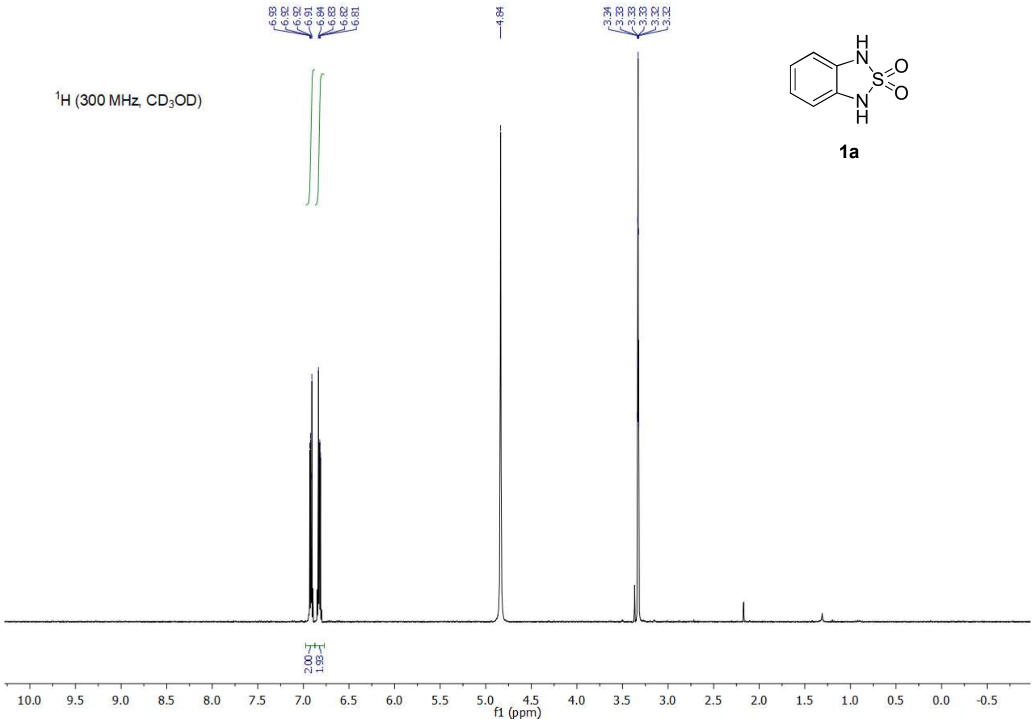

Figure S4. ^{1}H NMR spectrum of **1a**.

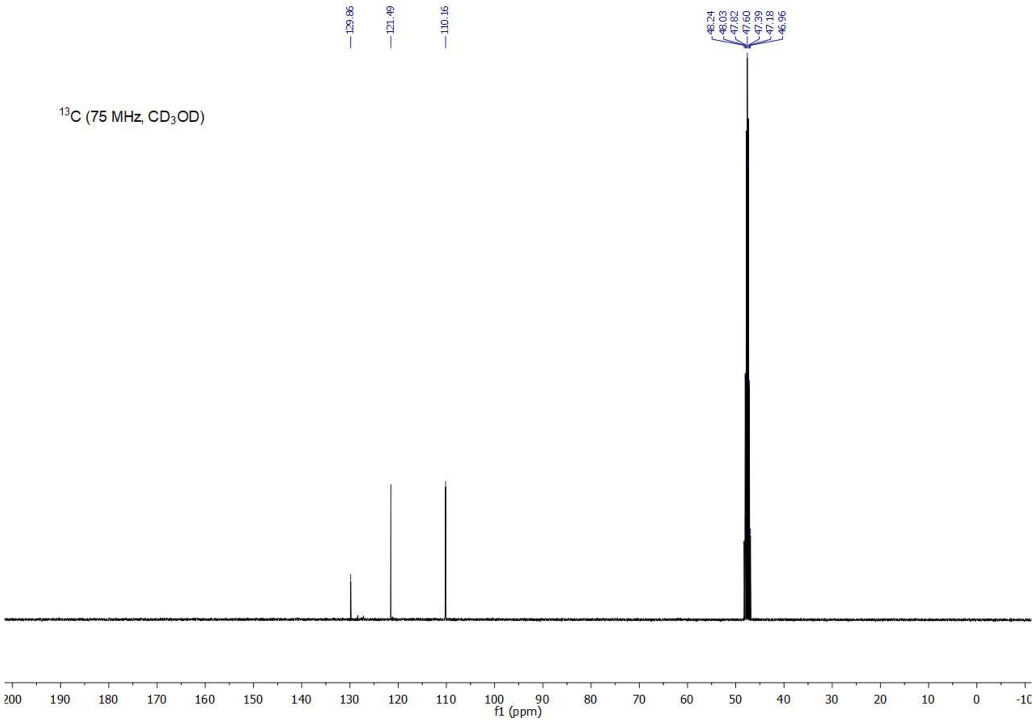

Figure S5. ^{13}C NMR spectrum of **1a**.

S1.3. 5-Methyl-1*H*,3*H*-2,1,3-benzothiadiazole-2,2-dioxide (1b)

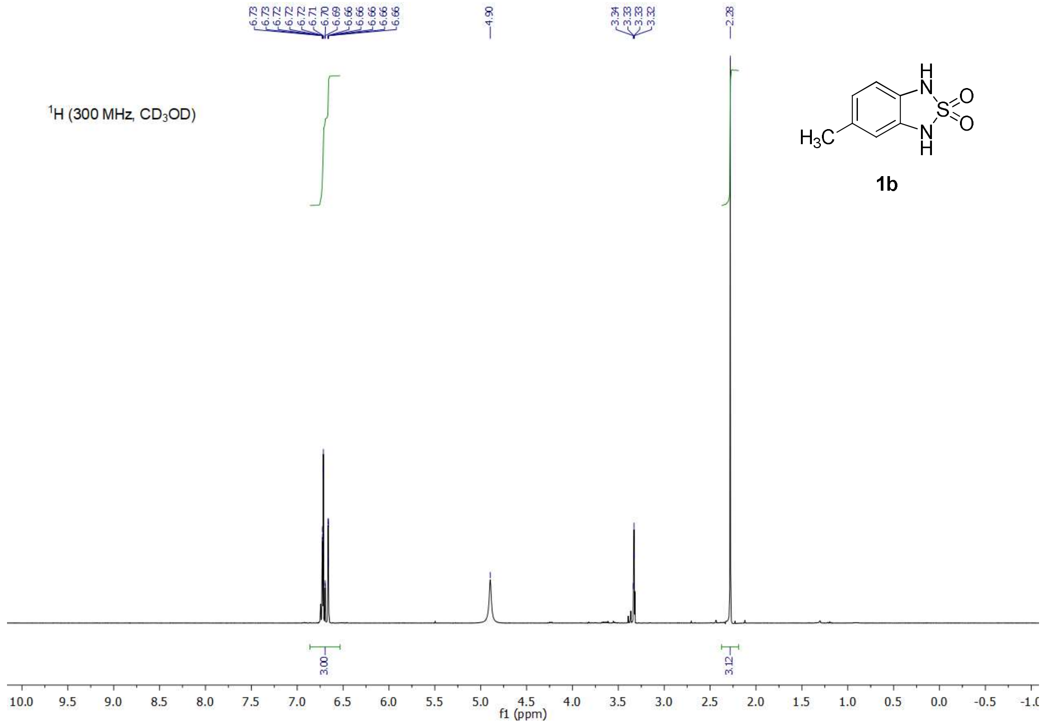

Figure S6. ^{1}H NMR spectrum of **1b**.

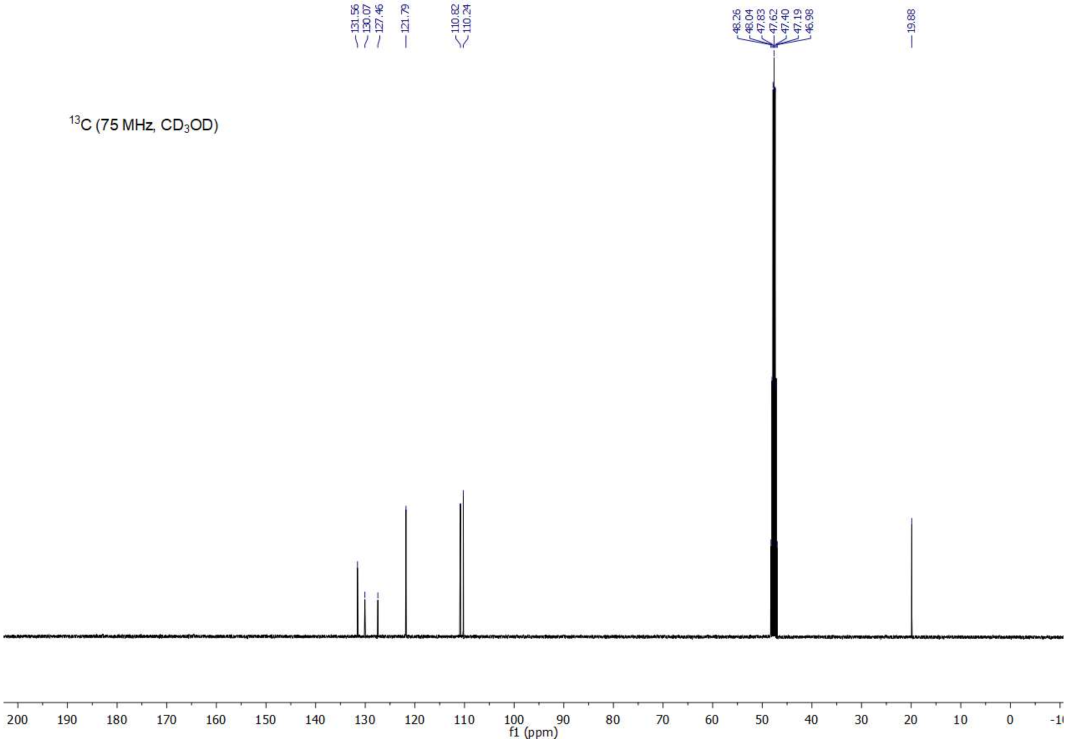

Figure S7. ^{13}C NMR spectrum of **1b**.

S1.4. 5-(Trifluoromethyl)-1*H*,3*H*-2,1,3-benzothiadiazole-2,2-dioxide (1c)

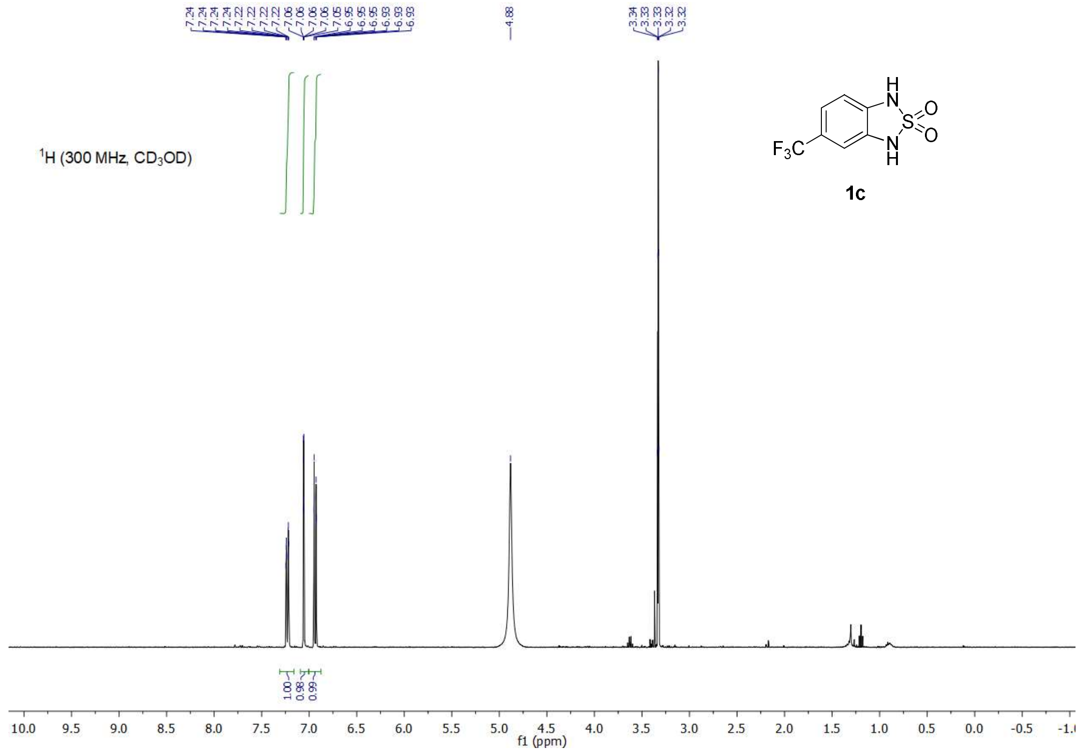

Figure S8. ^{1}H NMR spectrum of **1c**.

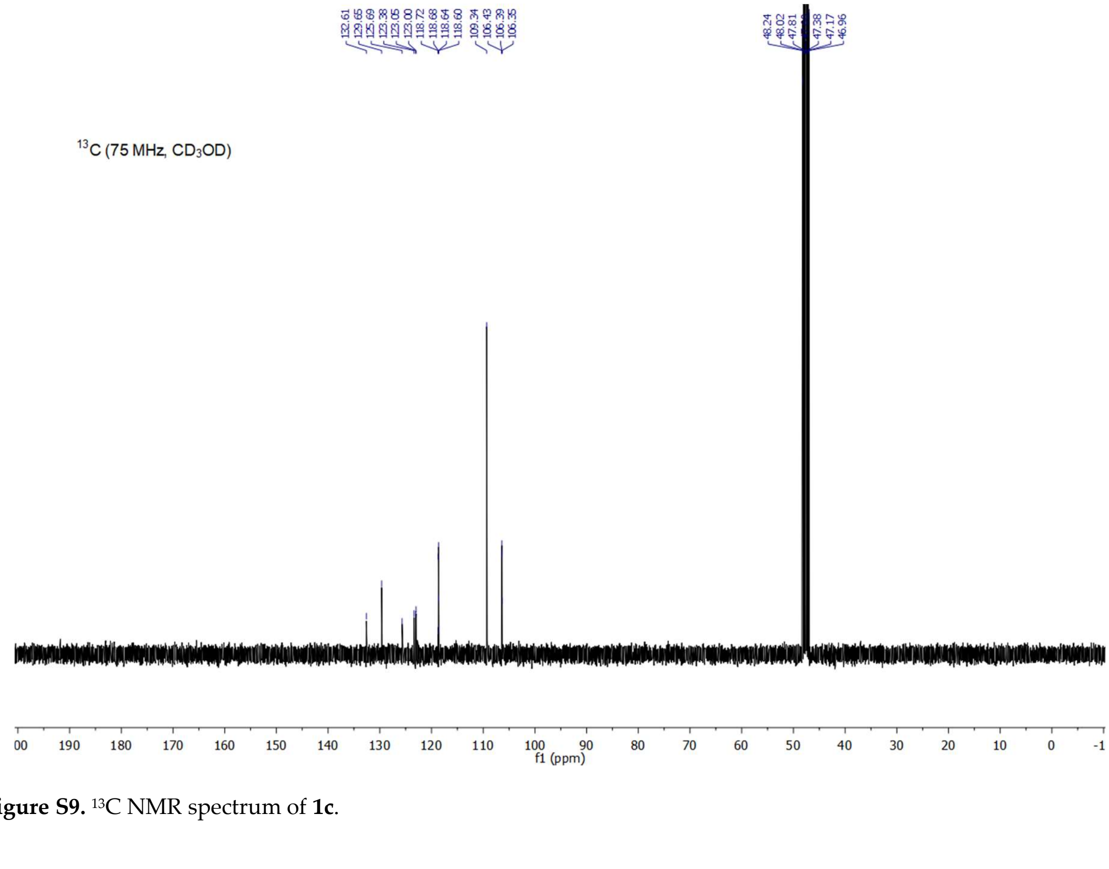

Figure S9. ^{13}C NMR spectrum of **1c**.

S1.5. 5-Nitro-1*H*,3*H*-2,1,3-benzothiadiazole-2,2-dioxide (1d)

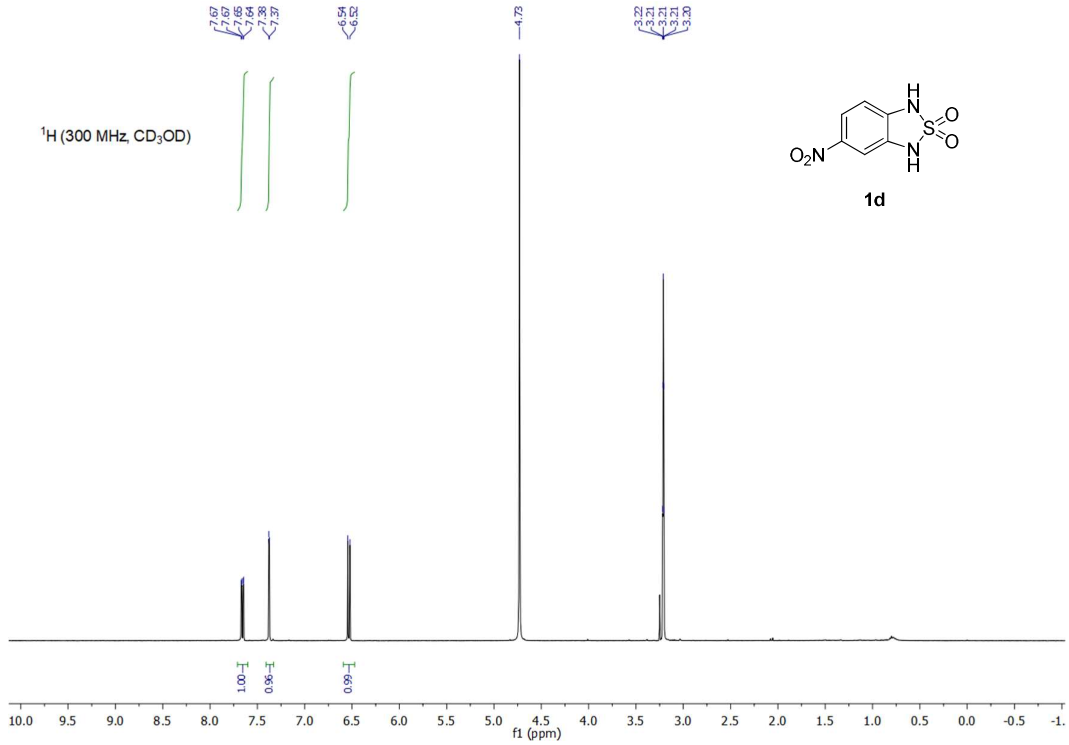

Figure S10. ^{1}H NMR spectrum of **1d**.

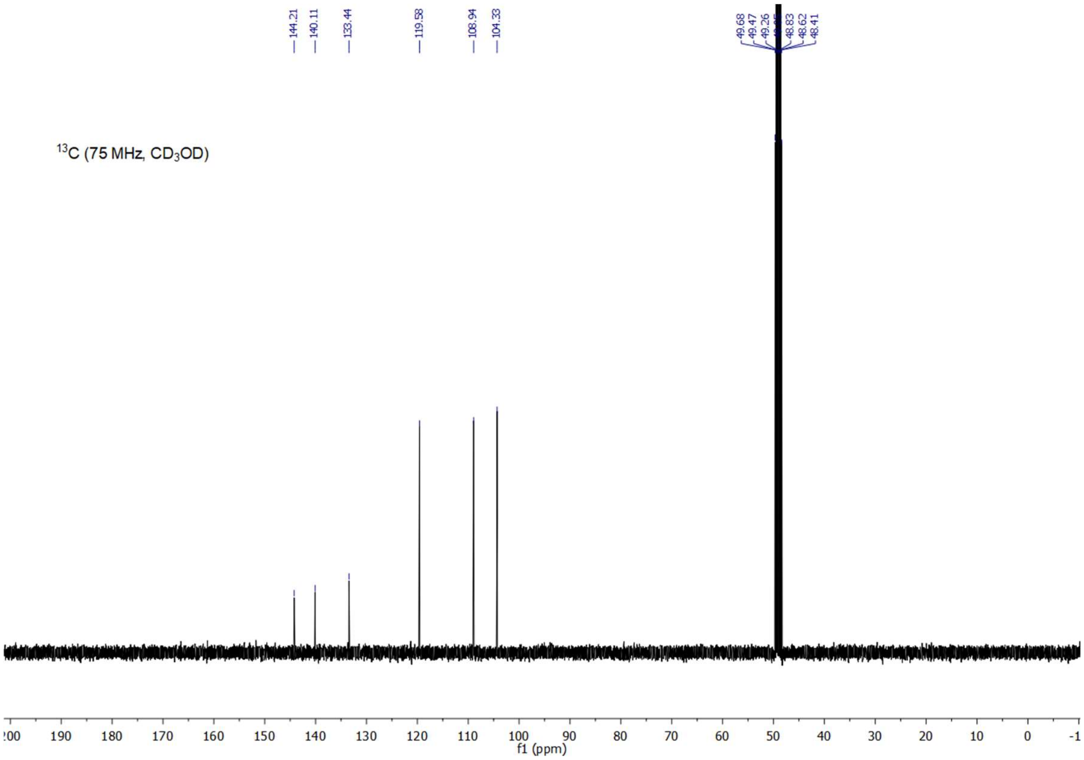

Figure S11. ^{13}C NMR spectrum of **1d**.

S1.6. 5-Chloro-*N*-[(2,4-dimethoxyphenyl)methyl]-2-nitroaniline (7)

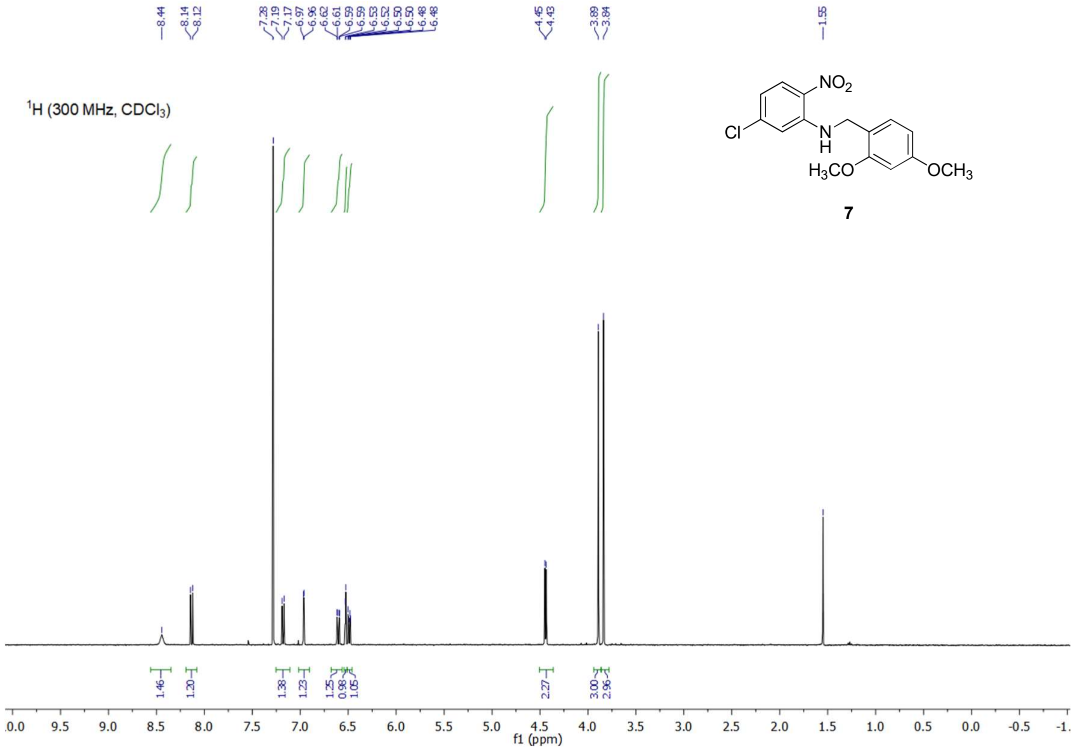

Figure S12. ^{1}H NMR spectrum of **7**.

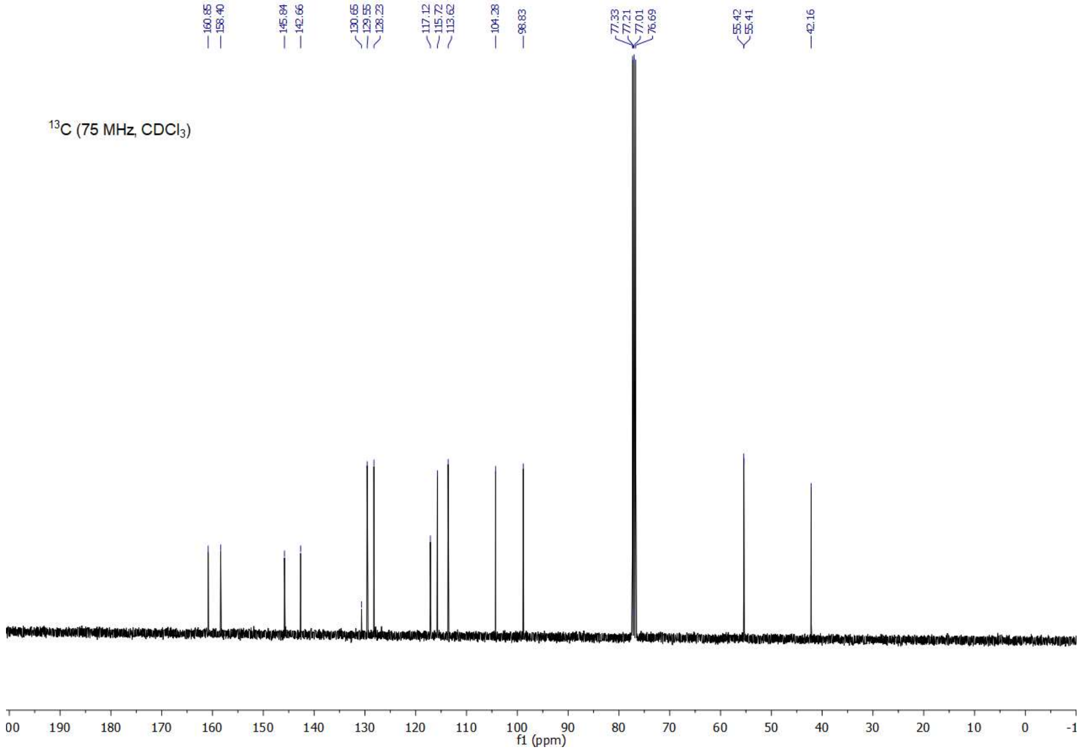

Figure S13. ^{13}C NMR spectrum of **7**.

S1.7. 5-Bromo-*N*-[(4-methoxyphenyl)methyl]-4-methyl-2-nitroaniline (8)

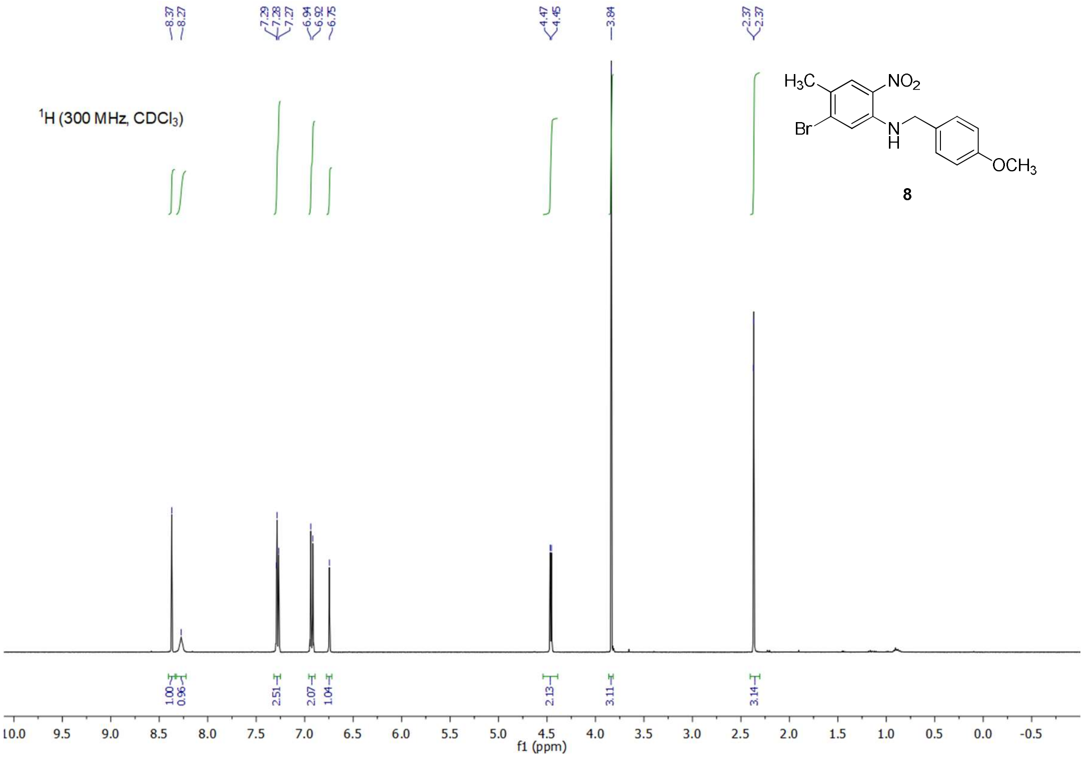

Figure S14. ^{1}H NMR spectrum of **8**.

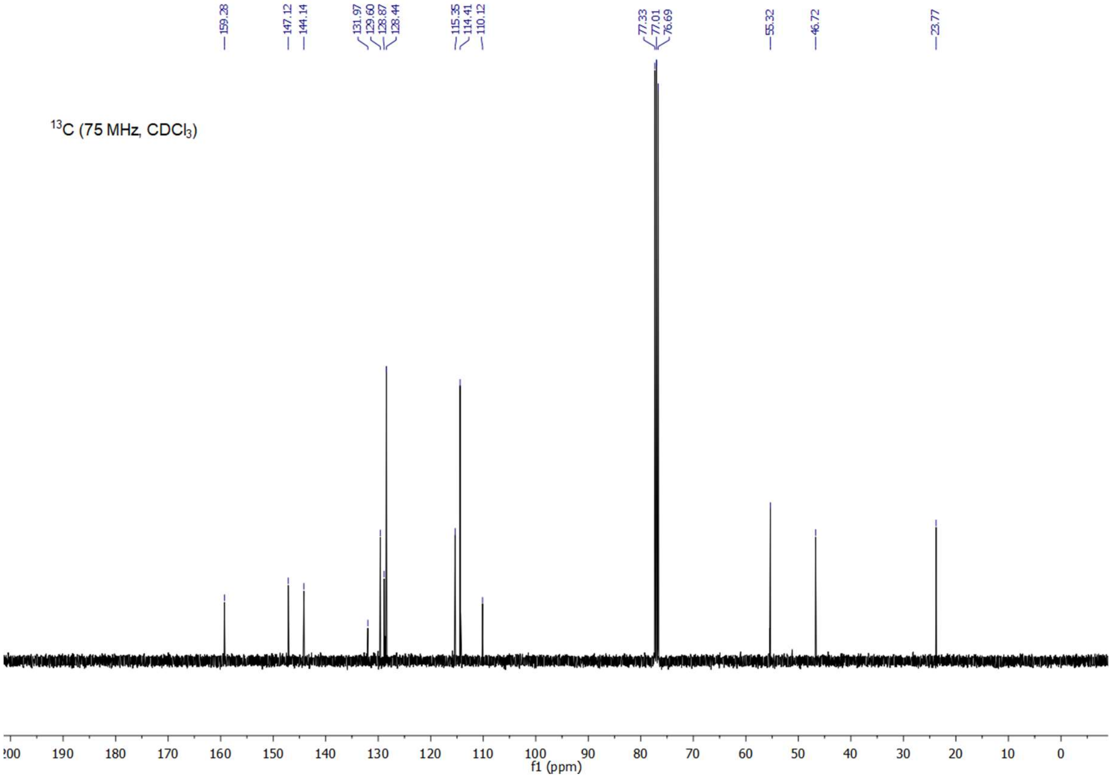

Figure S15. ^{13}C NMR spectrum of **8**.

S1.8. 5-Chloro-*N*1-[(2,4-dimethoxyphenyl)methyl]benzene-1,2-diamine (9)

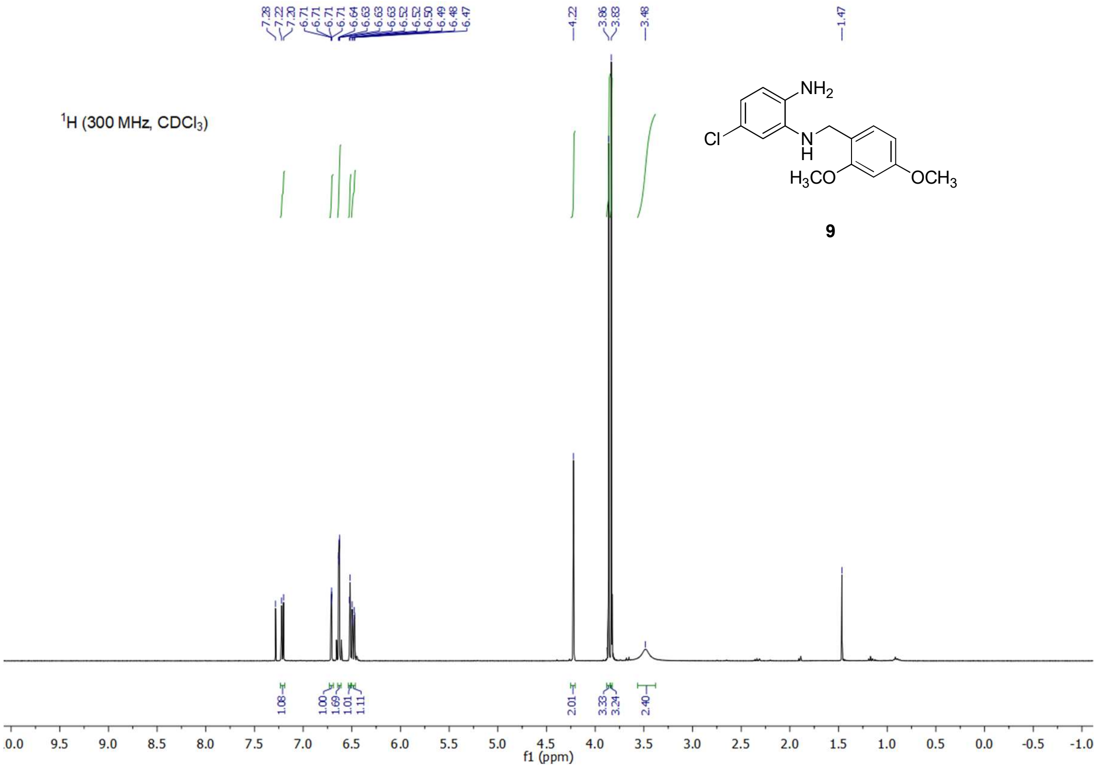

Figure S16. ^{1}H NMR spectrum of **9**.

S1.9. 5-Bromo-*N*1-[(4-methoxyphenyl)methyl]-4-methylbenzene-1,2-diamine (10)

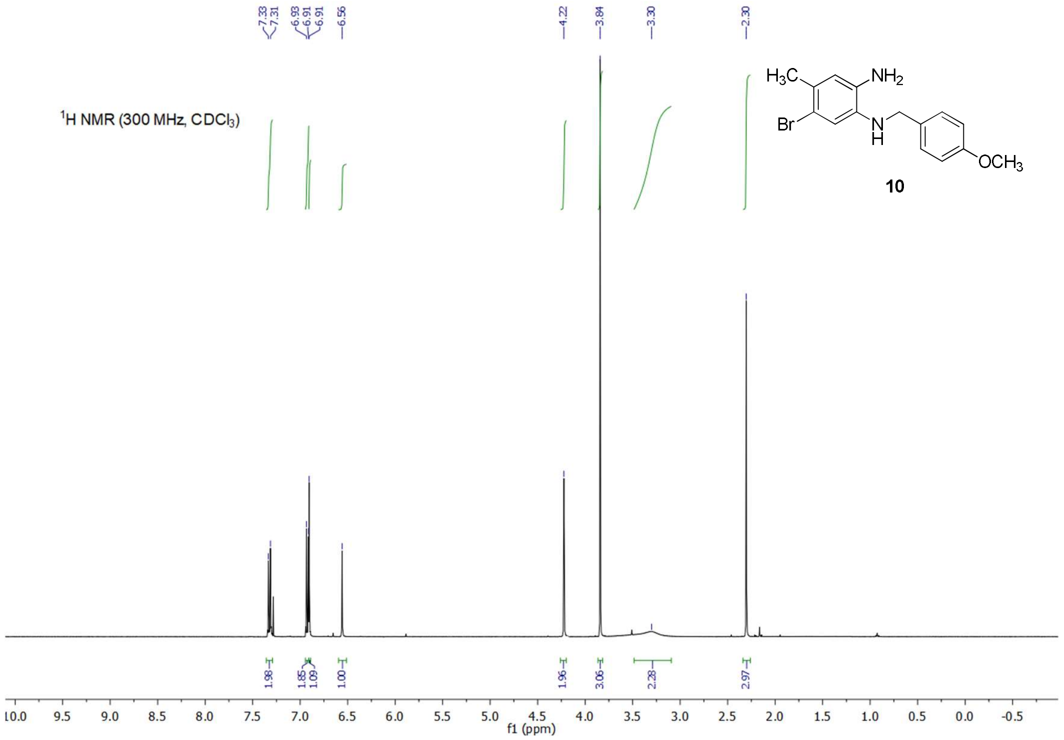

Figure S17. ^{1}H NMR spectrum of **10**.

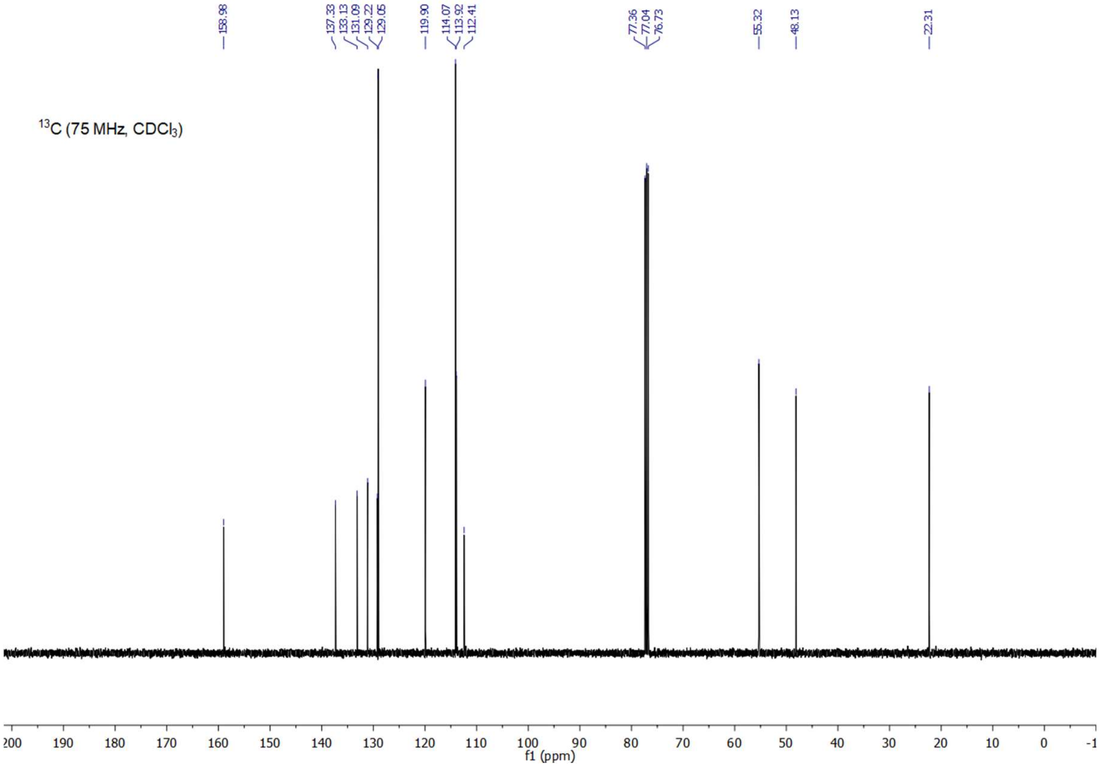

Figure S18. ^{13}C NMR spectrum of **10**.

S1.10. 6-Chloro-1-[(2,4-dimethoxyphenyl)methyl]-1*H*,3*H*-2,1,3-benzothiadiazole-2,2-dioxide (11)

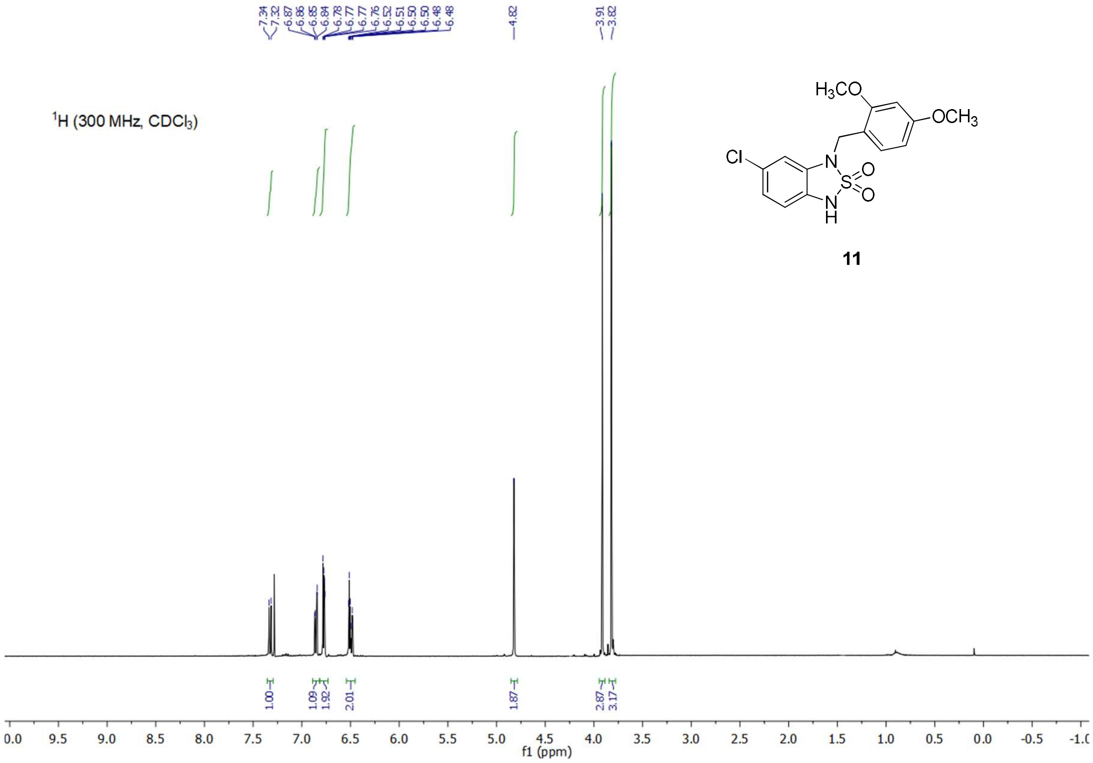

Figure S19. ^{1}H NMR spectrum of **11**.

S1.11. 6-Bromo-1-[(4-methoxyphenyl)methyl]-5-methyl-1*H*,3*H*-2,1,3-benzothiadiazole-2,2-dioxide (12)

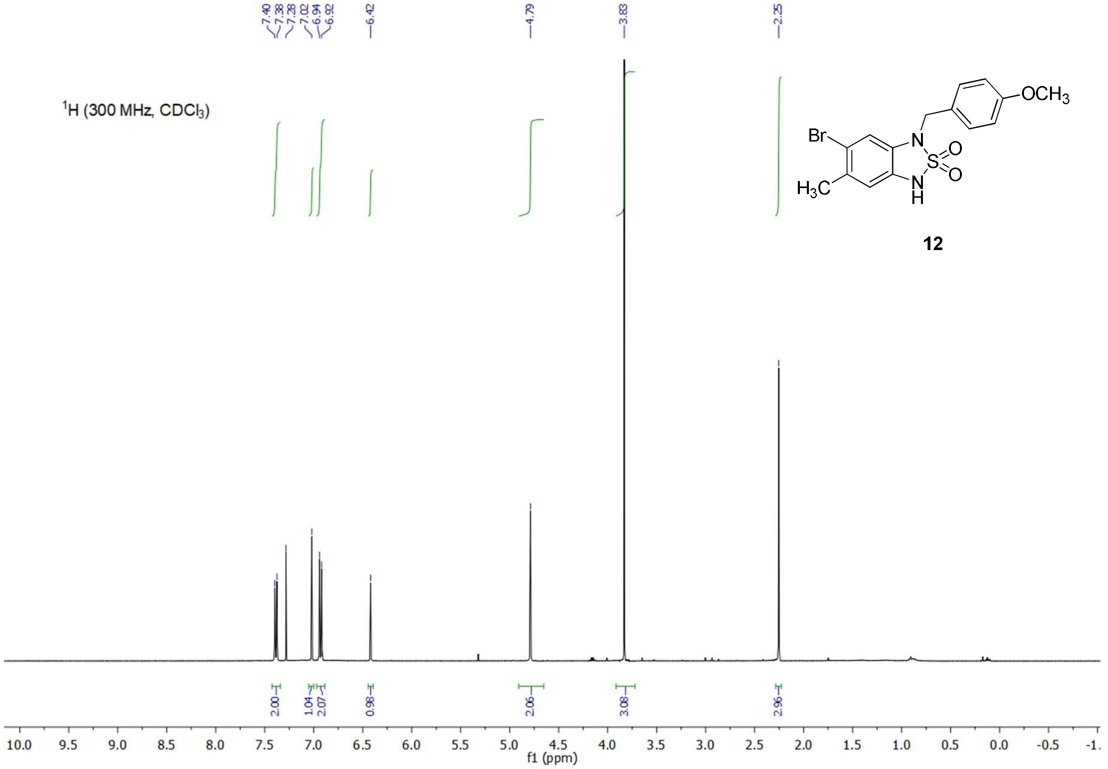

Figure S20. ^{1}H NMR spectrum of **12**.

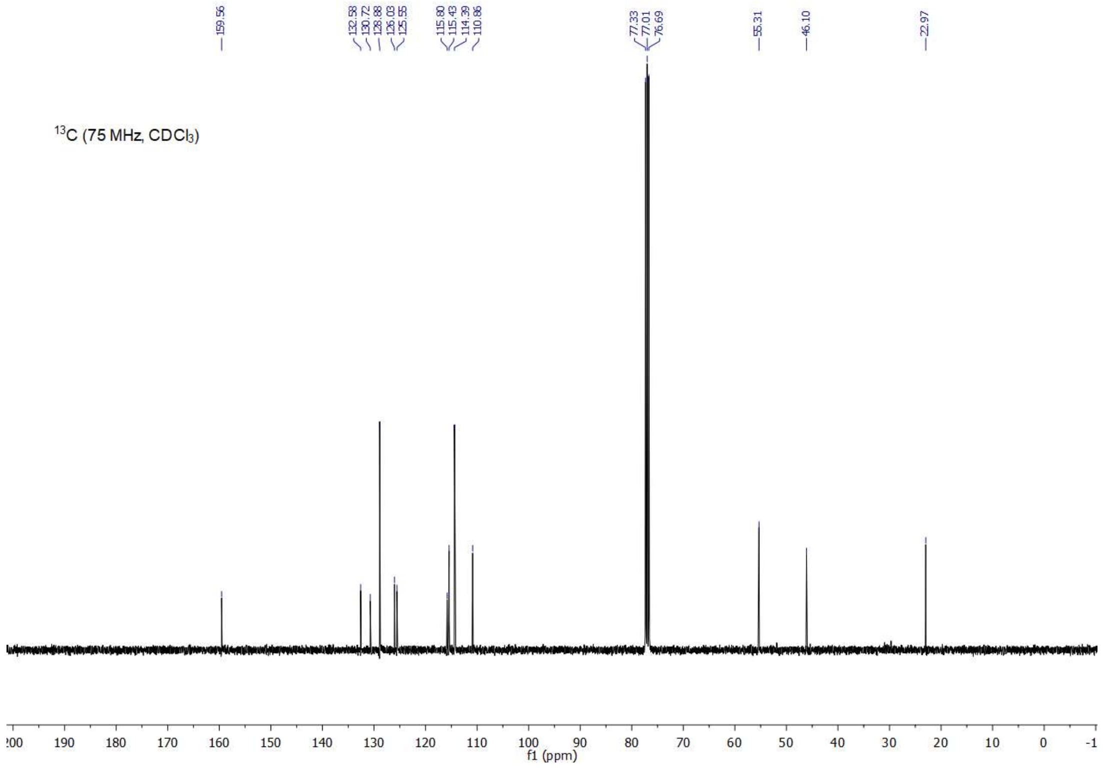

Figure S21. ^{13}C NMR spectrum of **12**.

S1.12. 5-Chloro-1*H*,3*H*-2,1,3-benzothiadiazole-2,2-dioxide (1e)

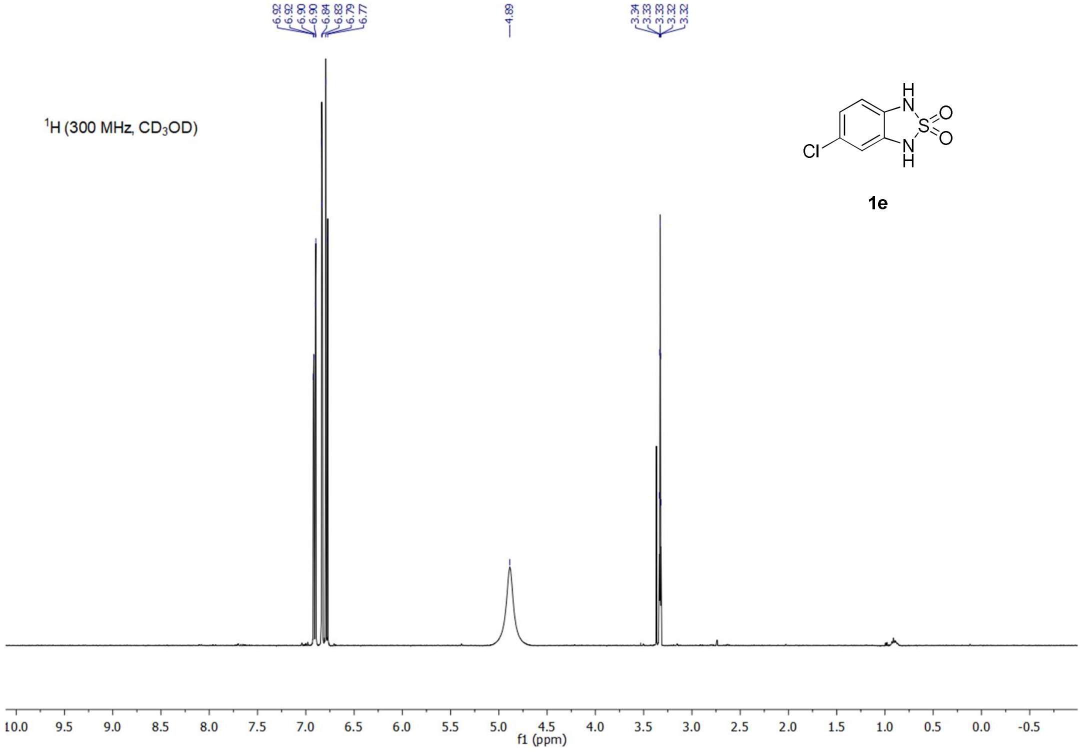

Figure S22. ^{1}H NMR spectrum of **1e**.

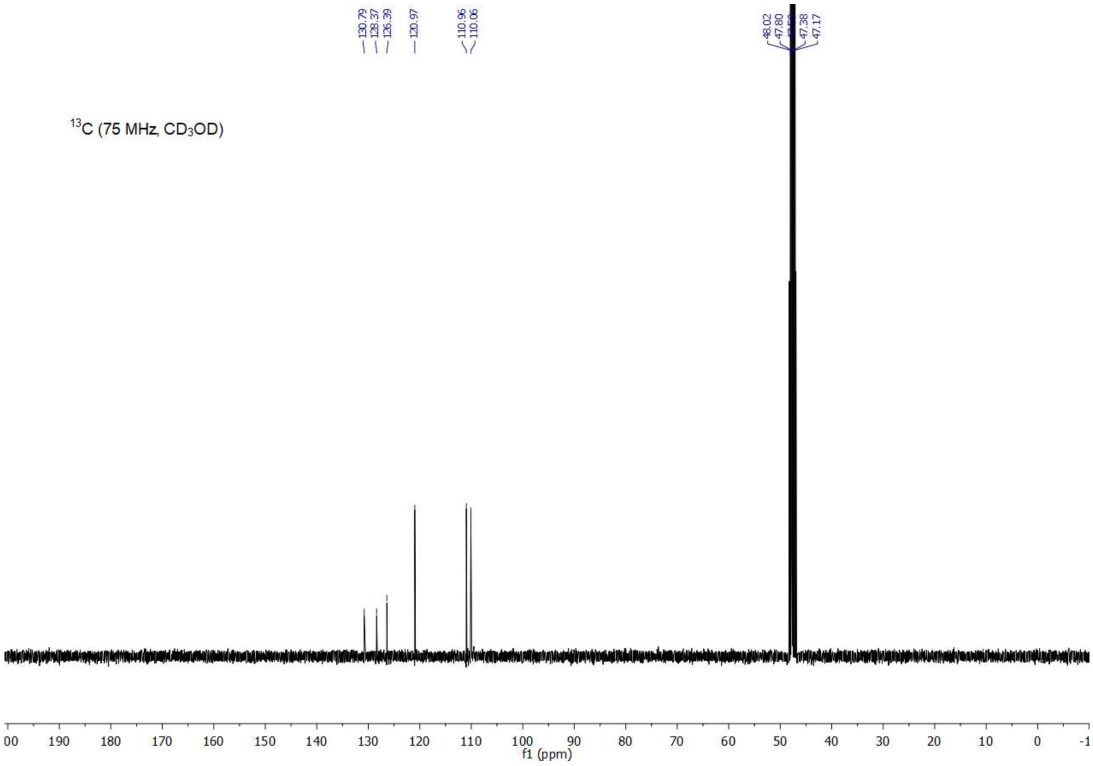

Figure S23. ^{13}C NMR spectrum of **1e**.

S1.13. 5-Bromo-6-methyl-1*H*,3*H*-2,1,3-benzothiadiazole-2,2-dioxide (2a)

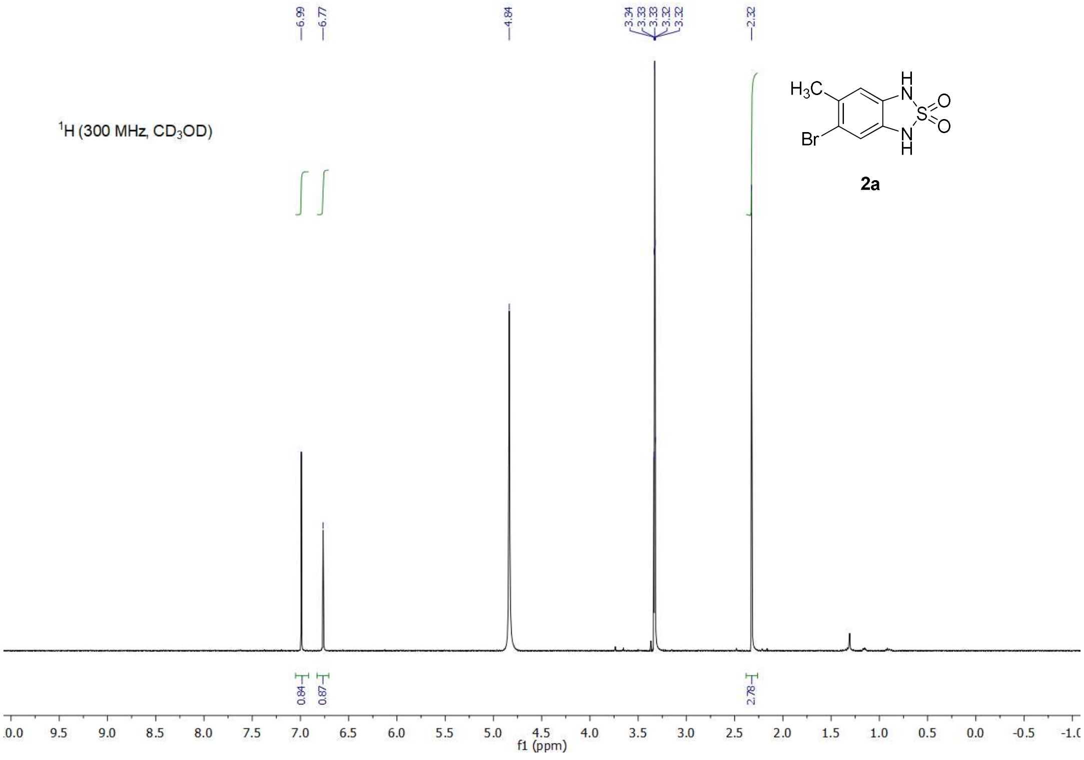

Figure S24. ^{1}H NMR spectrum of **2a**.

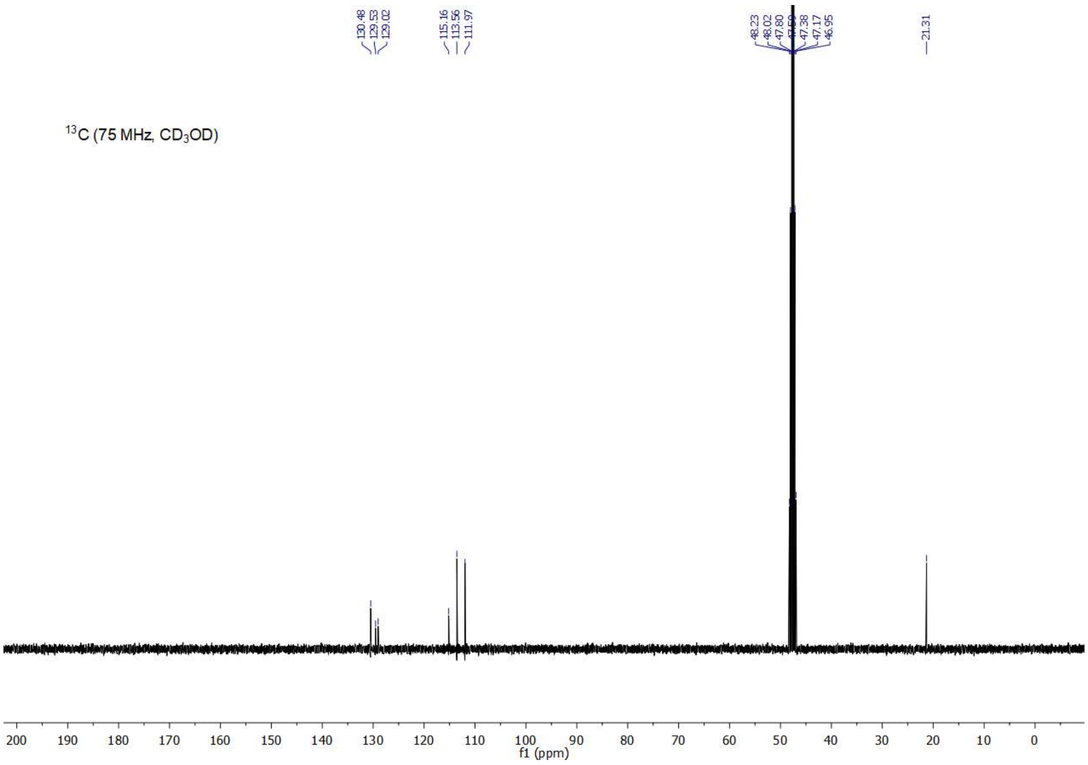

Figure S25. ^{13}C NMR spectrum of **2a**.

S1.14. 1,3-Bis[(4-methoxyphenyl)methyl]-5-nitro-2,1,3-benzothiadiazole-2,2-dioxide (13)

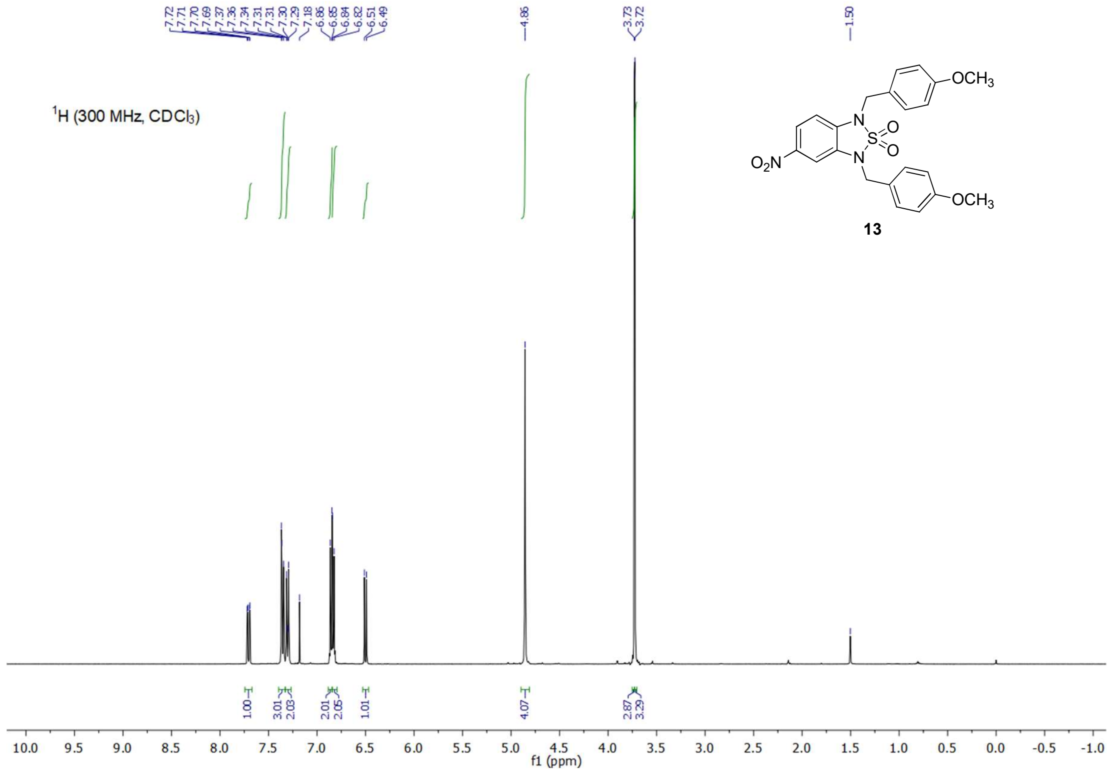

Figure S26. ^{1}H NMR spectrum of **13**.

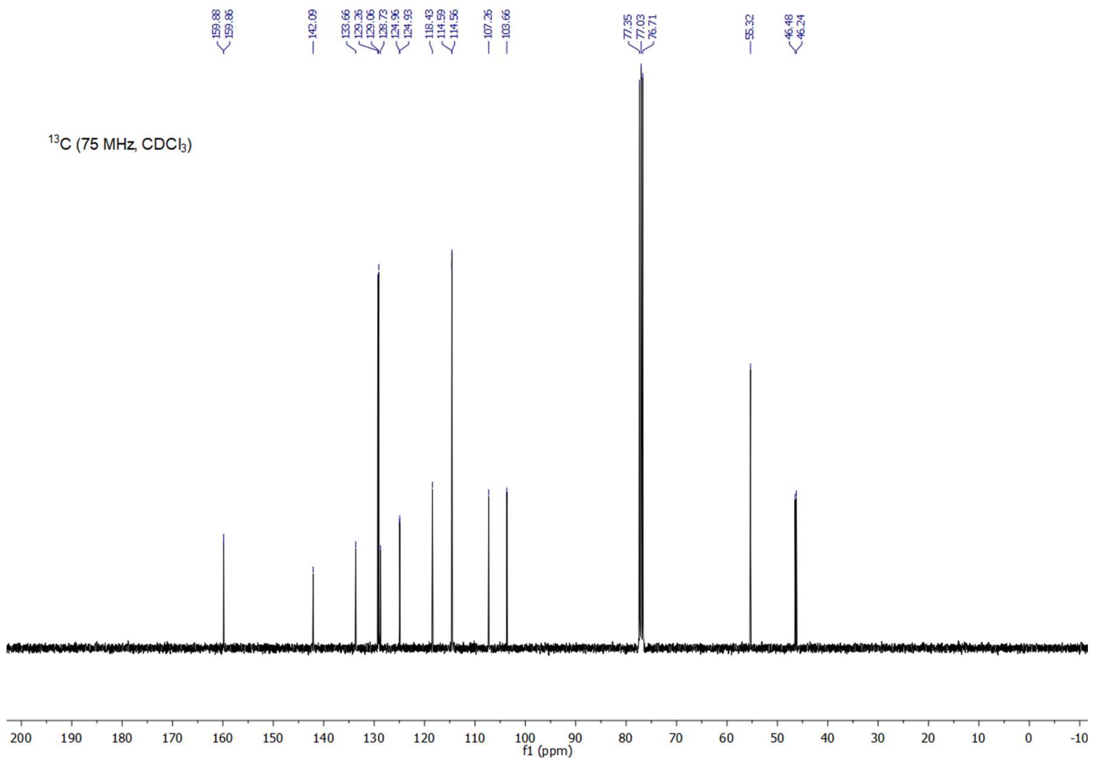

Figure S27. ^{13}C NMR spectrum of **13**.

S1.15. 5-Amino-1,3-bis[(4-methoxyphenyl)methyl]-2,1,3-benzothiadiazole-2,2-dioxide (14)

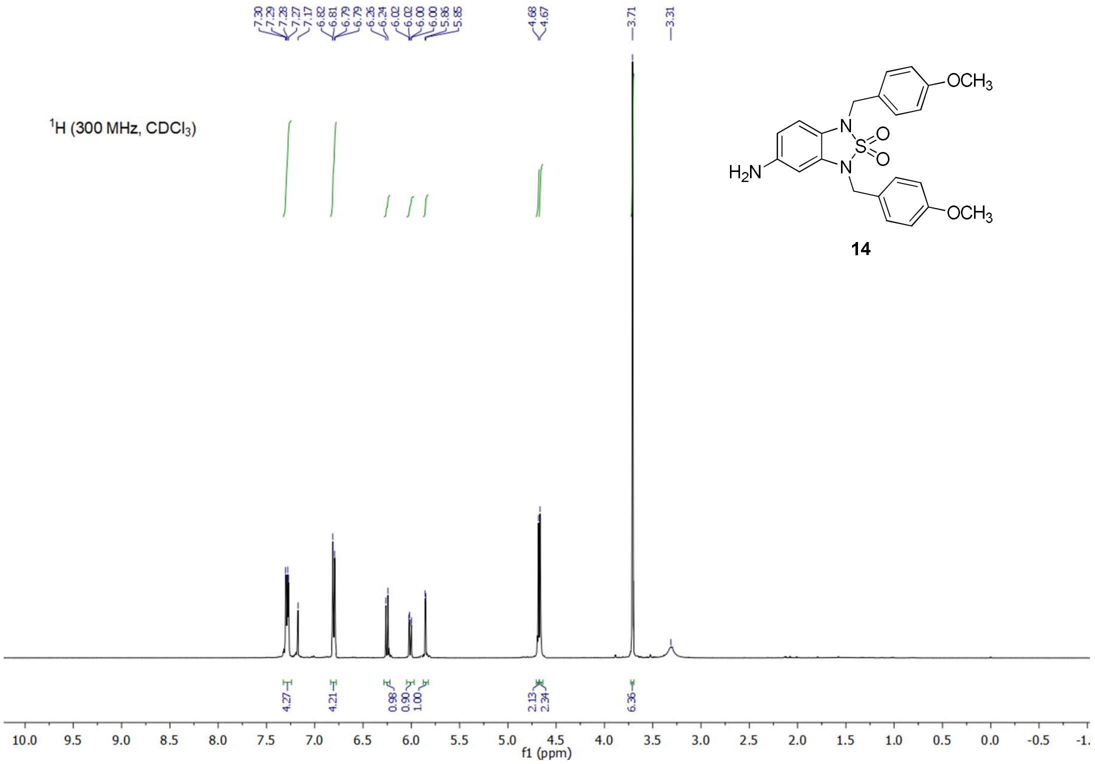

Figure S28. ^{1}H NMR spectrum of **14**.

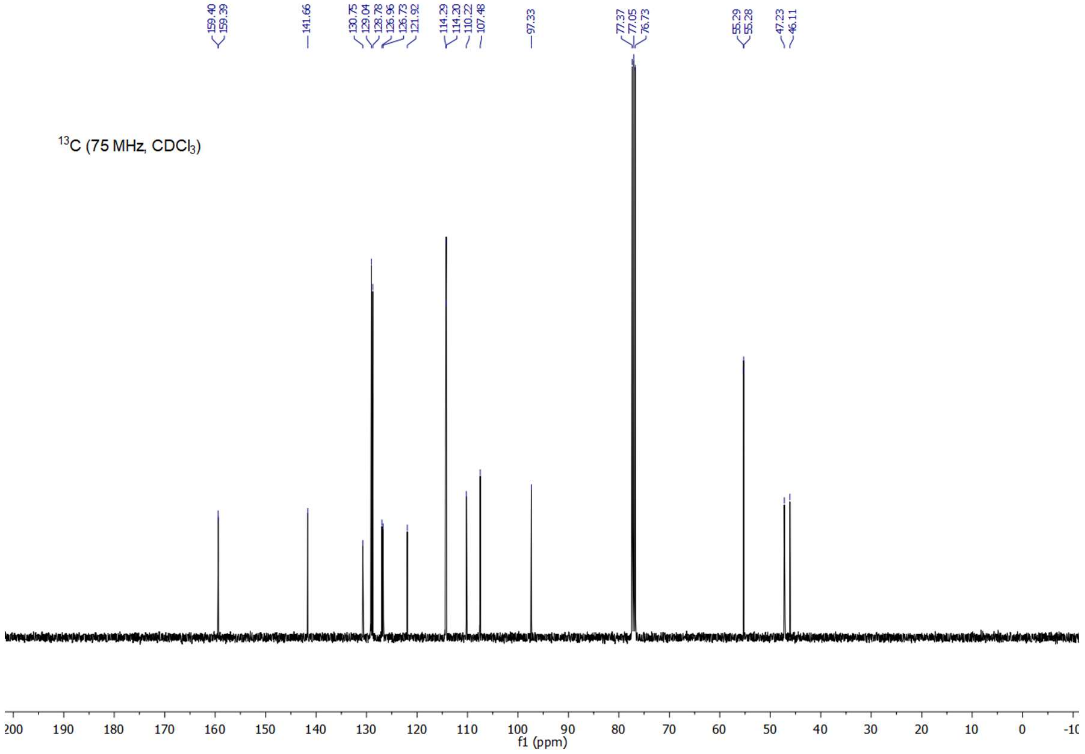

Figure S29. ^{13}C NMR spectrum of **14**.

S1.16. *N*-{1,3-Bis[(4-methoxyphenyl)methyl]-2,2-dioxo-2,1,3-benzothiadiazol-5-yl}acetamide (15)

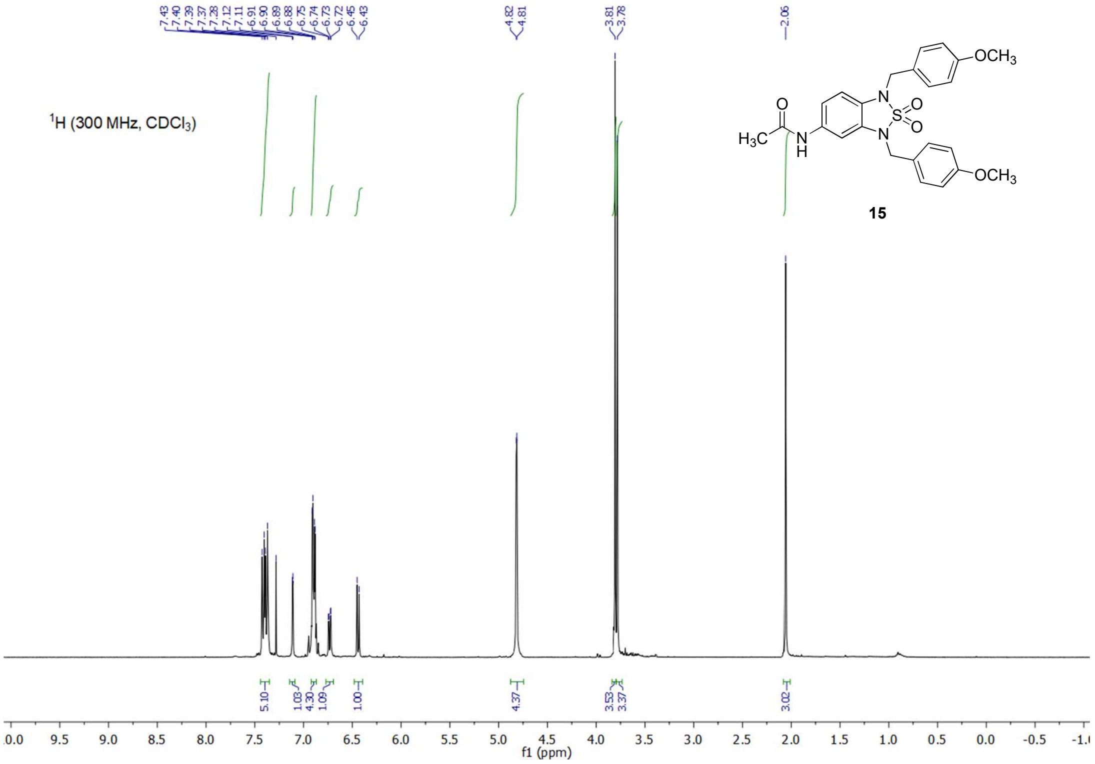

Figure S30. ^{1}H NMR spectrum of **15**.

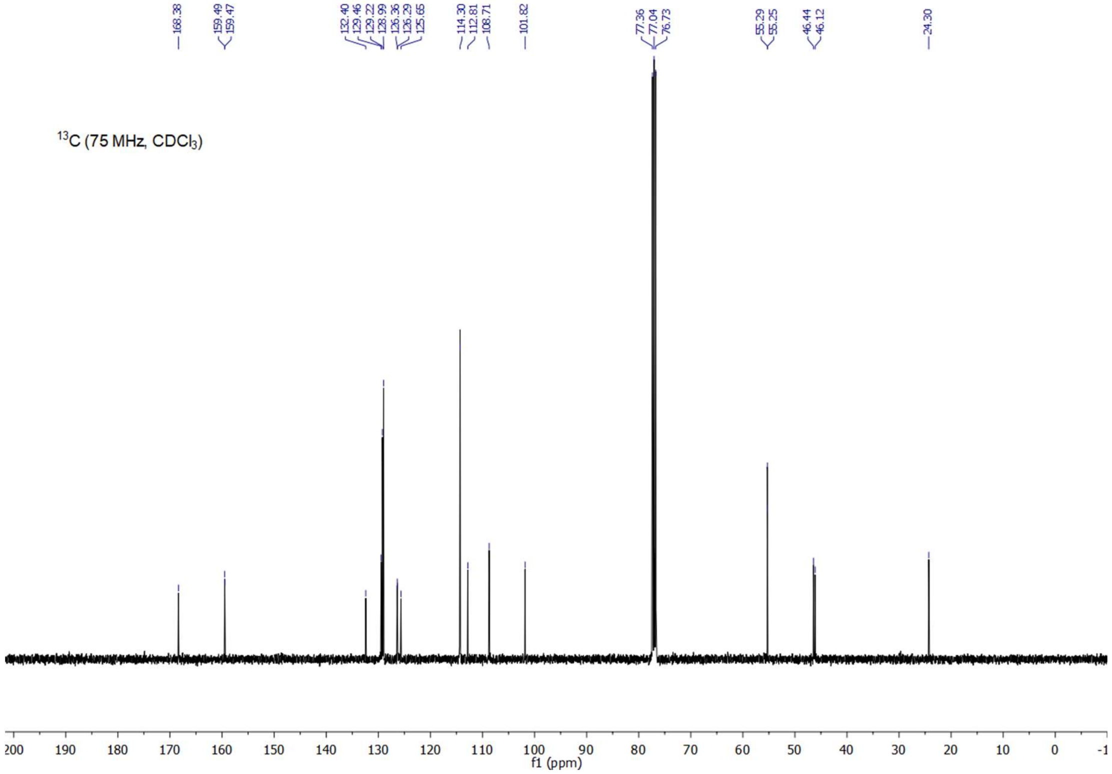

Figure S31. ^{13}C NMR spectrum of **15**.

S1.17. *N*-(2,2-Dioxo-1*H*,3*H*-2,1,3-benzothiadiazol-5-yl)acetamide (1f)

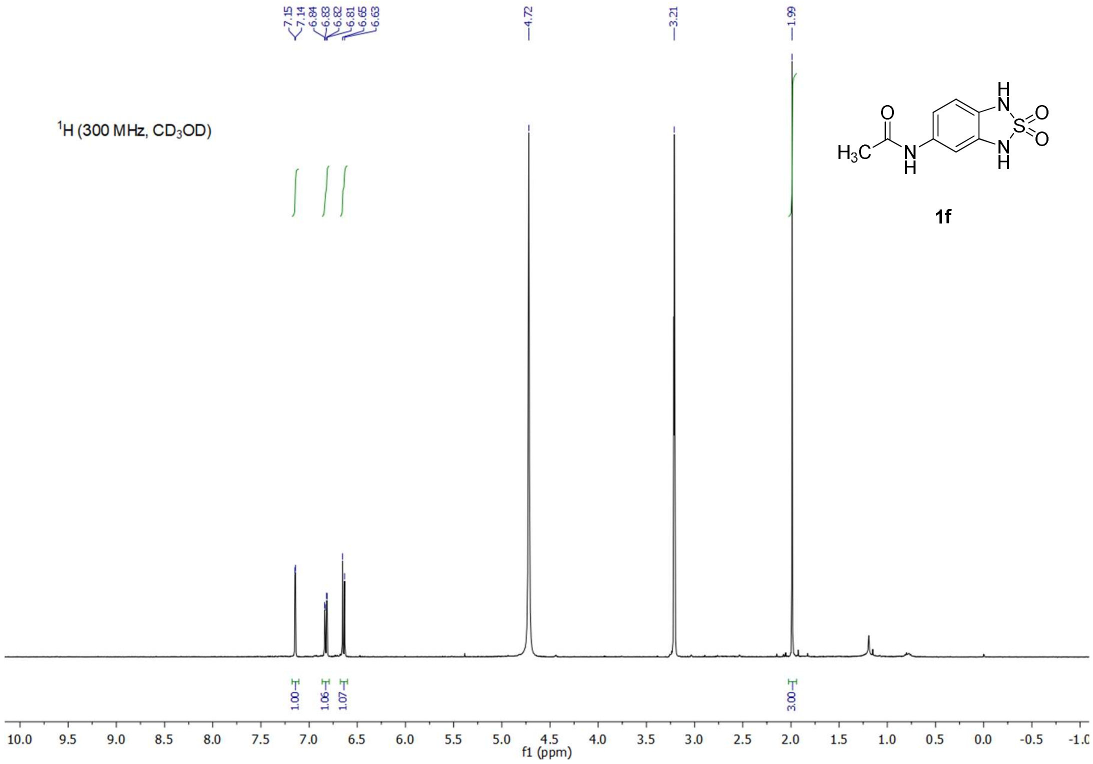

Figure S32. ^{1}H NMR spectrum of **1f**.

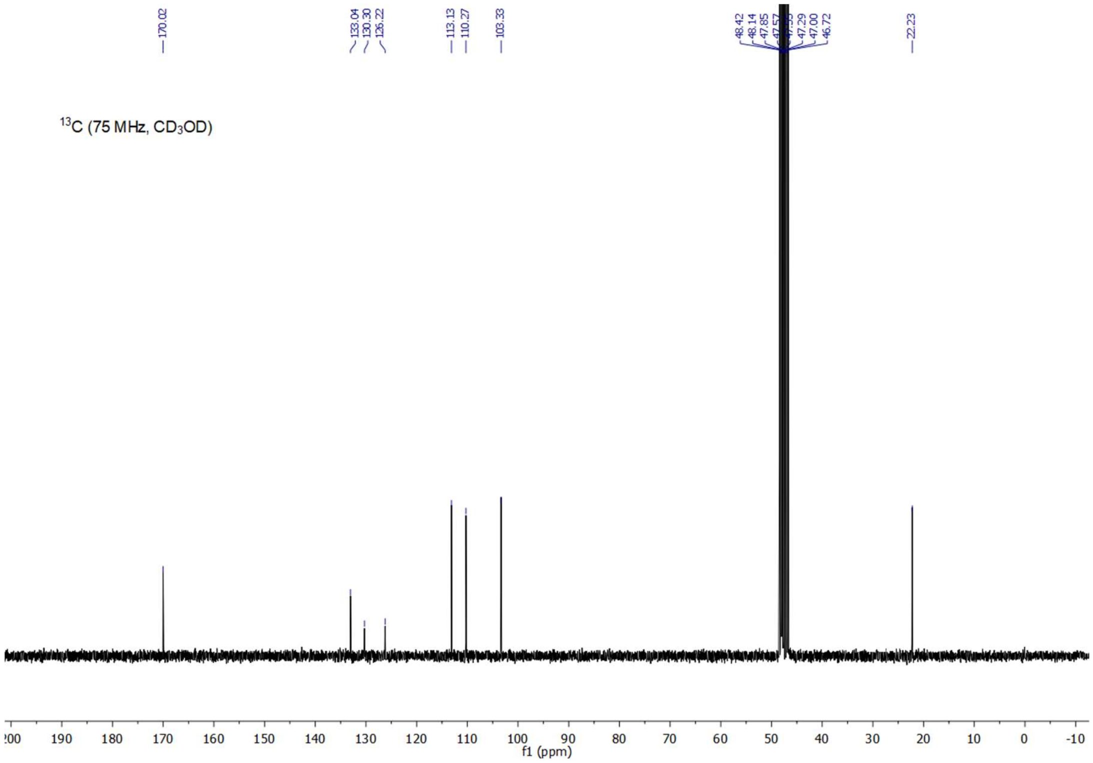

Figure S33. ^{13}C NMR spectrum of **1f**.

S1.18. 4-Bromo-*N*-[(4-methoxyphenyl)methyl]-2-nitroaniline (16)

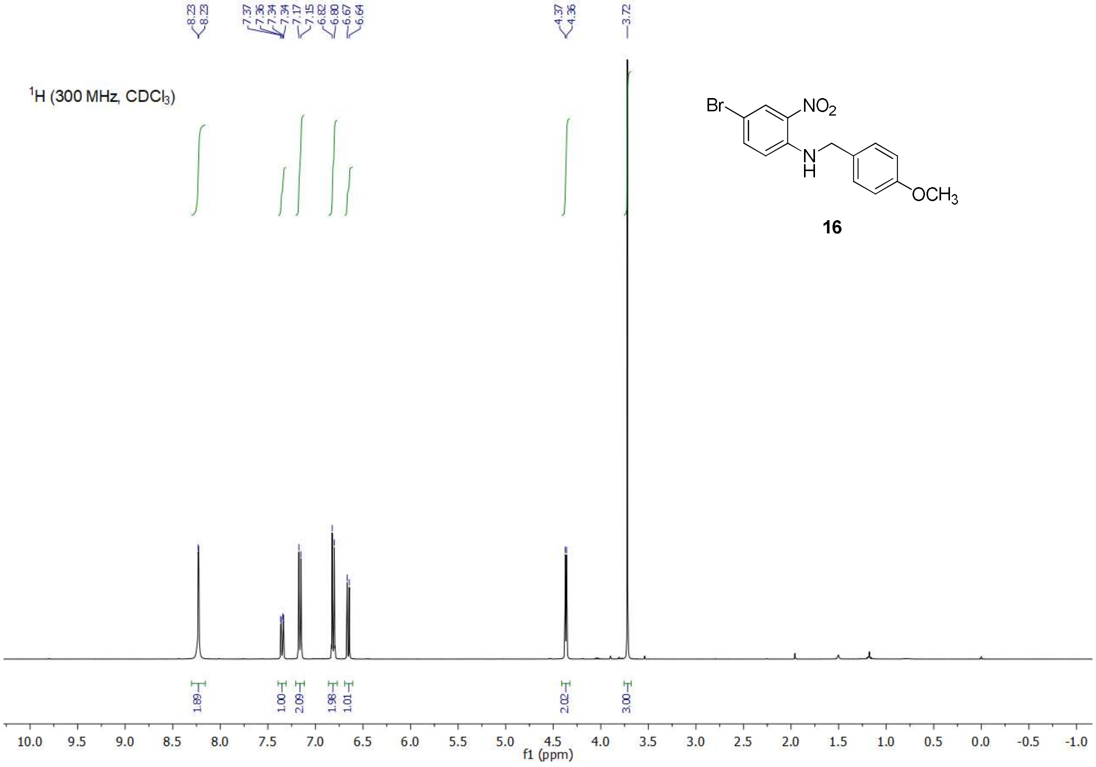

Figure S34. ^{1}H NMR spectrum of **16**.

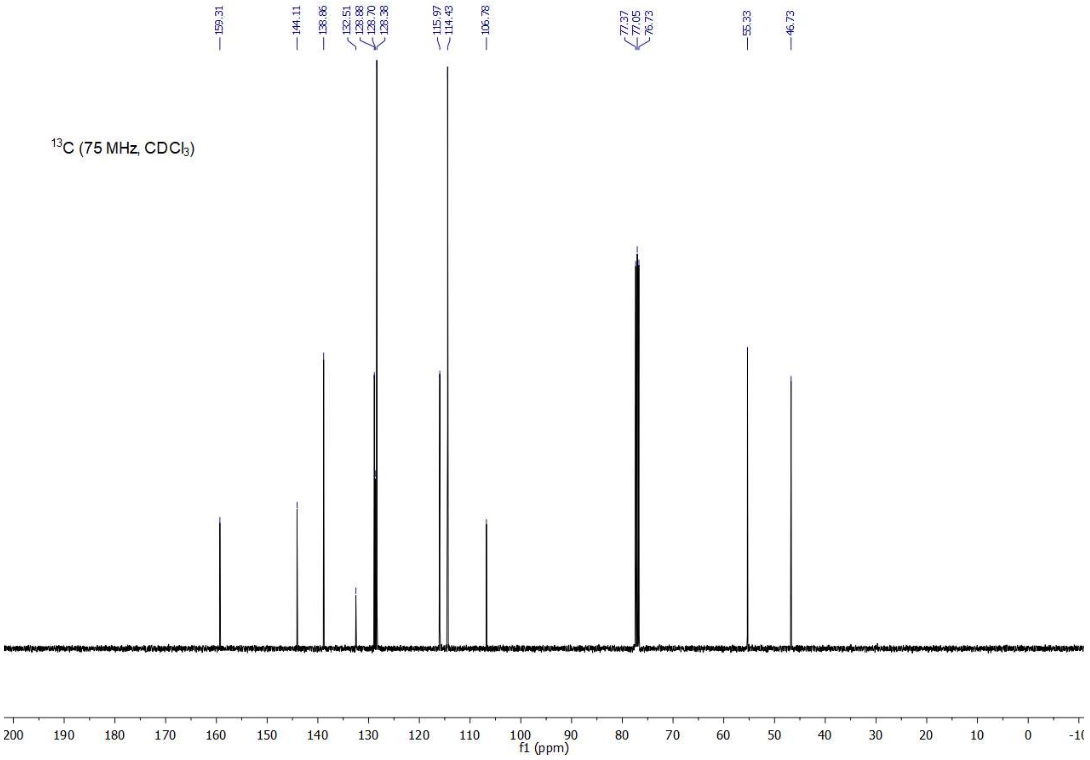

Figure S35. ^{13}C NMR spectrum of **16**.

S1.19. 4-Bromo-*N*1-[(4-methoxyphenyl)methyl]benzene-1,2-diamine (17)

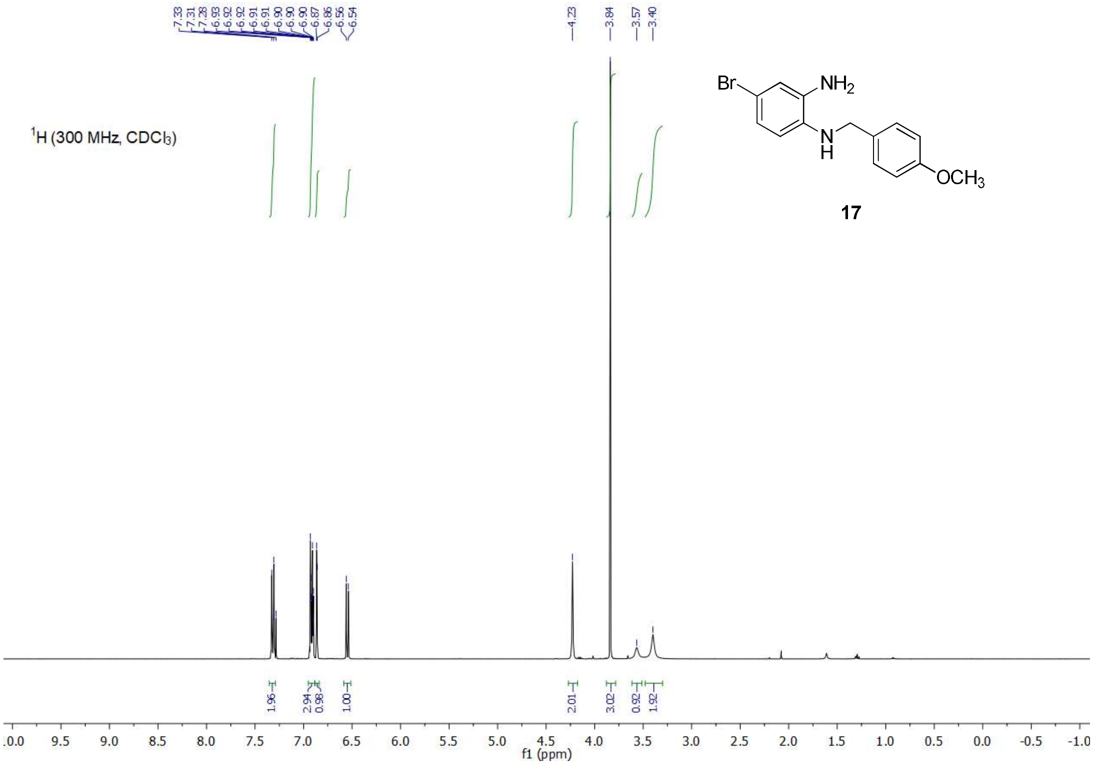

Figure S36. ^{1}H NMR spectrum of **17**.

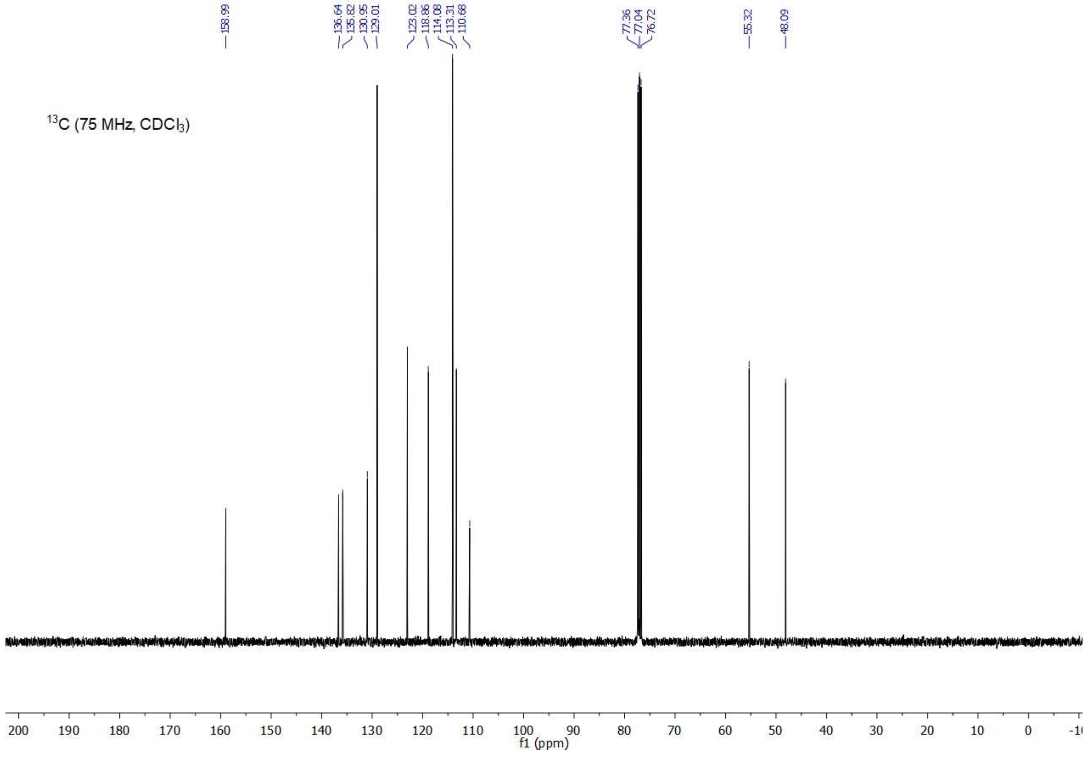

Figure S37. ^{13}C NMR spectrum of **17**.

S1.20. **5-Bromo-1-[(4-methoxyphenyl)methyl]-3*H*-2,1,3-benzothiadiazole-2,2-dioxide (18)**

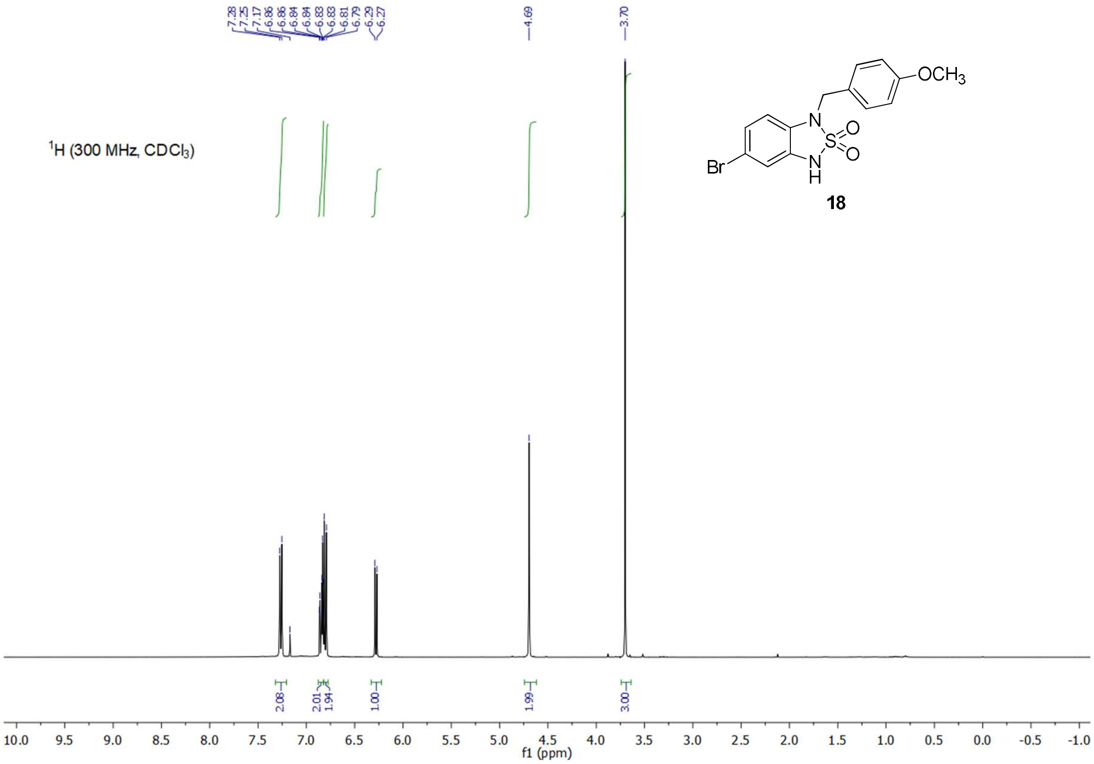

Figure S38. ^{1}H NMR spectrum of **18**.

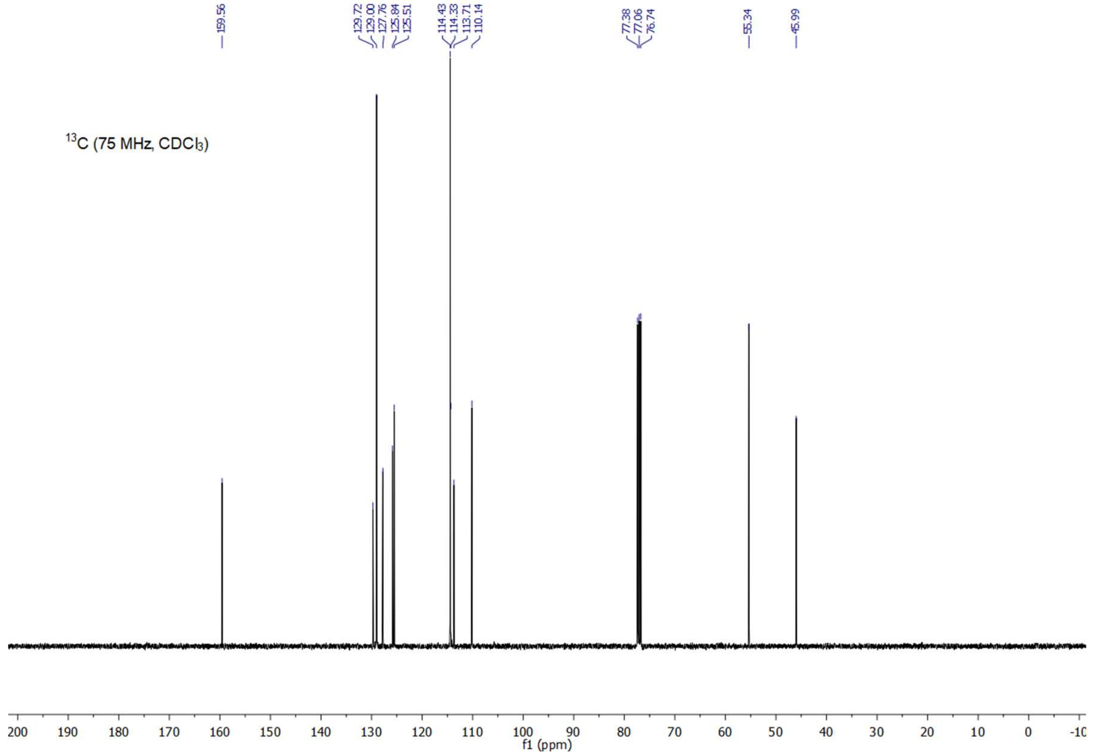

Figure S39. ^{13}C NMR spectrum of **18**.

S1.21. 5-Bromo-1,3-bis[(4-methoxyphenyl)methyl]-2,1,3-benzothiadiazole-2,2-dioxide (19)

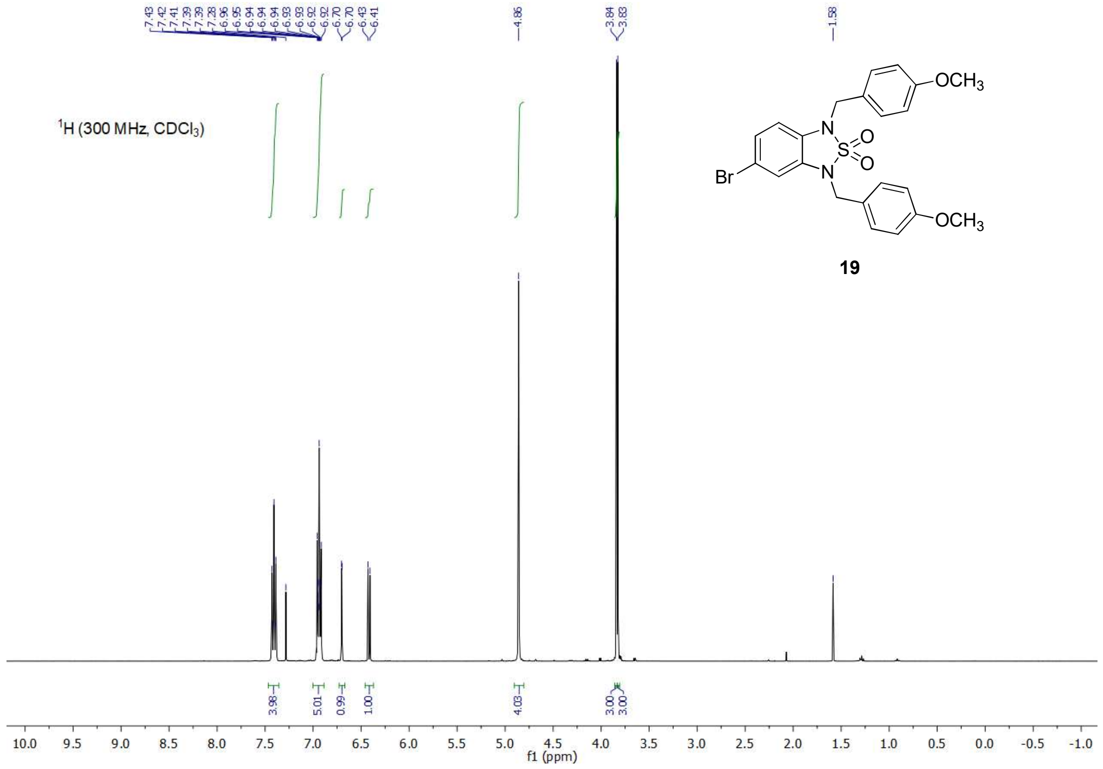

Figure S40. ^{1}H NMR spectrum of **19**.

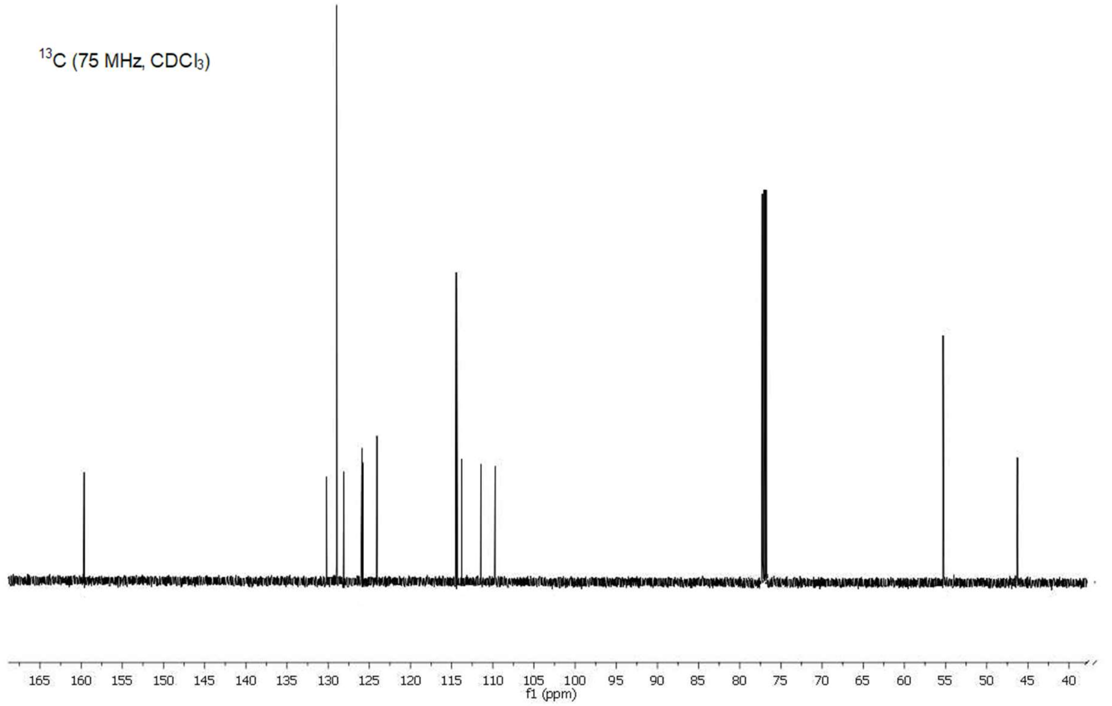

Figure S41. ^{13}C NMR spectrum of **19**.

S1.22. Ethyl(2*E*)-3-{1,3-bis[(4-methoxyphenyl)methyl]-2,2-dioxo-2,1,3-benzothiadiazol-5-yl}prop-2-enoate (20)

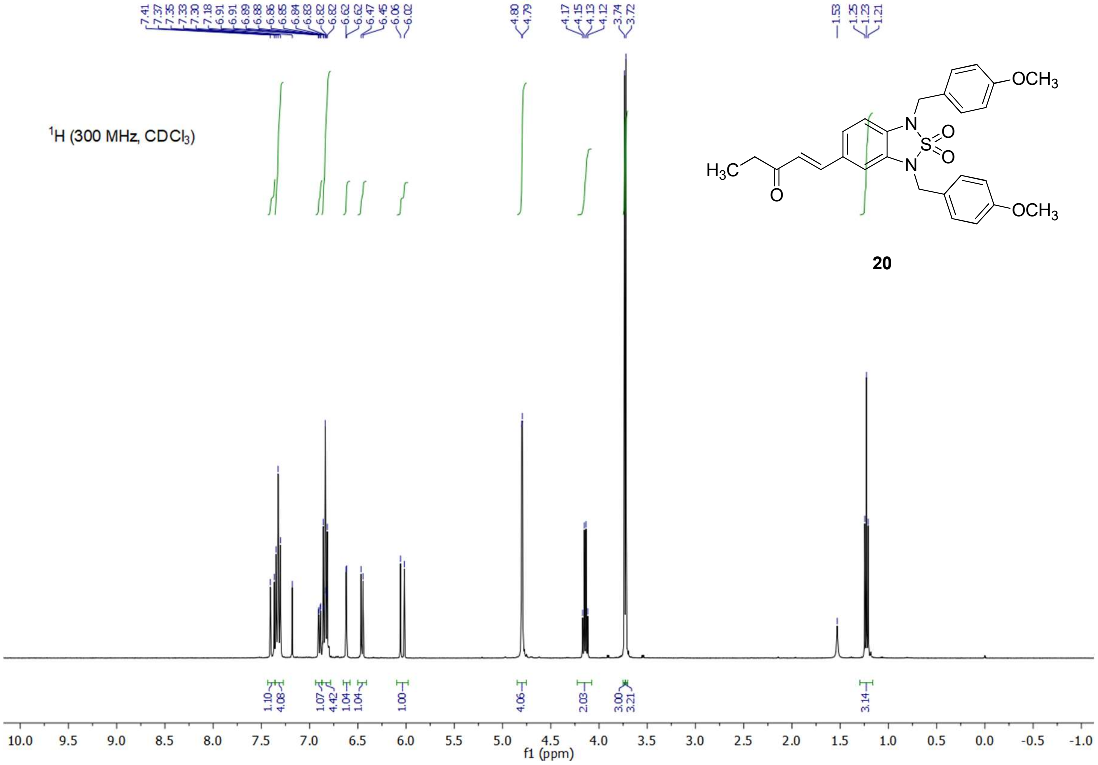

Figure S42. ^{1}H NMR spectrum of **20**.

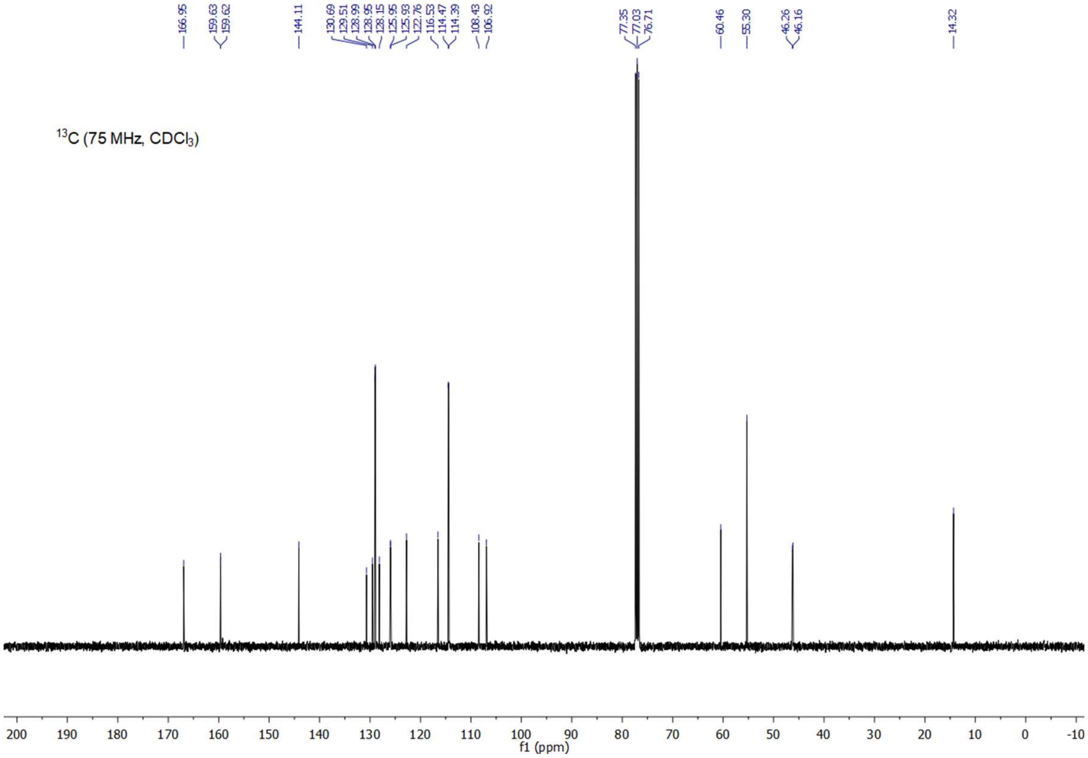

Figure S43. ^{13}C NMR spectrum of **20**.

S1.23. (2*E*)-3-{1,3-Bis[(4-methoxyphenyl)methyl]-2,2-dioxo-2,1,3-benzothiadiazol-5-yl}prop-2-enoic acid (21)

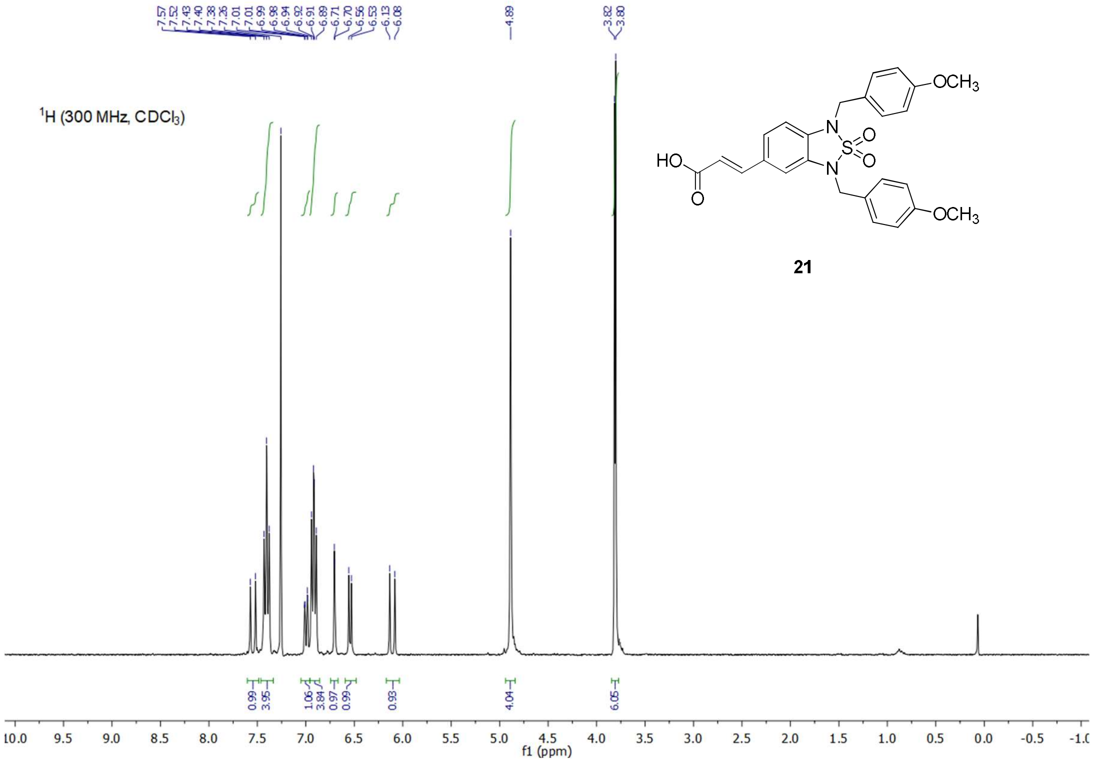

Figure S44. ^{1}H NMR spectrum of **21**.

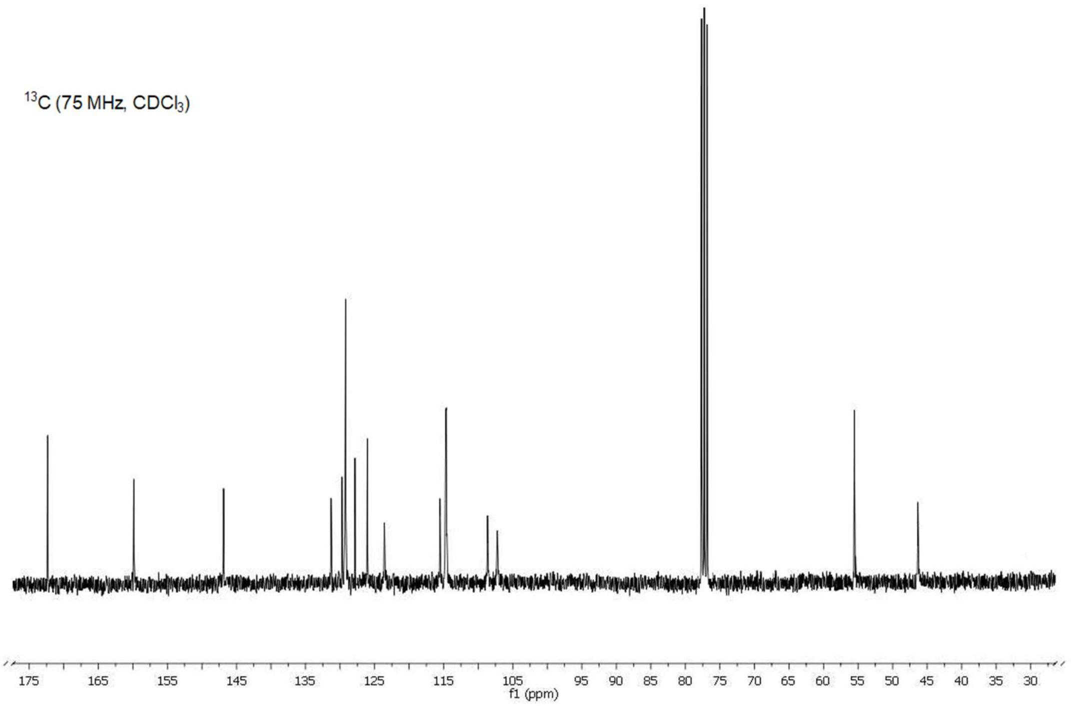

Figure S45. ^{13}C NMR spectrum of **21**.

S1.24. (2*E*)-3-{1,3-Bis[(4-methoxyphenyl)methyl]-2,2-dioxo-2,1,3-benzothiadiazol-5-yl}-*N*-(3-methylbutyl)prop-2-enamide (22)

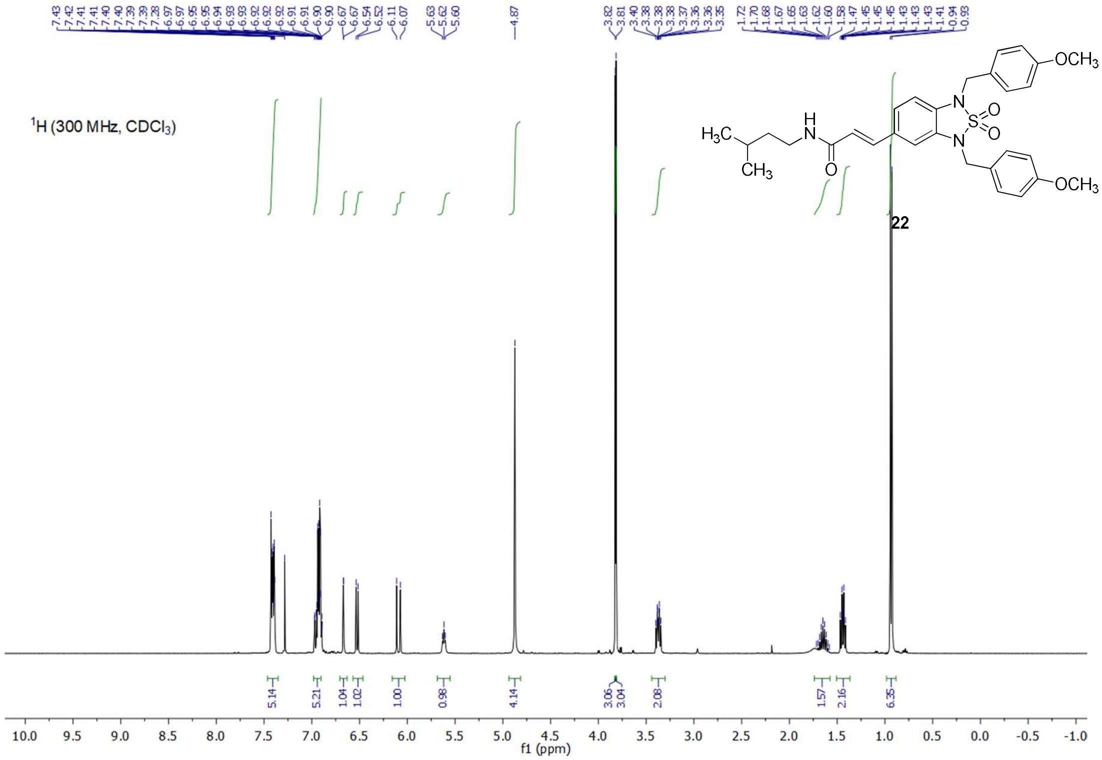

Figure S46. ^{1}H NMR spectrum of **22**.

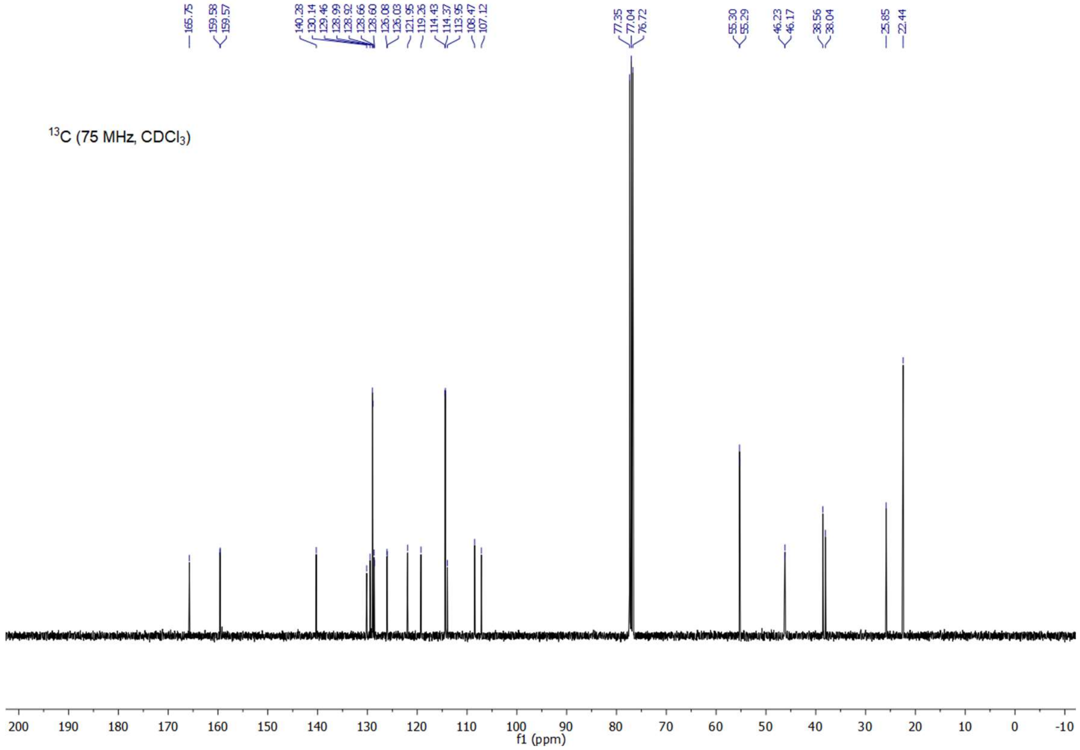

Figure S47. ^{13}C NMR spectrum of **22**.

S1.25. (2*E*)-3-{1,3-Bis[(4-methoxyphenyl)methyl]-2,2-dioxo-2,1,3-benzothiadiazol-5-yl}-*N*-phenylprop-2-enamide (23)

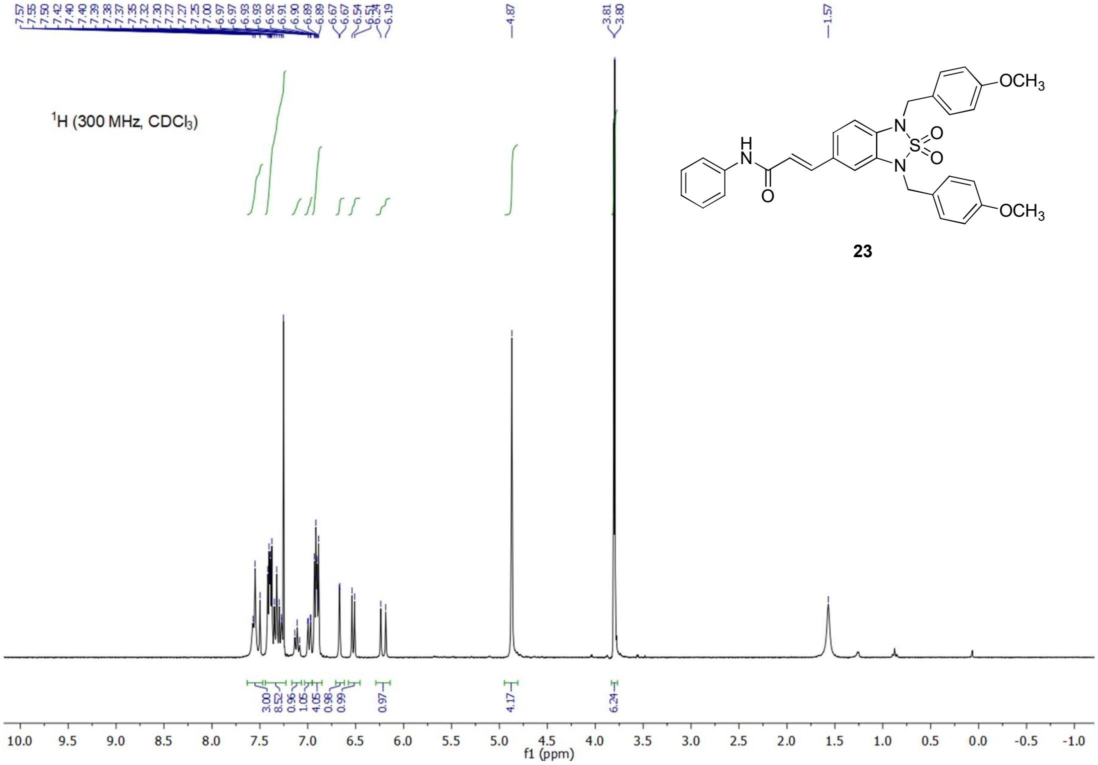

Figure S48. ^{1}H NMR spectrum of **23**.

S1.26. 3-(2,2-Dioxo-1*H*,3*H*-2,1,3-benzothiadiazol-5-yl)-*N*-(3-methylbutyl)propanamide (1g)

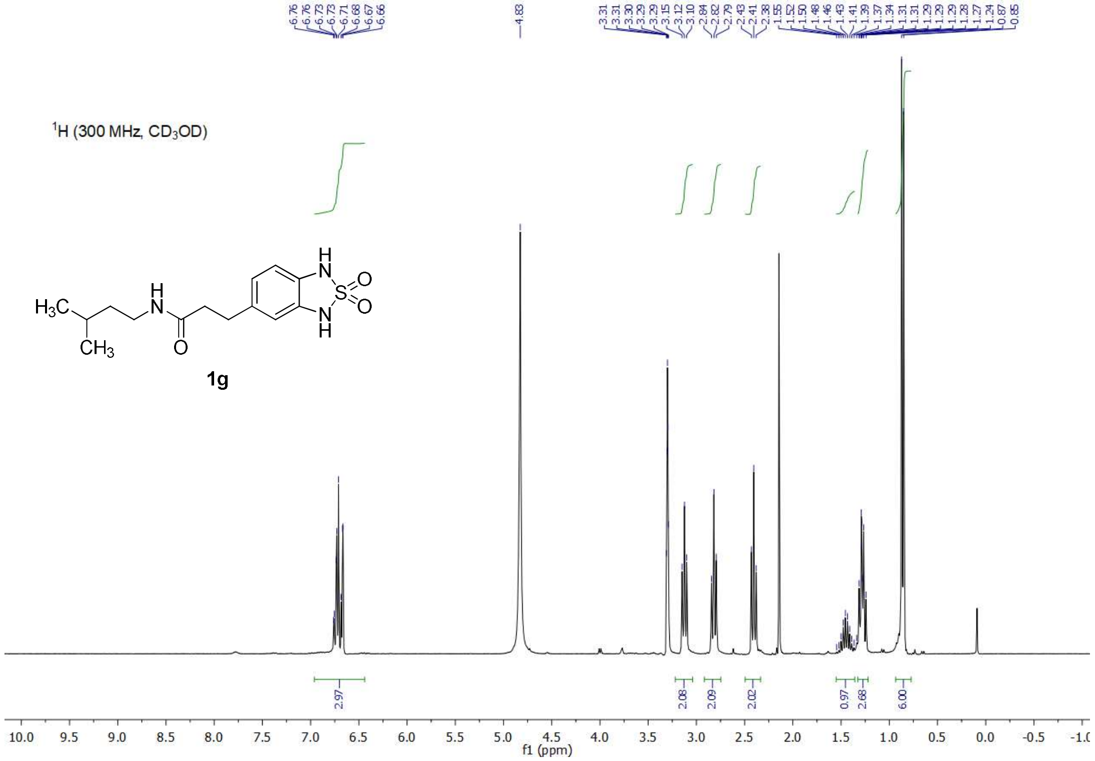

Figure S49. ^{1}H NMR spectrum of **1g**.

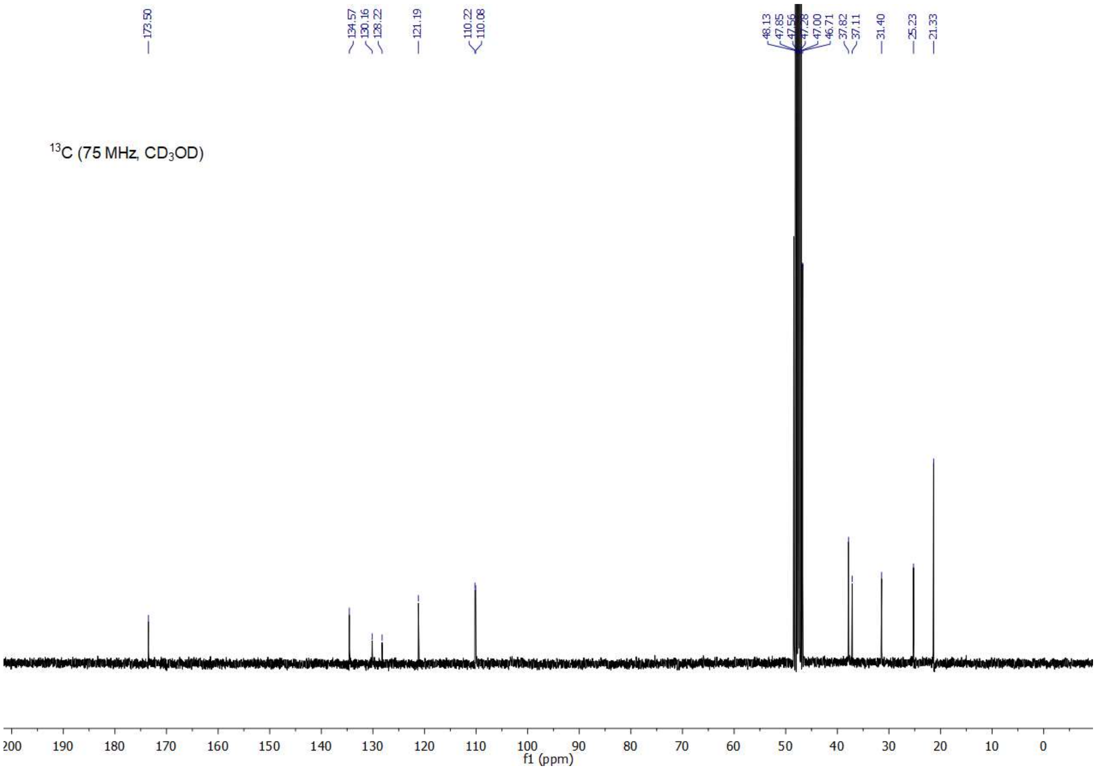

Figure S50. ^{13}C NMR spectrum of **1g**.

S1.27. 3-(2,2-Dioxo-1*H*,3*H*-2,1,3-benzothiadiazol-5-yl)-*N*-phenylpropanamide (1h)

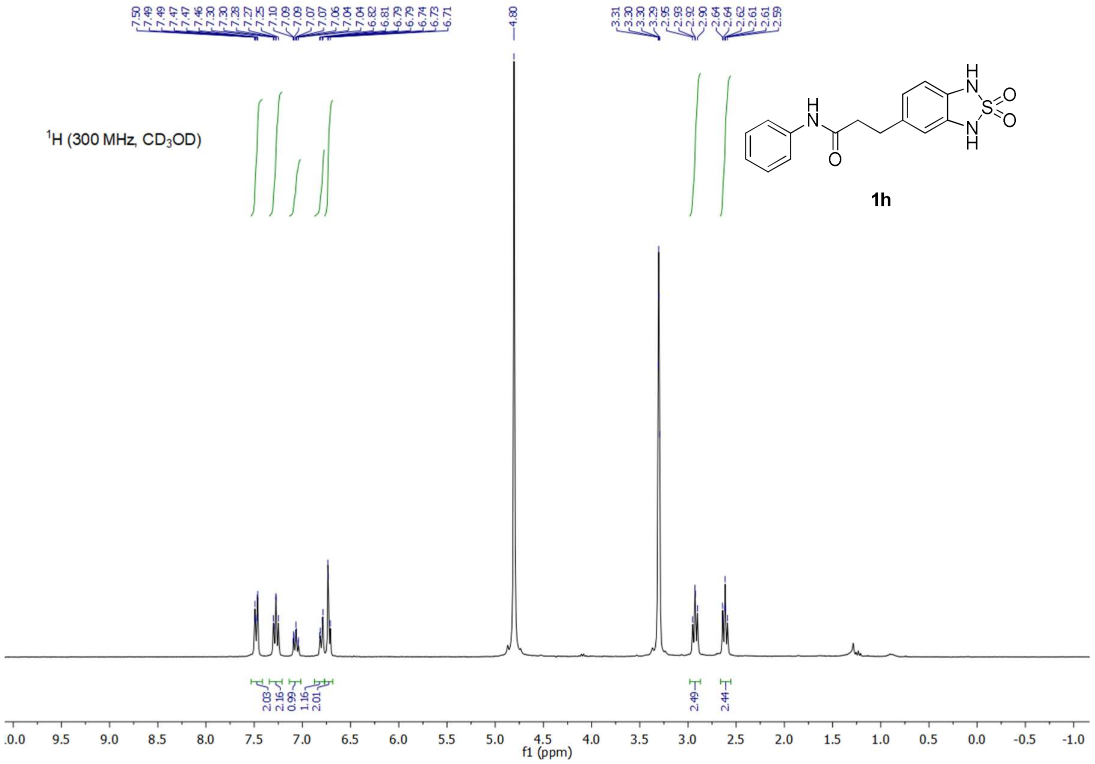

Figure S51. ^{1}H NMR spectrum of **1h**.

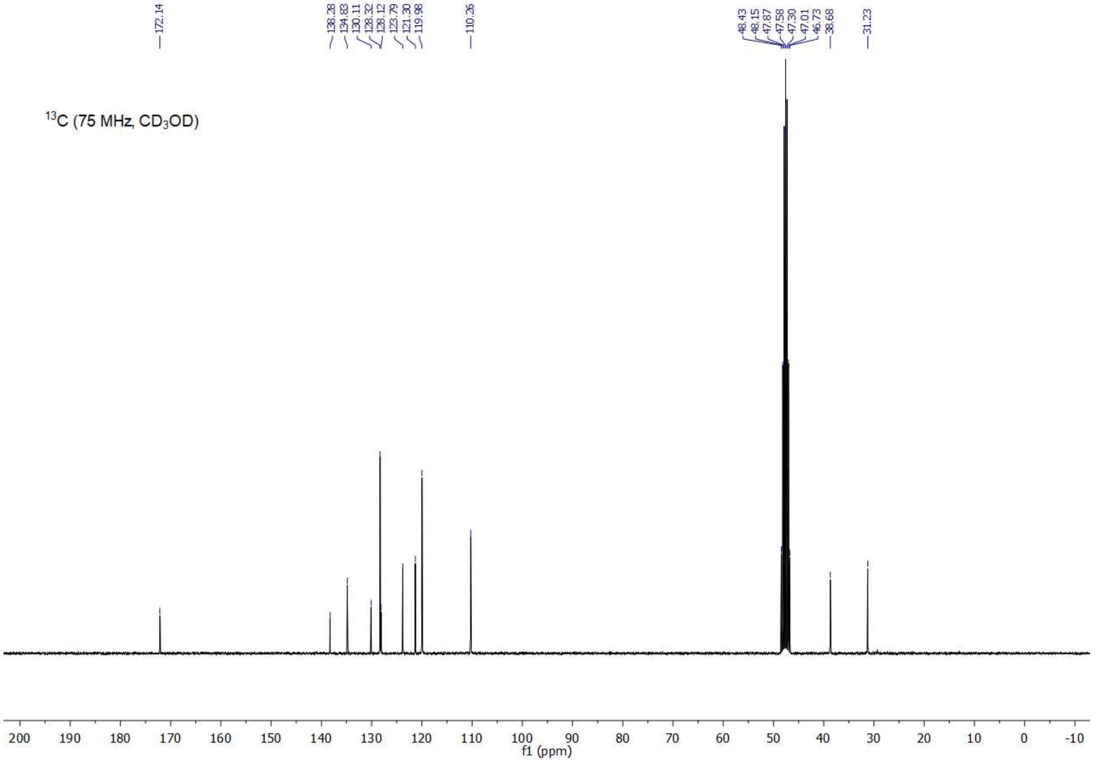

Figure S52. ^{13}C NMR spectrum of **1h**.

S1.28. **1,3-Bis[(4-methoxyphenyl)methyl]-5-phenyl-2,1,3-benzothiadiazole-2,2-dioxide (24)**

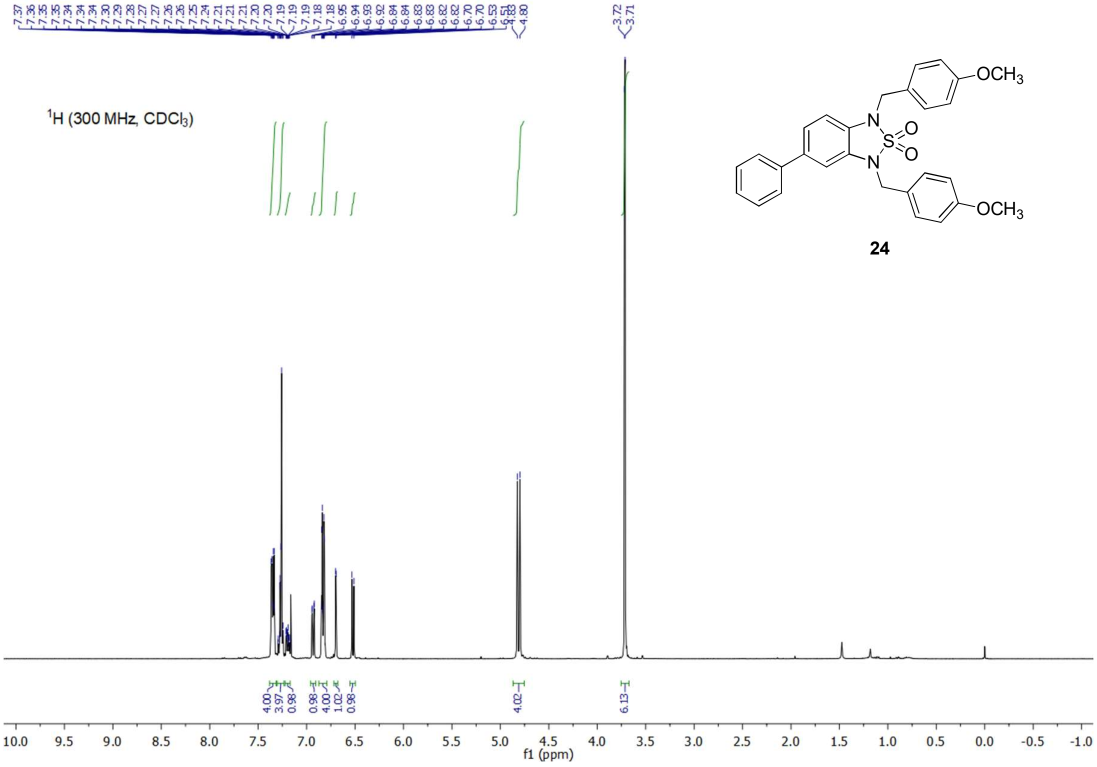

Figure S53. ^{1}H NMR spectrum of **24**.

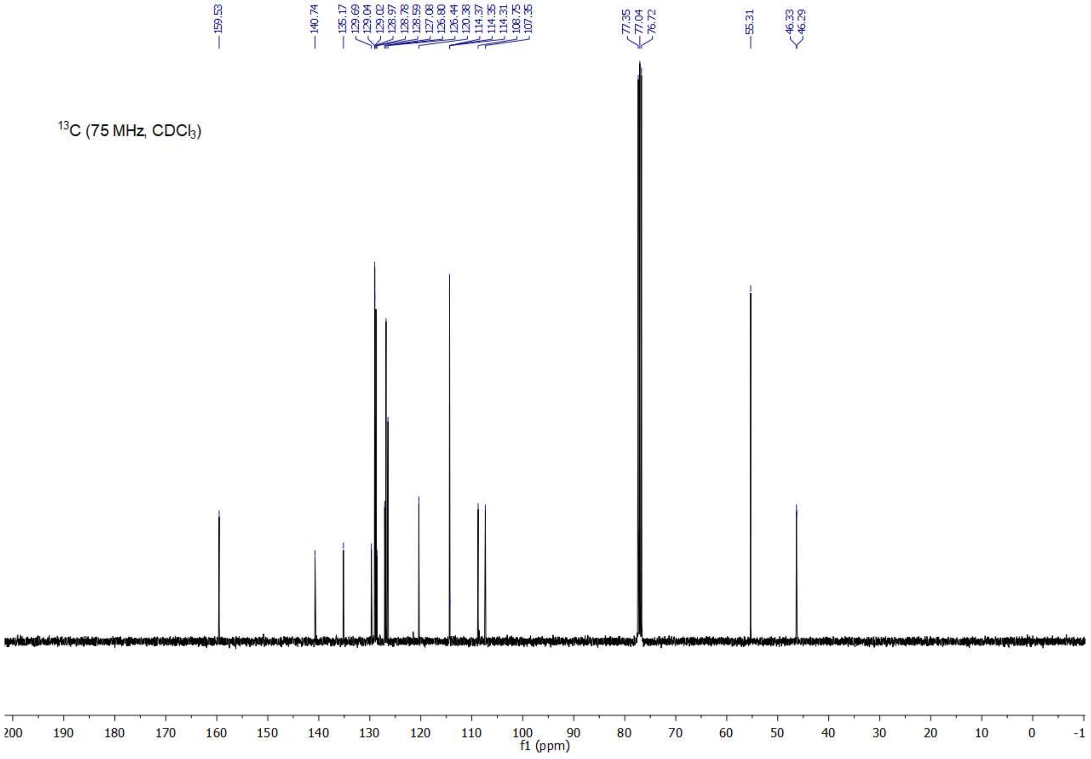

Figure S54. ^{13}C NMR spectrum of **24**.

S1.29. 5-Phenyl-1*H*,3*H*-2,1,3-benzothiadiazole-2,2-dioxide (1i)

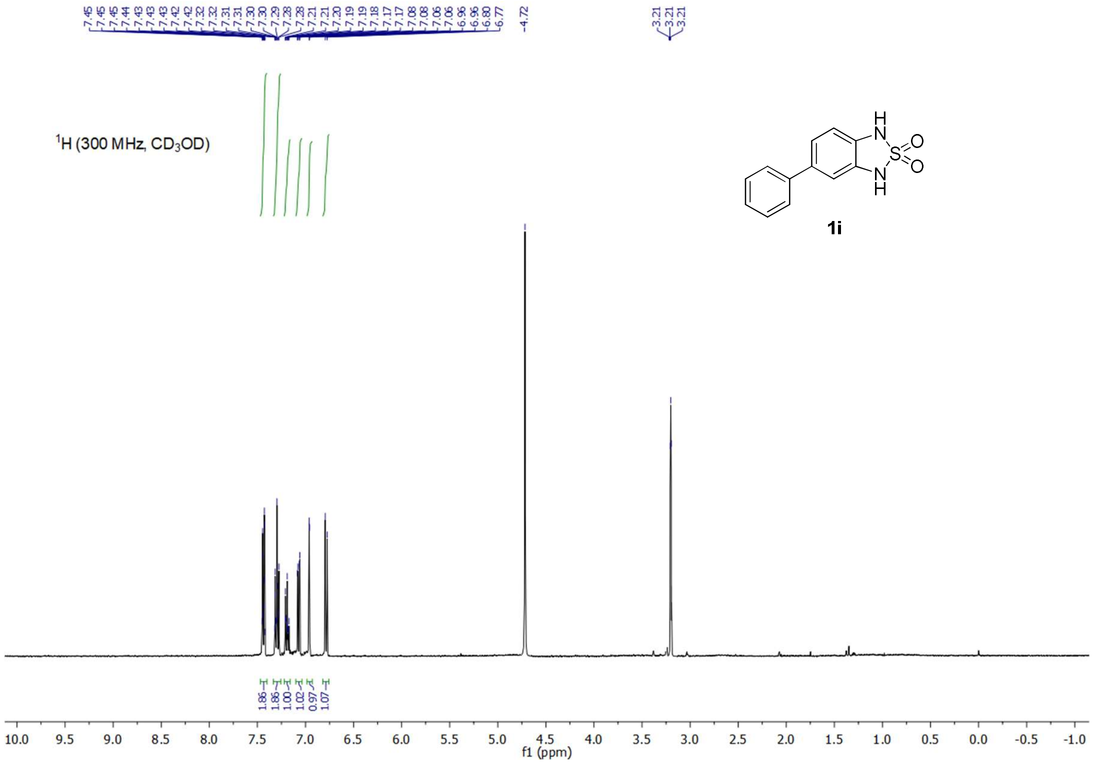

Figure S55. ^{1}H NMR spectrum of **1i**.

S1.30. 1,3-Dibenzyl-2,1,3-benzothiadiazole-2,2-dioxide (3)

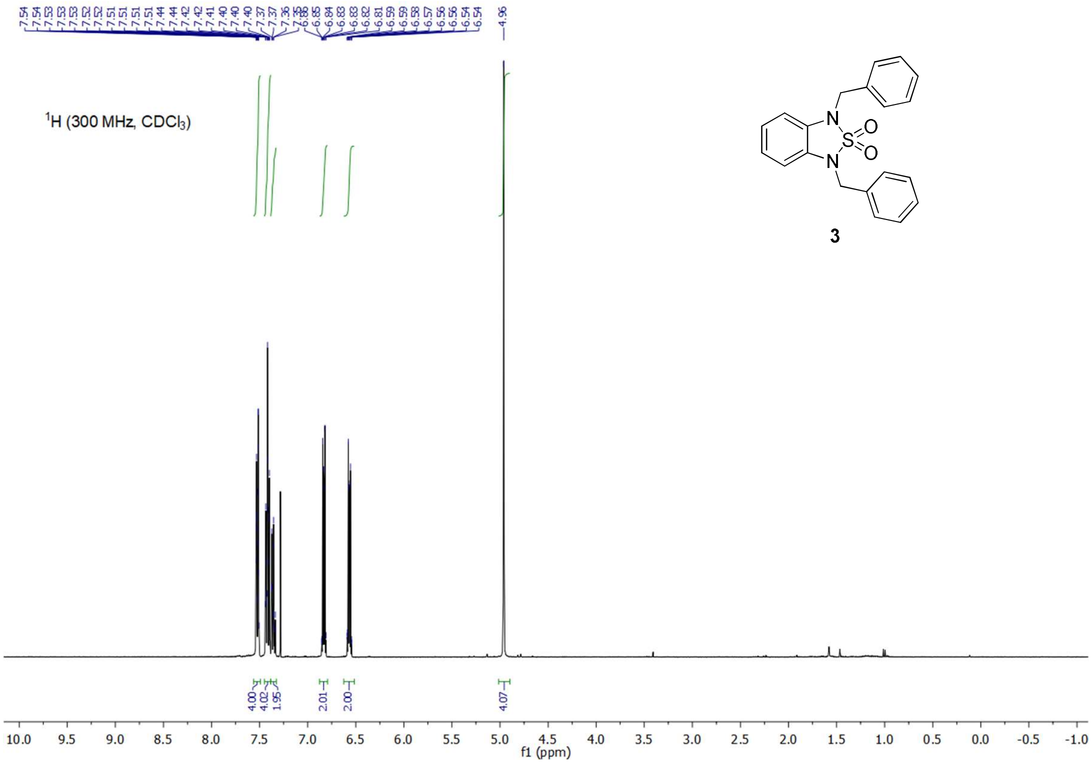

Figure S56. ^{1}H NMR spectrum of **3**.

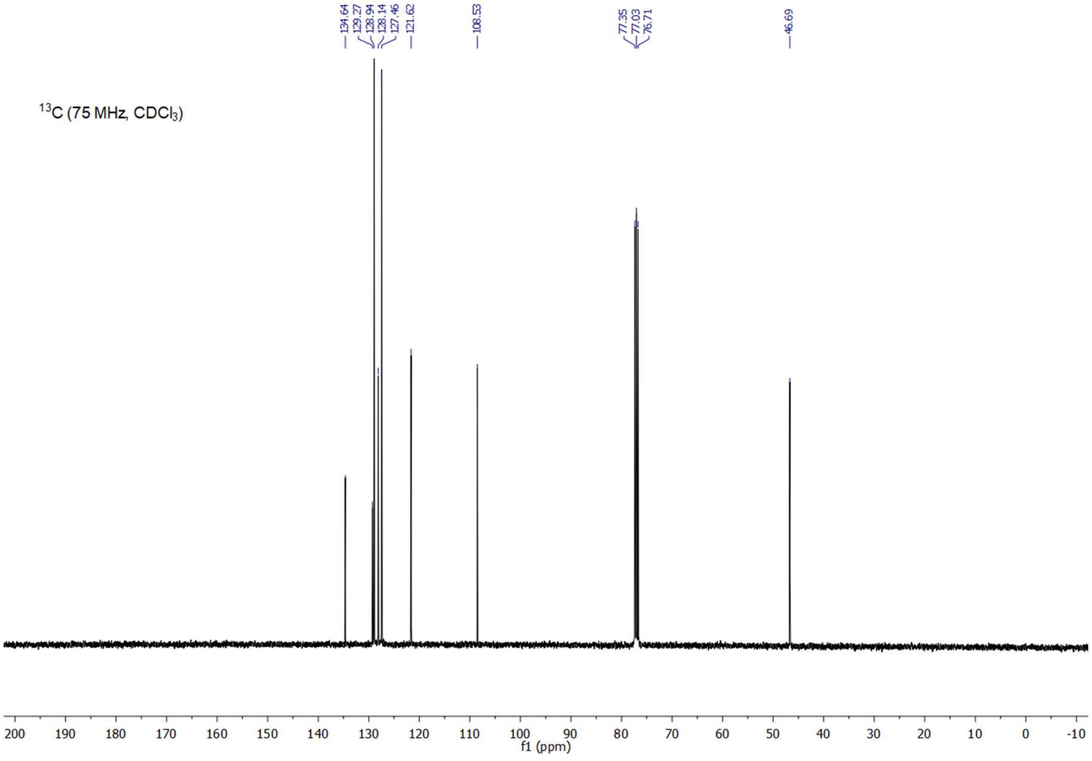

Figure S57. ^{13}C NMR spectrum of **3**.

S1.31. 1,3,5-Trimethyl-2,1,3-benzothiadiazole-2,2-dioxide (2b)

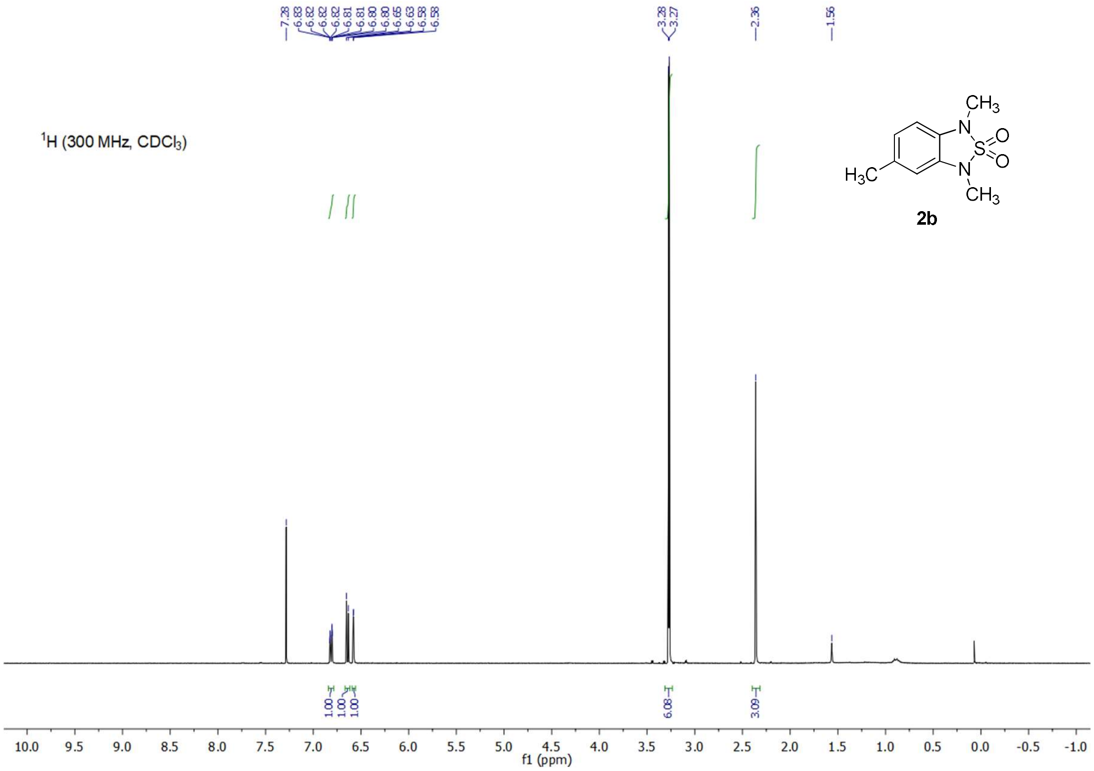

Figure S58. ^{1}H NMR spectrum of **2b**.

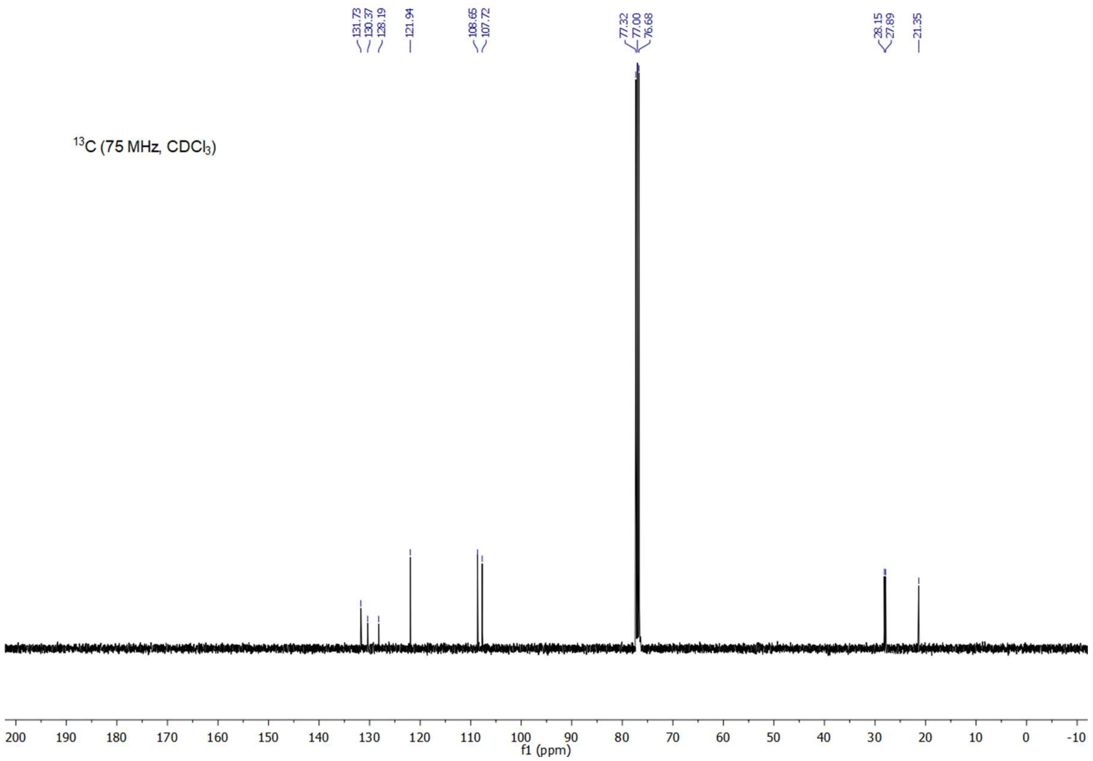

Figure S59. ^{13}C NMR spectrum of **2b**.

S1.32. *N*-[(2,4-Dimethoxyphenyl)methyl]-2-nitroaniline (25)

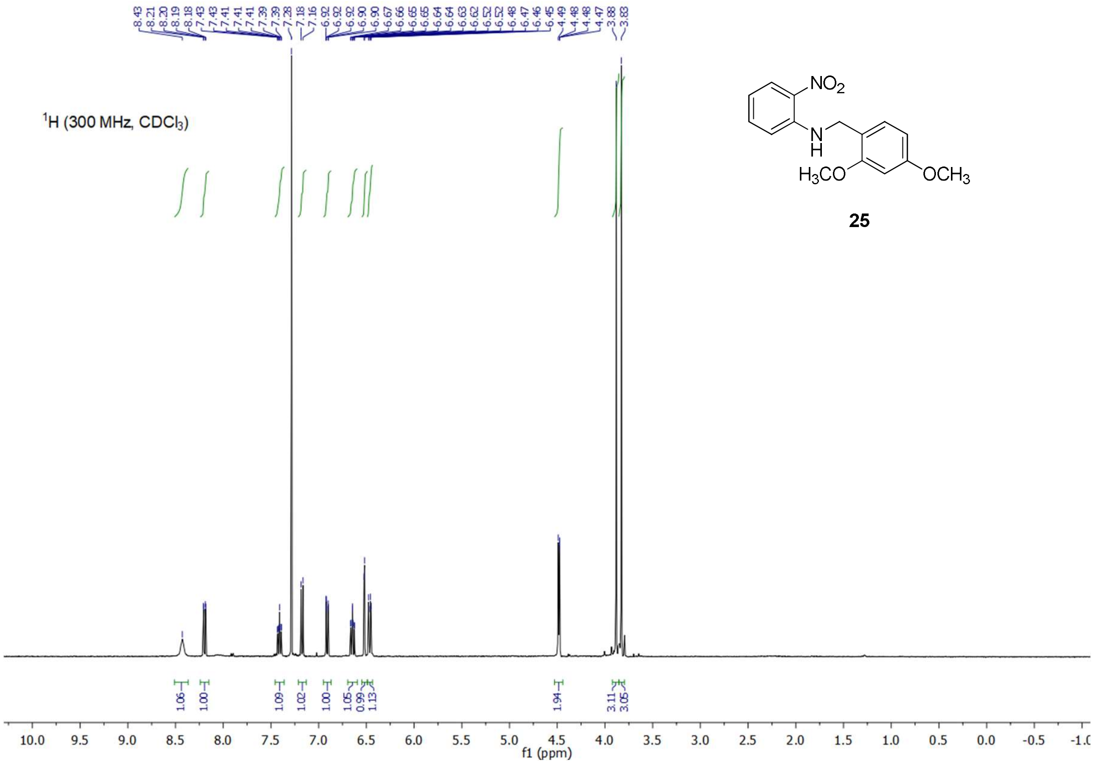

Figure S60. ^{1}H NMR spectrum of **25**.

S1.33. *N*1-[(2,4-Dimethoxyphenyl)methyl]benzene-1,2-diamine (26)

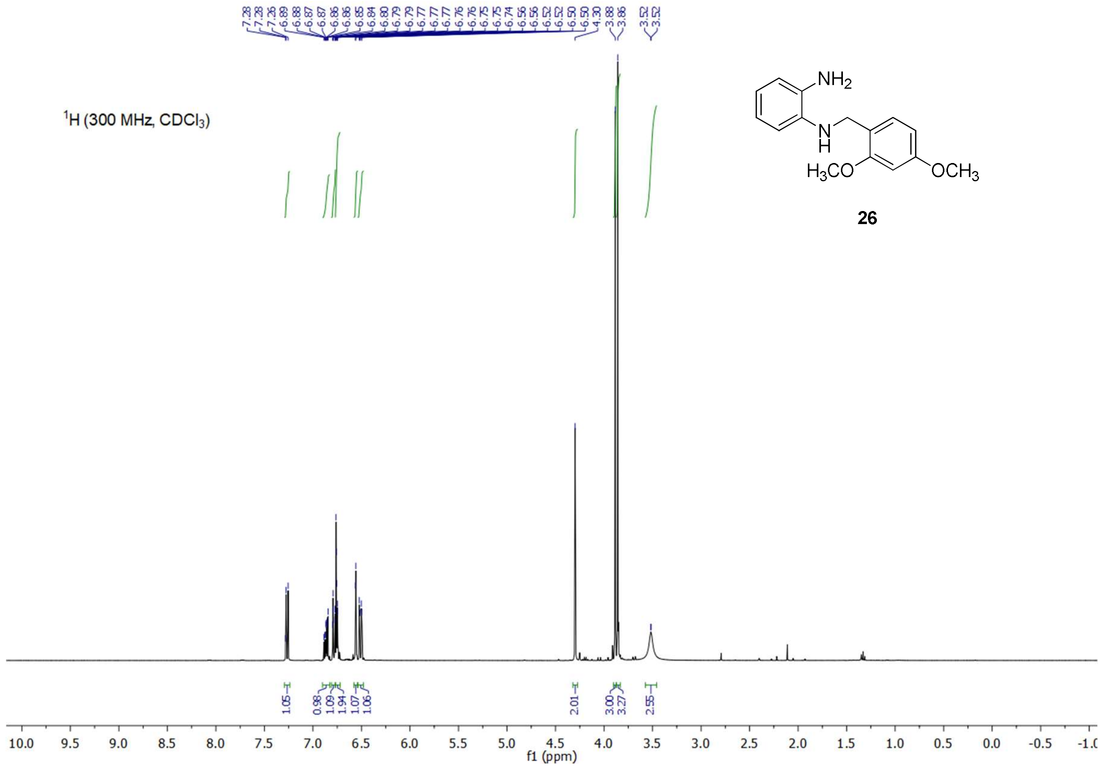

Figure S61. ^{1}H NMR spectrum of **26**.

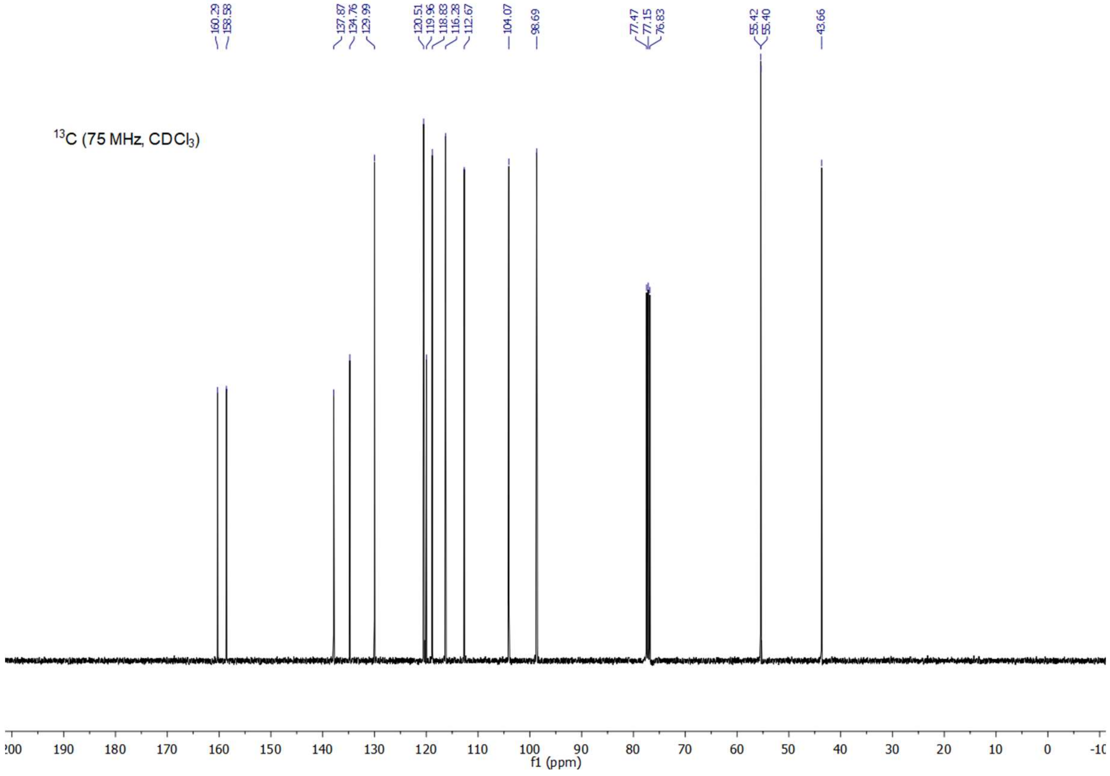

Figure S62. ^{13}C NMR spectrum of **26**.

S1.34. 1-[(2,4-Dimethoxyphenyl)methyl]-3*H*-2,1,3-benzothiadiazole-2,2-dioxide (27)

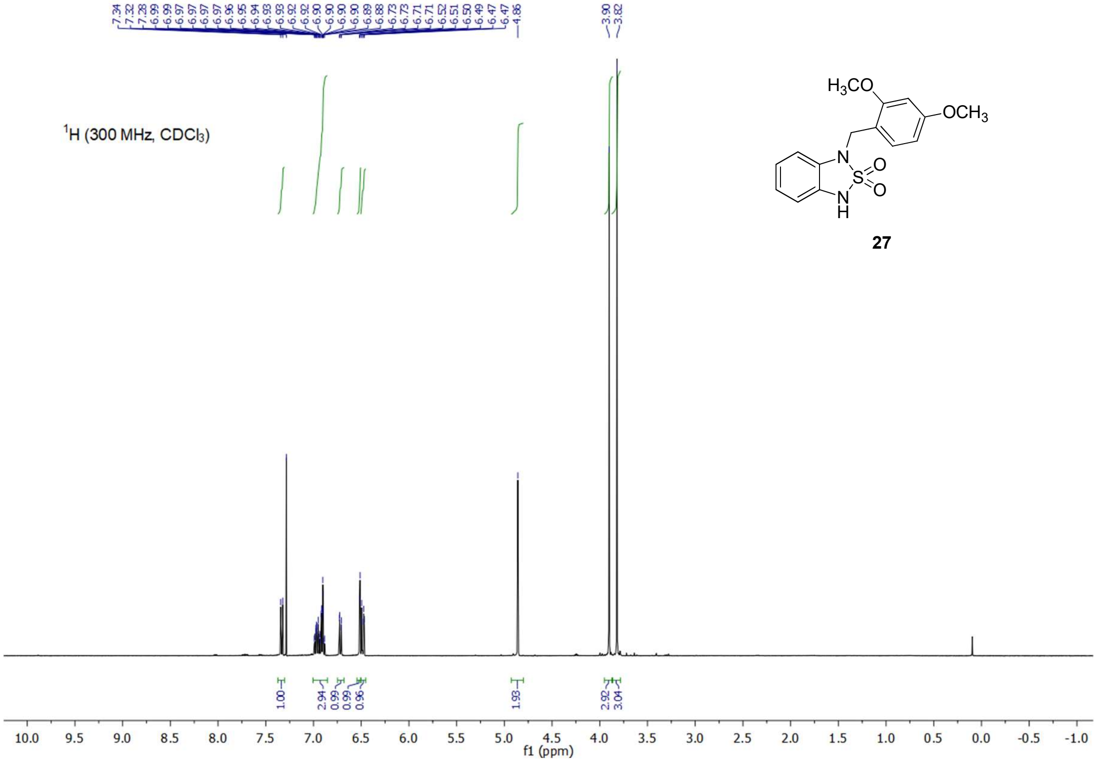

Figure S63. ^{1}H NMR spectrum of **27**.

S1.35. **Ethyl 2-{3-[(2,4-dimethoxyphenyl)methyl]-2,2-dioxo-2,1,3-benzothiadiazol-1-yl}acetate (28)**

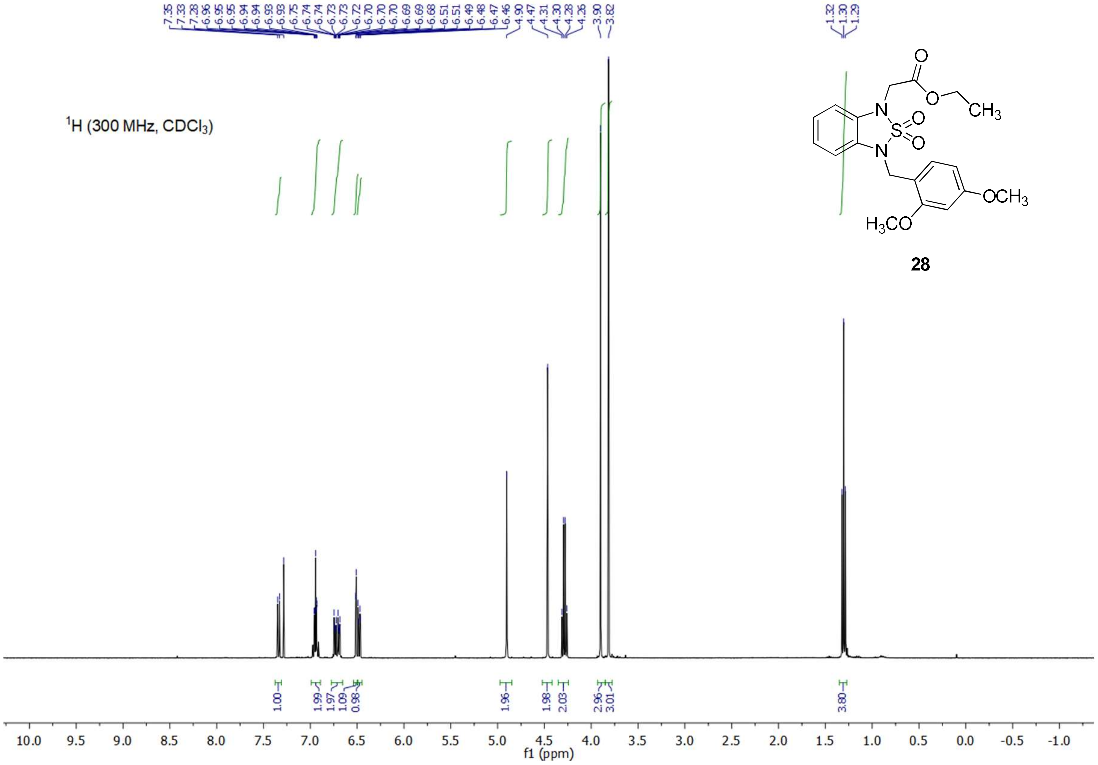

Figure S64. ^{1}H NMR spectrum of **28**.

S1.36. 2-{3-[(2,4-Dimethoxyphenyl)methyl]-2,2-dioxo-2,1,3-benzothiadiazol-1-yl} acetic acid (29)

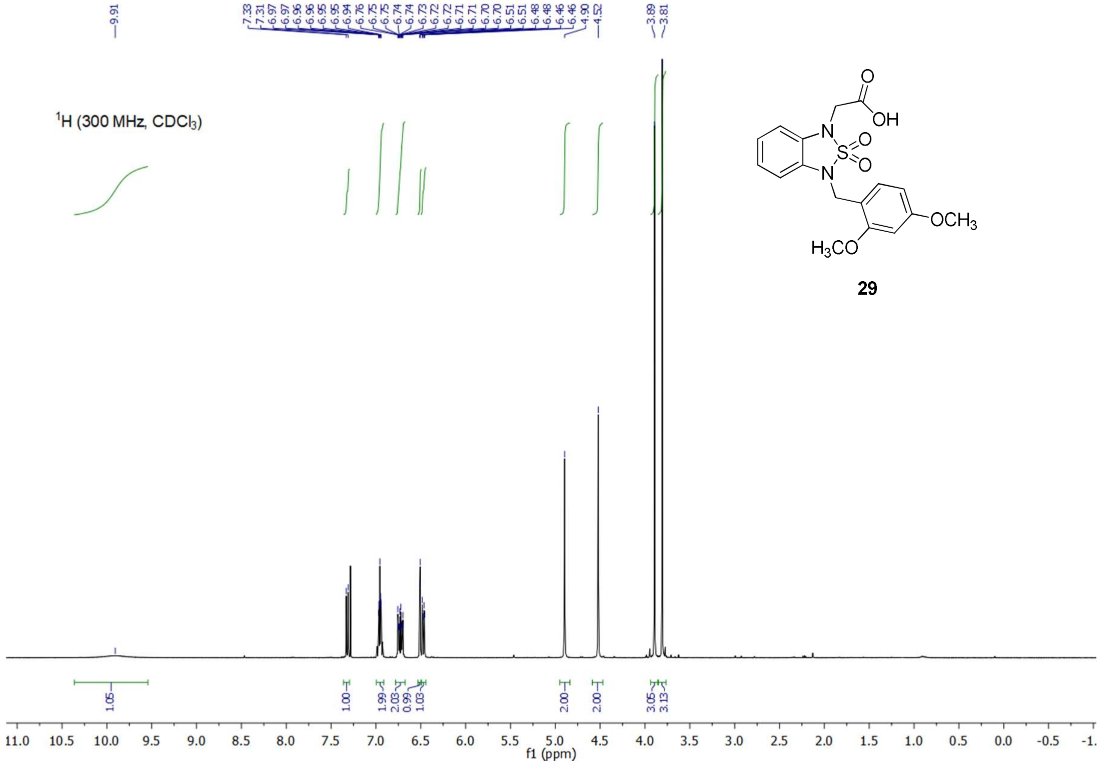

Figure S65. ¹H NMR spectrum of **29**.

S1.37. **2-{3-[(2,4-Dimethoxyphenyl)methyl]-2,2-dioxo-2,1,3-benzothiadiazol-1-yl}-*N*-phenethylacetamide (30)**

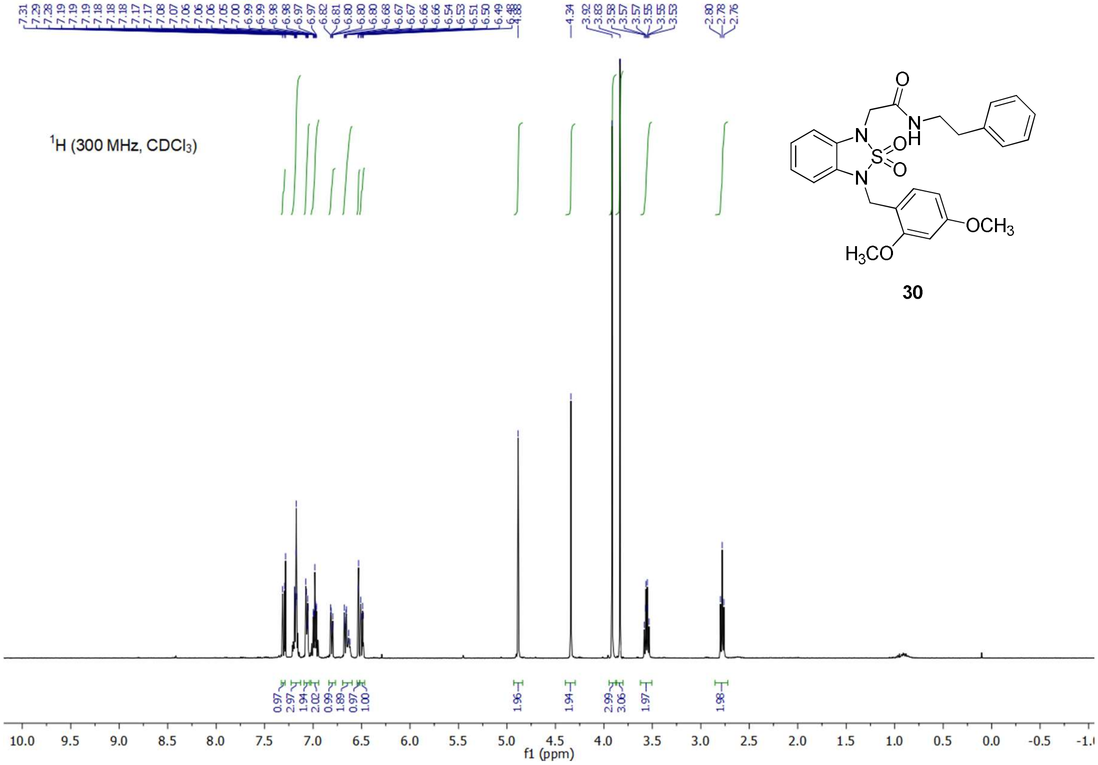

Figure S66. ^{1}H NMR spectrum of **30**.

S1.38. 2-(2,2-Dioxo-3*H*-2,1,3-benzothiadiazol-1-yl)-*N*-(2-phenylethyl)acetamide (4)

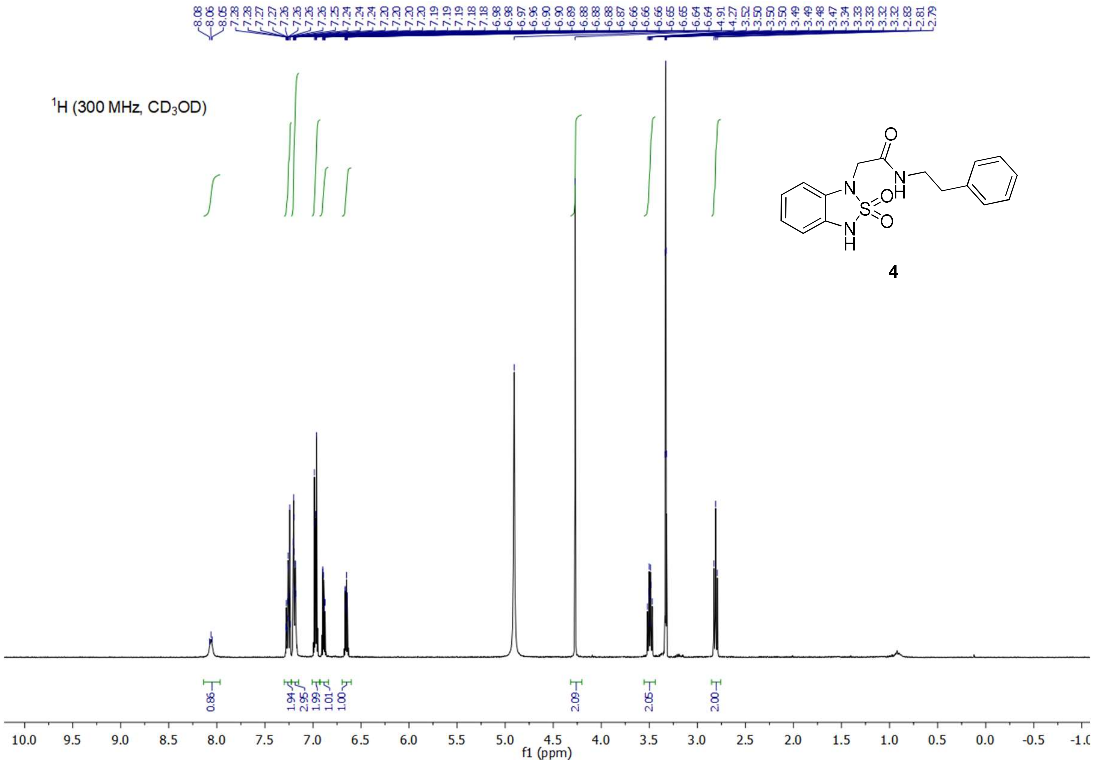

Figure S67. ^{1}H NMR spectrum of **4**.

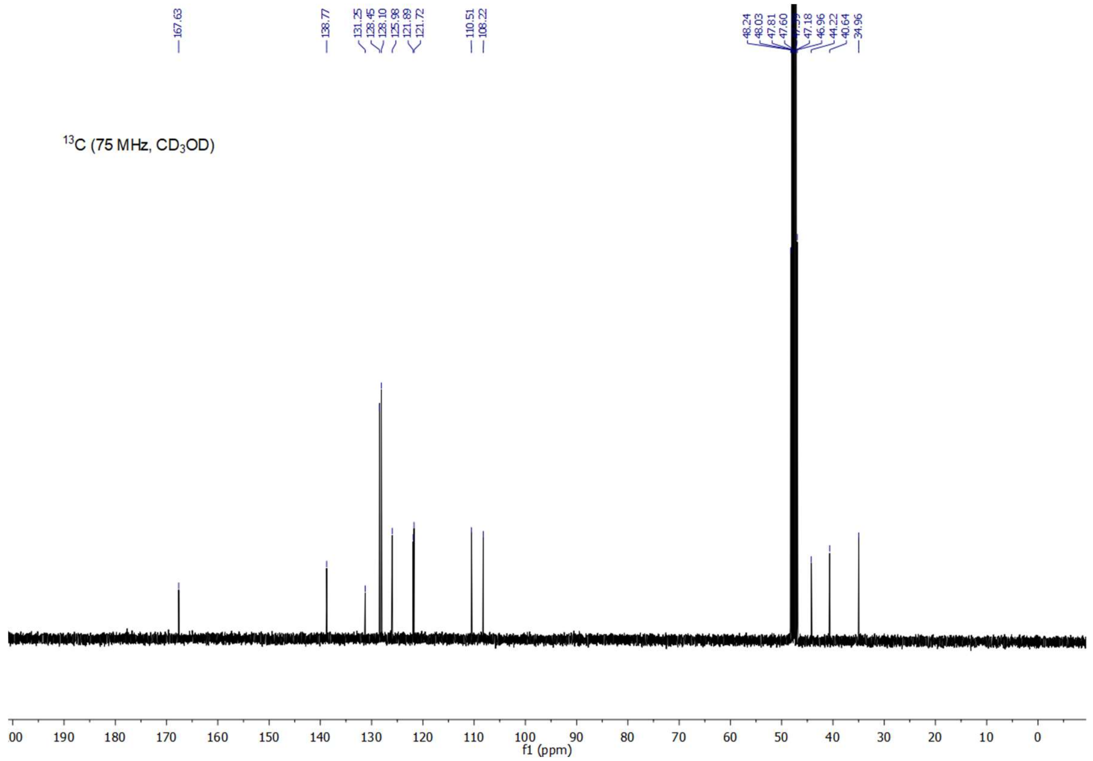

Figure S68. ^{13}C NMR spectrum of **4**.

S1.39. 2,3-Dihydro-1*H*-1,3-benzodiazol-2-one (5)

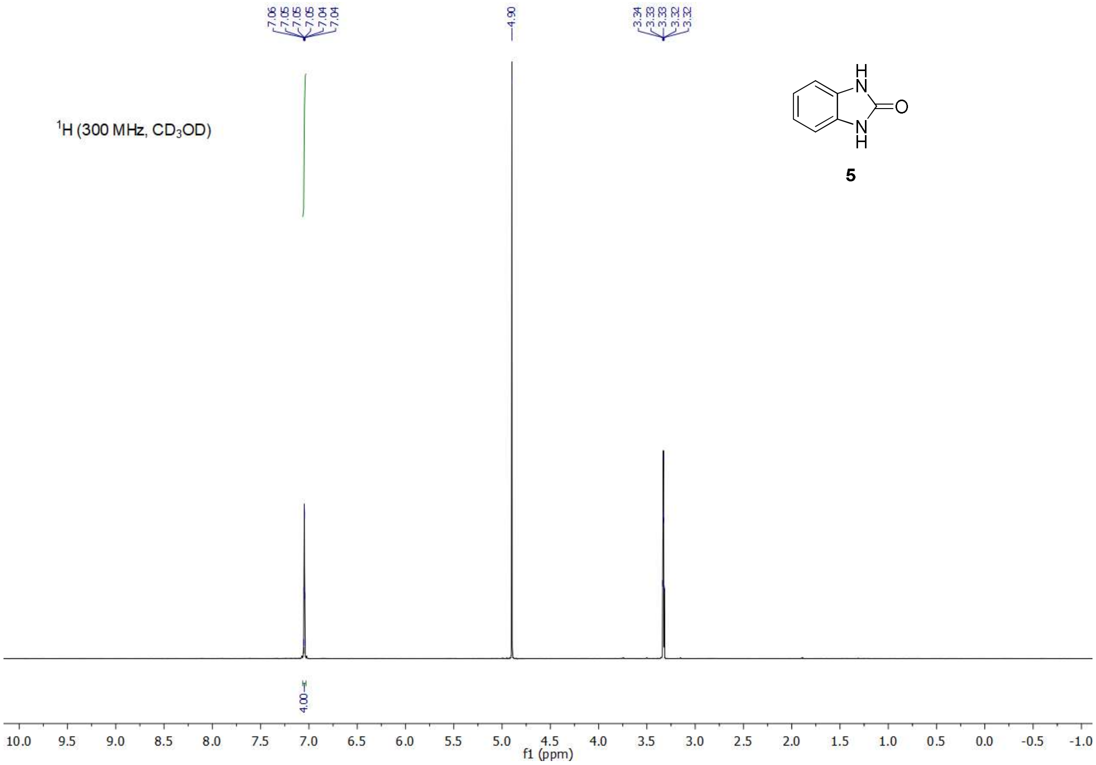

Figure S69. ^{1}H NMR spectrum of **5**.

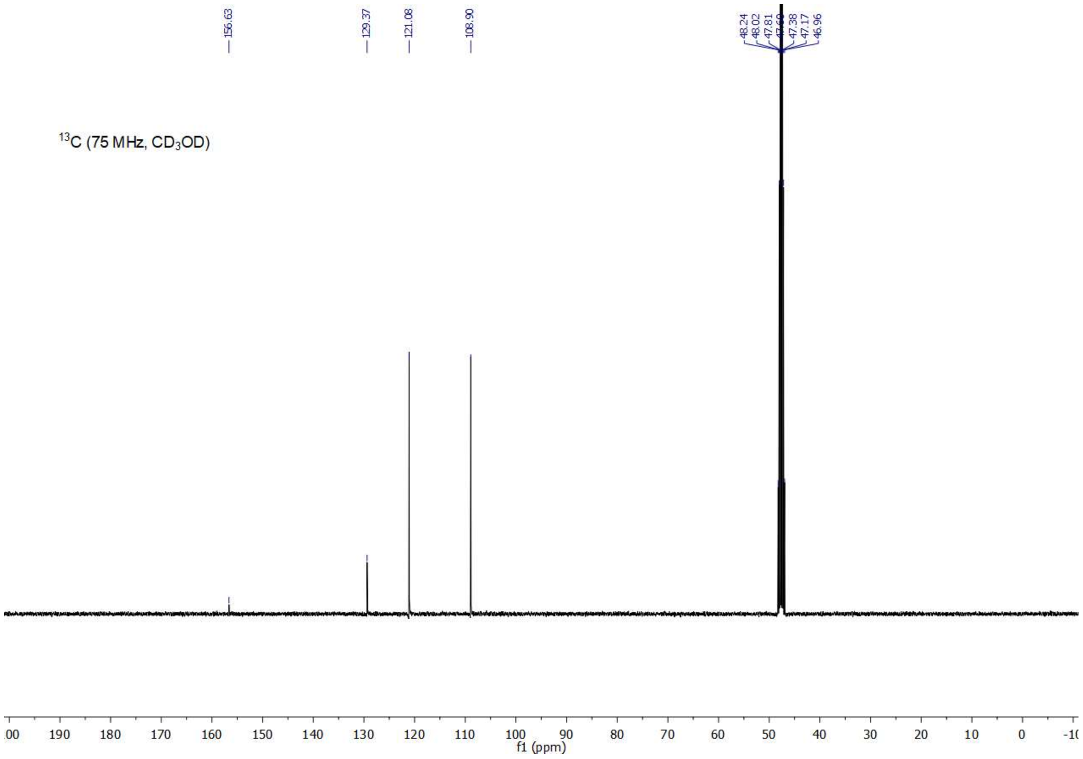

Figure S70. ^{13}C NMR spectrum of **5**..

Biological evaluation

Table S7. Effect of cysteine on the inhibitory activity of compound **1** *versus* STAT3.

		% of control	
		STAT3 AlphaScreen (DMSO: 5%)	
**Sample**	**[1] (μM)**	**2-Me or L-Cysteine 200 μM**	**IC_{50} (μM)**
**1** + 2-Me	0	131.3 ± 2.9	> 100
	0.1	138.3 ± 4.0	
	0.3	137.1 ± 2.3	
	1	140.9 ± 2.2	
	3	134.0 ± 3.7	
	10	136.8 ± 2.5	
	30	128.0 ± 1.6	
	100	111.7 ± 1.4	
**1** + L-Cysteine	0	101.8 ± 4.8	> 100
	0.1	103.9 ± 4.6	
	0.3	107.3 ± 5.0	
	1	107.8 ± 1.8	
	3	113.1 ± 4.6	
	10	115.0 ± 4.7	
	30	110.7 ± 5.7	
	100	101.9 ± 2.5	

Table S8. Effect of the mutation of cysteine residues located in the vicinities of the SH2 domain on the inhibitory activity of compound **1** *versus* STAT3. All concentrations are expressed in μM.

		% of control											
		STAT3 AlphaScreen (DMSO: 5%)											
**Spl**	**[1]**	**WT**	**IC_{50}**	**Cys468A**	**IC_{50}**	**Cys542A**	**IC_{50}**	**Cys550A**	**IC_{50}**	**Cys687A**	**IC_{50}**	**Cys712A**	**IC_{50}**
**1**	1	98.6 ± 0.9	15.8 ± 0.6	111.8 ± 5.9	> 30	102.5 ± 5.4	> 30	132.9 ± 15.1	> 30	93.3 ± 3.0	> 30	113.4 ± 9.8	> 30
	3	94.1 ± 3.6		108.3 ± 5.6		101.3 ± 4.9		136.0 ± 14.4		89.7 ± 5.3		112.4 ± 9.5	
	10	72.4 ± 2.6		101.1 ± 5.9		98.1 ± 4.1		135.2 ± 15.4		78.3 ± 4.2		110.5 ± 10.0	
	30	18.4 ± 0.4		79.7 ± 5.5		92.3 ± 4.4		124.7 ± 12.6		51.9 ± 1.9		108.0 ± 9.3	

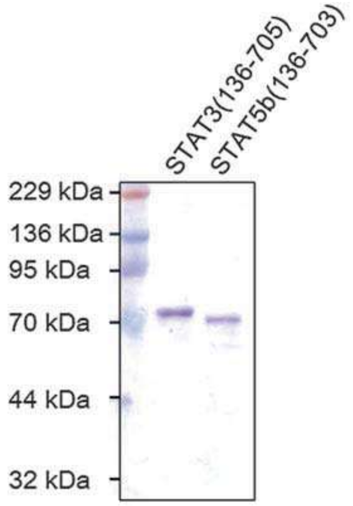

Figure S71. CBB staining of the STAT3(136–705) and STAT5b(136–703) proteins. The purified soluble proteins (0.5 μg) were analyzed by SDS-PAGE.†

†Asai, A.; Takakuma, K. Expression and Purification of Soluble STAT5b/STAT3 Proteins for SH2 Domain Binding Assay. In *SH2 Domains: Methods and Protocols*; Machida, K., Liu, B.A., Eds.; Springer: New York, 2017; pp. 163–172.

Computational studies on cysteine residues

Table S9. Comparison of the best STAT3 complexes obtained by docking compound **1** into the pockets containing the mutated cysteines, considered in the mutagenesis study. More in detail, the table shows the interaction energy between compound **1** and STAT3, evaluated by AutoDock 4, as well as the distances of the cysteine sulfur to the methyl at position 5 (CH_3---S) and the aromatic carbon at position 4 (CH---S), which were hypothesized to be the reactive centers.

**Cysteine**	**Energy (Kcal/mol)**	**CH_3---S (Å)**	**CH---S (Å)**
**468**	-4.72	7,99	8,92
**542**	-5,44	9,17	10,52
**550**	-3,52	3,64	5,13
**687**	-3,66	7,75	7,08
**712**	-5,93	4,70	4,95

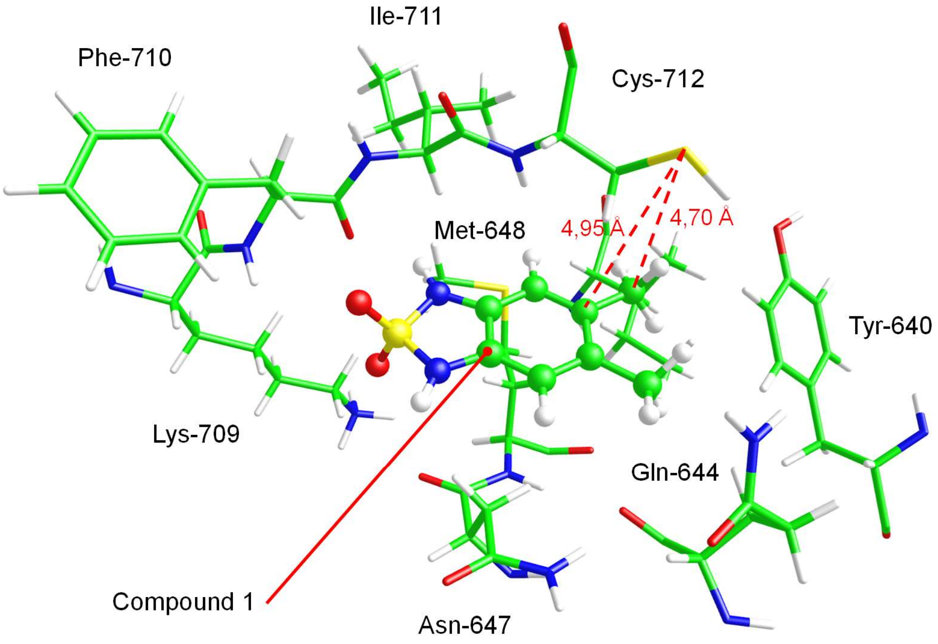

Figure S72. Main interactions of compound **1** into the pocket lined by Cys712. The complex is stabilized by a network of H-bonds involving the sulfone moiety and the backbone atoms of both Lys709 and Phe710. Moreover, the positively charged ammonium group of Lys709 interacts with the oxygen of the sulfone function. Both the methyl in 5 and the carbon in 4 seem well-oriented to react with the sulfur of Cys712.

^{1}H-NMR studies

The results are shown in Figures S72 and S73, in which the ^{1}H NMR spectra at time 0 (Figure S72) and after 24 h (Figure S73) are reported. Their comparison reveals that the spectrum of GSH changes after 24 h in the experimental condition used, showing two new signals at 4.08 (dd) and 4.18 (t) ppm. The intensities of these two signals were observed to increase over time (Figure S74). On the contrary, compound **1** alone did not show any modification of its spectrum after 24 h. Comparing spectra (2) and (3) of Figure S73, no additional significant signals appeared in the spectrum of the mixture (3) with respect to the GSH alone (2) after 24 h, suggesting that GSH did not bind to the methyl of compound **1** as in the **IVa** structure (Figure 8). In general, the spectrum of compound **1** mixed with GSH at 24 h (spectrum (3) of Figure S73) was similar to that at time 0 (spectrum (3) of Figure S72). On the contrary, the spectrum of GSH alone at 24 h (spectrum (2) of Figure S73) was different from that at time 0 (spectrum (2) of Figure S72), in particular for the different shape of the broad signal relative to the βCH_2Cys protons (2.75-2.95 ppm) and of the αCHGlu/αCH_2Gly multiplet (3.65-3-80 ppm), and for the chemical shift of αCHCys proton (4.42 ppm *vs* 4.45 ppm). Even if these differences in the spectra could suggest some interaction of compound **1** with GSH, the intensity of its aromatic CH (6.54 ppm) *vs* CH_3 (2.05 ppm) protons was roughly unchanged even after 6 days (Figure 11), apparently excluding the formation (at least in a detectable amount) of compound **IIb** (Figure 8), deriving from a covalent linkage between GSH and the aromatic ring of compound **1**. However, it should be considered that the different conditions employed for the NMR *vs* MS experiments (e.g. use of deuterated water as the solvent) may have reduced the extent of the proposed reaction. Hence, taking into account the lower sensitivity of NMR *vs* MS technique and the possibility that small signals could be masked by the superimposition of larger NMR resonances, we can conclude that the NMR outcomes cannot disprove the results obtained by MS spectroscopy.

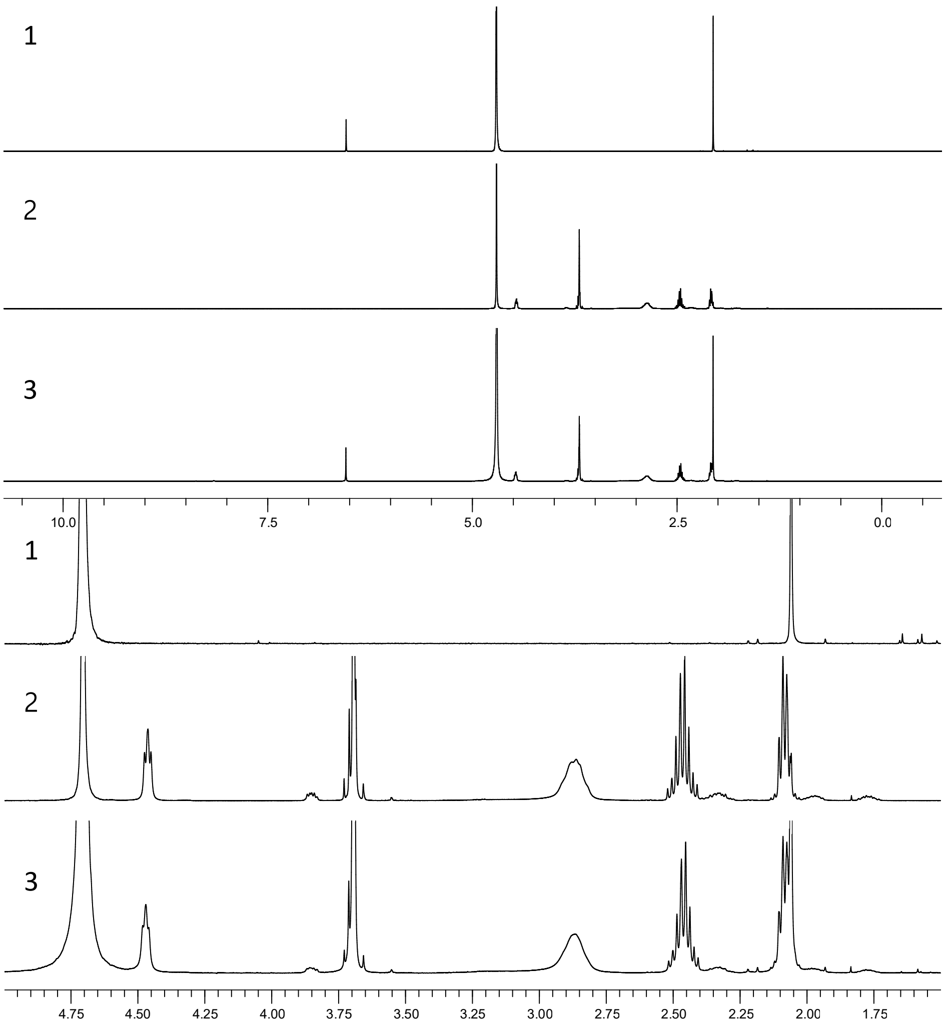

Figure S73. ^{1}H-NMR full spectra and expansion at time zero for: (1) compound **1** + $Na_2S_2O_5$; (2) GSH + $Na_2S_2O_5$; (3) compound **1** + $Na_2S_2O_5$ + GSH in D_2O carbonate buffer, pH ≈ 10. All spectra were recorded at 298 K.

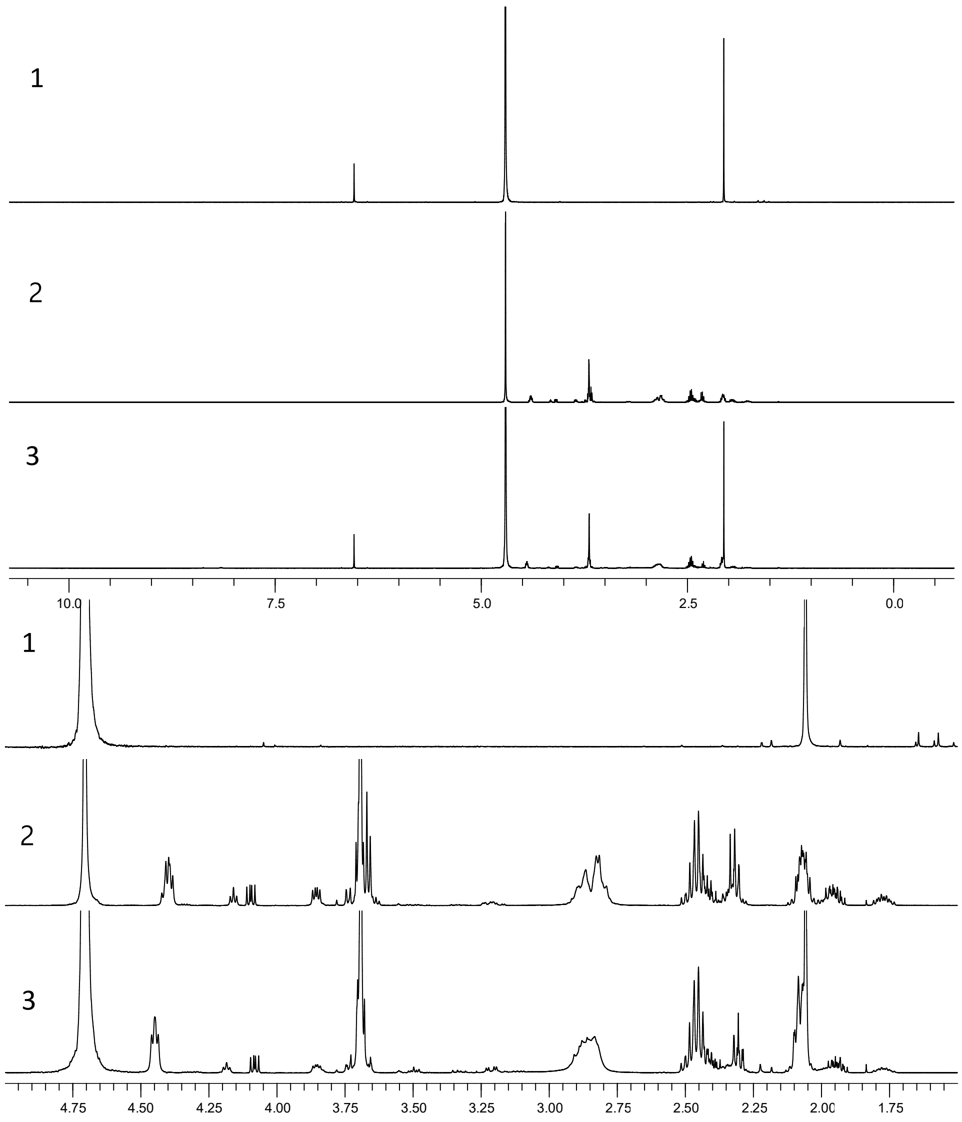

Figure S74. ^{1}H-NMR full spectra and expansion after 24 h at 37 °C: (1) compound **1** + $Na_2S_2O_5$; (2) GSH + $Na_2S_2O_5$; (3) compound **1** + $Na_2S_2O_5$ + GSH in D_2O carbonate buffer, pH ≈ 10. All spectra were recorded at 298 K.

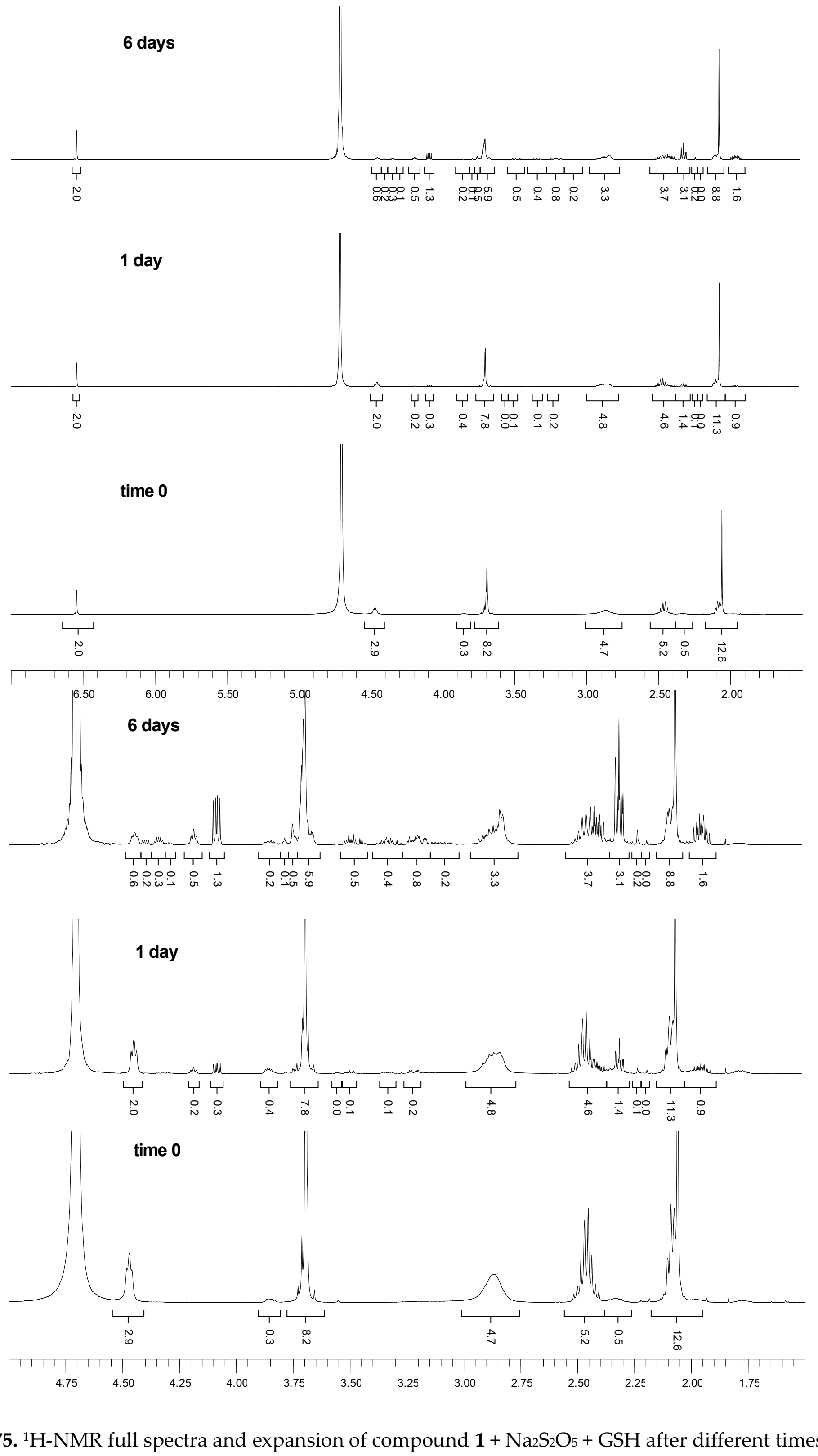

Figure S75. ^{1}H-NMR full spectra and expansion of compound **1** + $Na_2S_2O_5$ + GSH after different times at 37 °C and integrated signals.
